# Competence shut-off by intracellular pheromone degradation in salivarius streptococci

**DOI:** 10.1371/journal.pgen.1010198

**Published:** 2022-05-25

**Authors:** Adrien Knoops, Laura Ledesma-García, Alexandra Waegemans, Morgane Lamontagne, Baptiste Decat, Hervé Degand, Pierre Morsomme, Patrice Soumillion, Frank Delvigne, Pascal Hols

**Affiliations:** 1 Louvain Institute of Biomolecular Science and Technology, Université catholique de Louvain, Louvain-La-Neuve, Belgium; 2 Microbial Processes and Interactions, TERRA Research and Teaching Center, Gembloux Agro-Bio Tech, University of Liège, Gembloux, Belgium; Max Planck Institute for Terrestrial Microbiology: Max-Planck-Institut fur terrestrische Mikrobiologie, GERMANY

## Abstract

Competence for DNA transformation is a major strategy for bacterial adaptation and survival. Yet, this successful tactic is energy-consuming, shifts dramatically the metabolism, and transitory impairs the regular cell-cycle. In streptococci, complex regulatory pathways control competence deactivation to narrow its development to a sharp window of time, a process known as competence shut-off. Although characterized in streptococci whose competence is activated by the ComCDE signaling pathway, it remains unclear for those controlled by the ComRS system. In this work, we investigate competence shut-off in the major human gut commensal *Streptococcus salivarius*. Using a deterministic mathematical model of the ComRS system, we predicted a negative player under the control of the central regulator ComX as involved in ComS/XIP pheromone degradation through a negative feedback loop. The individual inactivation of peptidase genes belonging to the ComX regulon allowed the identification of PepF as an essential oligoendopeptidase in *S*. *salivarius*. By combining conditional mutants, transcriptional analyses, and biochemical characterization of pheromone degradation, we validated the reciprocal role of PepF and XIP in ComRS shut-off. Notably, engineering cleavage site residues generated ultra-resistant peptides producing high and long-lasting competence activation. Altogether, this study reveals a proteolytic shut-off mechanism of competence in the salivarius group and suggests that this mechanism could be shared by other ComRS-containing streptococci.

## Author summary

The human oral cavity is one of the most challenging ecological niches for bacteria. In this ecosystem, hundreds of species compete for food and survival in a physicochemical fluctuating environment. To outcompete, S*treptococcus salivarius* has developed a particular physiological state called competence during which antibacterial compounds are produced together with the uptake of external DNA that can be integrated in its own genome. Although this strategy is of main importance for evolution and adaptation, its short-term cost in terms of energy and metabolism reprogramming are important. To restrain competence activation to a sharp window of time, bacteria use a process known as shut-off. Although described in some species, this process is still mostly unknown in streptococci. In this work, we used predictive mathematical simulations to infer the role of a pheromone-degradation machinery involved in the exit from competence. We confirmed experimentally this mechanism by identifying PepF as a competence-induced oligoendopeptidase with a specific activity towards the XIP pheromone. Importantly, we show that this peptidase is not only shutting down competence but also preventing its development under inappropriate conditions.

## Introduction

The human microbiota that includes *Streptococcus salivarius* is one of the most competitive and challenging microbial ecosystems. Limited resources, physicochemical inconstancy and bacterial high density have fostered the emergence of powerful survival strategies [1–3]. Sophisticated regulation networks ensure a tight control over those tactics, converting miscellaneous environmental inputs to population-wide synchronized outcomes [4–6]. Orchestrating coordinated bacteriocin production and natural transformation, competence in streptococci is a glaring illustration of such a ploy. While the production of antimicrobial compounds eliminates competitors, rival cell lysis guarantees genomic material availability for subsequent DNA uptake [7–9].

In streptococci, competence activation relies on two distinct pheromone-dependent mechanisms. In both cases, initiation occurs through the production of a genome-encoded small peptide, acting as a pheromone after secretion and maturation [10]. In two streptococcal groups (i.e. mitis and anginosus), the competence signaling peptide (CSP) sparks a phosphorelay after binding to its cognate histidine kinase ComD, ultimately stimulating dimerization of the transcriptional factor ComE and its transcriptional activity [11,12]. On the other hand, all other streptococci groups (i.e. mutans, bovis, pyogenes, suis, and salivarius) operate with a differing pattern [10]. The pheromone precursor ComS is secreted through the PptAB ABC transporter [13], maturated to form XIP (ComX-Inducing-Peptide), and then internalized by the generic oligopeptide transporter Ami/Opp [14,15]. Subsequent binding to the intracellular receptor ComR triggers dimerization of the complex ComR•XIP, leading to the establishment of a positive feedback loop via *comS* transcriptional activation [16]. Concomitantly, ComR•XIP will trigger the production of the alternative sigma factor ComX which will guide RNA polymerase to promoters of late competence genes responsible for the transformasome assembly [10].

During the past two decades, the accumulation of genomic data together with multi-species investigations of DNA exchange reshaped our perception of competence as a major driving force in adaptation and evolution of streptococci [17–21]. Even though competence long-term benefits for bacteria are undeniable, the process is energetically expensive, reprograms deeply the metabolism, and transitory impairs the regular cell-cycle [22,23]. To curtail this dramatic impact on the survival chance of the population, streptococci exploit complex regulatory networks to restrict competence activation to a subpopulation. This tactic known as bimodality or excitability (since transitory) allows only a subset of the community to play the dangerous but yet promising game of competence [5,24]. An auxiliary strategy aims to narrow the activation time window of this peculiar physiological state to restore routine cell-cycle once DNA has been internalized and integrated. This mechanism termed competence shut-off, has been thoroughly described in *Streptococcus pneumoniae*, where at least two distinct mechanisms were highlighted. The first one involves the late competence protein DprA, the RecA loader involved in single-stranded DNA recombination during transformation [25]. Strikingly, this protein was shown to have a supplementary function in competence shut-off by directly interacting with ComE~P, thereby interrupting the positive feedback loop acting on the *comCDE* operon [26,27]. Moreover, this mechanism was shown to require ComX to spatially coordinate the interaction between DprA and ComE~P at a single cell pole [28]. Another mechanism was suggested by Martin and co-workers, showing that ComE accumulation due to the activation of the *comCDE* operon would compete with ComE~P for its binding to the *comCDE* promoter, ultimately leading to competence shut-off [29].

In ComRS-streptococci, a number of competence negative players have been depicted. Among them, the endopeptidase PepO was shown in *Streptococcus mutans* to control XIP abundance. However, since *pepO* transcription is not stimulated through competence, the endopeptidase would rather stand as a locking device than a proper shut-off player [30]. Two other antagonist mechanisms controlled by competence and thereby acting through a negative feedback loop were described in streptococci. The small protein paratox (Prx) from a prophage of *Streptococcus pyogenes* was shown to be controlled by ComX and to prevent ComR-XIP association [31,32]. An analogous model in *S*. *mutans* involves a peptide termed XrpA that interferes with ComR dimerization [33]. Surprisingly, this peptide is encoded within the *comX* coding sequence and therefore is upregulated along with competence activation [34]. Although those two ComRS antagonists are rooted in a negative feedback loop, their inactivation was not shown to extend the competence time window [31,35], suggesting they do not stand as true shut-off effectors.

In salivarius streptococci, competence shutdown remains unexplained. Since previous works ruled out shut-off implication of DprA in *Streptococcus thermophilus* [36] and highlighted the absence of Prx and XprA homologs in salivarius streptococci [31,34], this paves the way to uncover a shared streptococcal inhibitor. In the present study, we questioned the ComRS regulatory cascade by developing a deterministic mathematical model describing competence in *S*. *salivarius*. Confronting model predictions with experimental data suggested the presence of a pheromone inhibitor under the control of ComX, which was experimentally identified as PepF, a widely-distributed oligoendopeptidase in streptococci. We confirmed its role in competence shut-off through XIP pheromone degradation and pinpointed key peptide residues involved in its specific processing. Those results outline the benefits of combining computational and experimental methodologies to decipher the first proper shut-off effector in ComRS-containing streptococci.

## Results

### Mathematical simulations support pheromone degradation

**Fig 1:**
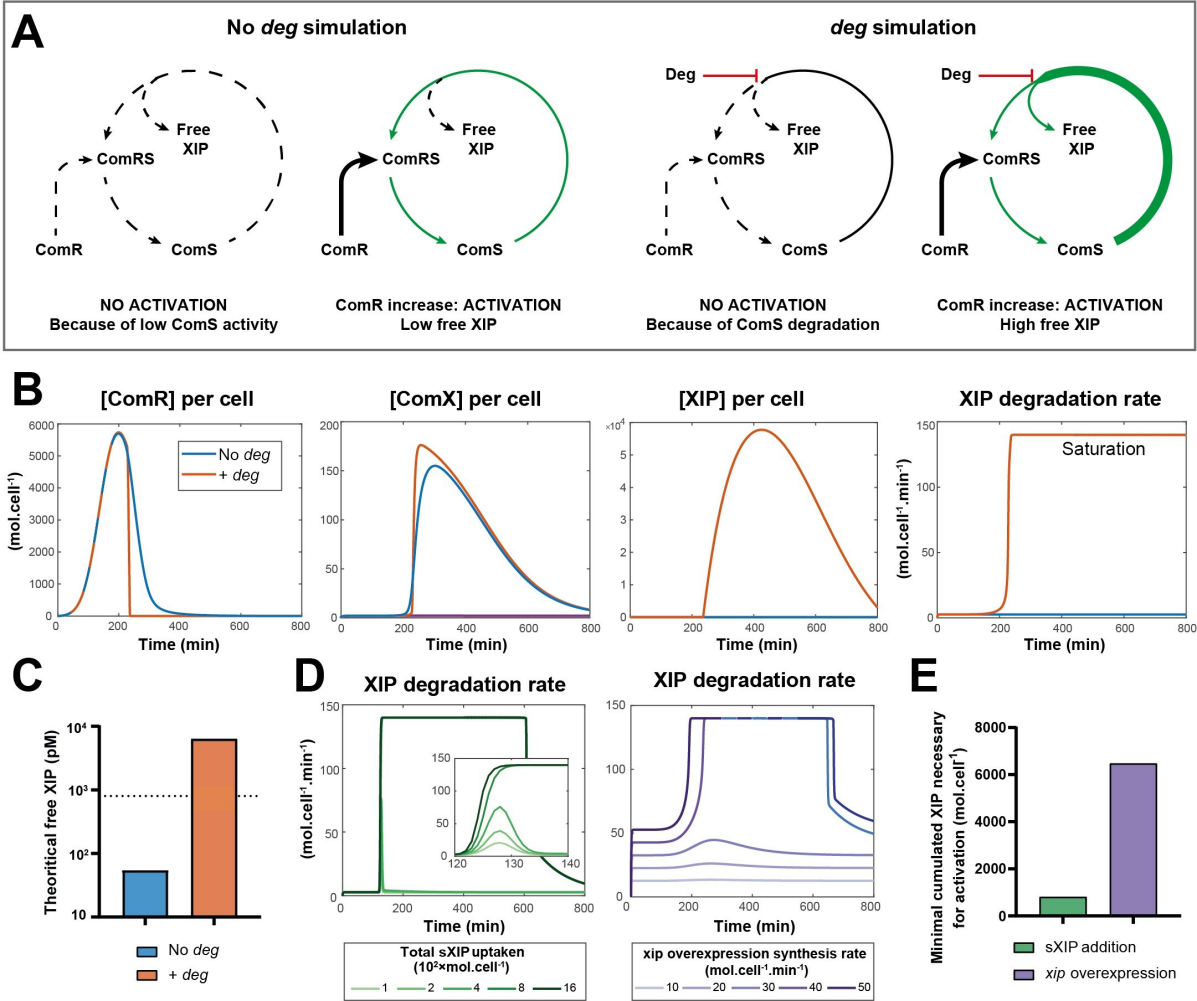
ComRS modelling suggests a peptidase activity. (A) Cartoons depicting the two types of simulations involving or not the *deg* player. (B) Simulated free ComR, ComX and ComS intracellular concentrations together with ComS degradation rate over time. Two different simulations output are depicted, one considering a degradation machinery (*deg*, blue) of ComS being fastly saturated and one without the *deg* player (No *deg*, orange). In both cases, competence was induced by increasing ComR concentration (8.5-fold), mimicking its genetic overexpression. (C) Theoritical maxima of free ComS (not included in the ComR•XIP complex) are computed for both simulations shown in panel A. Dotted line denotes the minimum sXIP extracellular concentration experimentally determined to trigger competence in *S*. *salivarius* [24]. (D) ComS degradation rate over time computed for the model including the *deg* variable in two types of competence inducing processes. First panel (green) simulates the extracellular addition of various sXIP concentrations and second panel (purple) simulates the genetic overexpression of *xip* at various levels. (E) Minimal quantity of XIP molecules to reach the treshold to activate competence in both scenarios modeled in panel D.

Previous investigations in *S*. *salivarius* HSISS4 [37] have highlighted the central role of ComR abundance in competence activation [7,36]. Strikingly, while pheromone overexpression could not trigger *comX* transcriptional activation, synthetic pheromone (sXIP) addition generated a strong and unimodal response in the population [24]. To puzzle out this intriguing observation, we investigated the ComRS regulatory cascade by designing a deterministic model based on previous *in silico* modeling of *S*. *thermophilus* and *S*. *mutans* [36,38]. While calibrating the model based on single-cell and luciferase data (see S1 Appendix for details), we realized that a low pheromone activity (i.e. low affinity for ComR or low cellular concentration) was required for running the model. Since high XIP-ComR affinity and high *comS* expression have been experimentally established [7,16,39], we hypothesized the presence of a negative player controlling the abundance of active ComS/XIP to explain this low pheromone activity. Various mechanisms could be at work such as sequestration, post-modification or degradation of XIP. However, we decided to simulate pheromone degradation as the most plausible mechanism since previously reported for similar regulatory systems in different streptococci [30,40,41]. We introduced this unknown inhibitor in our model (named *deg*), adjusted the free parameters, and compared the evolution of the cellular concentration of ComR, ComX and ComS with or without the *deg* variable (Fig 1A and 1B). In both cases, we simulated the ComR overproduction to high levels to trigger competence (up to 7,000 molecules per cell, according to a western-blot semi quantification previously performed [24]). As expected, free ComR intracellular concentration increases until a sharp drop corresponding to its conversion into the ComR**·**XIP complex (Fig 1B, 1^st^ panel). Concomitantly, ComX concentration starts to increase in both cases at 200 minutes to reach about 150 molecules per cell, which corresponds to the predicted number of ComX box in the HSISS4 genome [7] (Fig 1B, 2^nd^ panel). On the other hand, free ComS/XIP reaches high concentration (~6,000 molecules/cell) when simulated with the *deg* player, while only rising to 5 molecules/cell in its absence (Fig 1B, 3^rd^ panel). This counter-intuitive result is explained by the nature of the *deg* player simulated. Indeed, we modeled a ComS/XIP degradation machinery quickly saturated (Fig 1A and 1B [4^th^ panel]), which avoids spontaneous competence activation but allows high ComS production once overwhelmed. This resulted in a theoretical maximum free XIP concentration of 6 nM produced during competence (Fig 1C), which largely exceeds the experimentally determined sXIP threshold concentration (0.8 nM) required for activation [24]. Moreover, the introduction of the *deg* player generated a system that is only reactive at high ComR levels as previously shown with ComR-overproduction in single-cell experiments [24] (S1 Fig).

We next investigated the initial question regarding the differential sensitivity of the system to sXIP external addition vs genetically-encoded pheromone overexpression. To this aim, we simulated two different activations both including the *deg* player. In the first scenario, we simulated a high sXIP concentration (t = 120 min) internalized within 15 minutes by the cells. In a second scenario, we mimicked the *xip* overexpression by a constant production rate of XIP. We next ran those two simulations multiple times with a gradual increase of both XIP production parameters. For both induction systems, competence is only activated once the *deg* player is saturated (Fig 1D and S1 Appendix). Notably, the simulation of sXIP addition requires less XIP to trigger the system than the simulation with *xip* overexpression (Fig 1E), because a sharp increase in XIP concentration saturates the degradation player faster than a progressive production by the genetically-encoded peptide.

Altogether, those *in silico* results support the existence of a quickly saturated degradation machinery limiting XIP pheromone abundance.

### PepF negatively impacts competence activation

**Fig 2:**
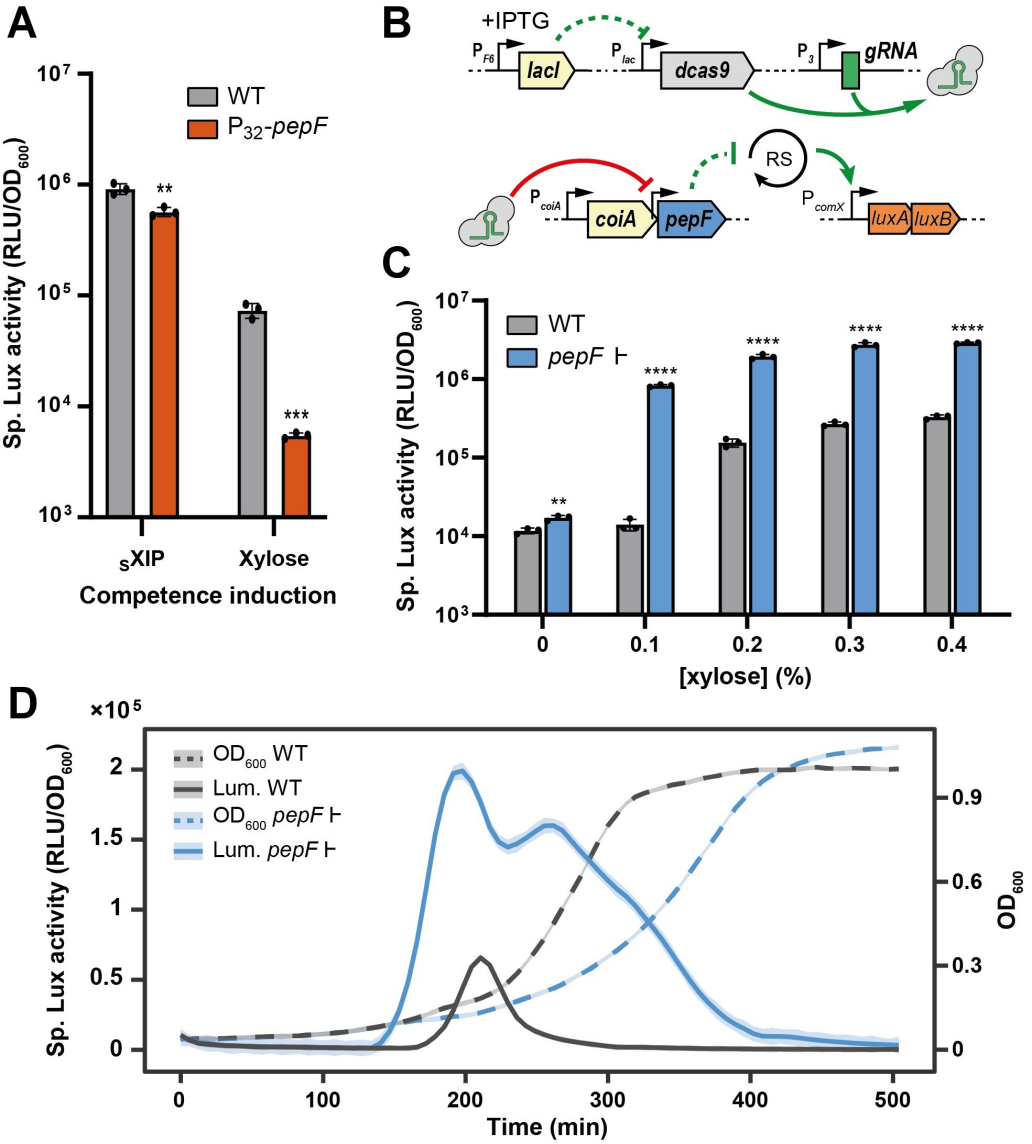
PepF down-regulates competence and constrains its activation to a limited time window. (A) Effect of *pepF* overexpression on P_*comX*_ activity. Data show specific luciferase activity (RLU/0D_600_) measured for WT (with P_*comX*_*-luxAB* as proxy) compared to a strain carrying a constitutive overexpression of *pepF* (*P*_32_*-pepF*). Competence was activated thanks to synthetic XIP addition (sXIP, 50 nM) or *comR* overexpression via a xylose-inducible promoter fused to *comR* (P_*xyl2*_*-comR*, 0.25% xylose). (B) Cartoon depicting the CRISPRi strategy used to repress *pepF* in panels C and D (RS: ComRS activation). (C) Effect of *pepF* inhibition on P_*comX*_ activity. Data show specific luciferase activity measured for WT (with P_*comX*_*-luxAB* as proxy) compared to a strain expressing a guide targeting the promoter of *pepF* (P_3_*-gRNA_9*). Both strains carry the IPTG-inducible dCas9 system (P_F6_*-lacI* P_*lac*_*-dcas9*) together with a xylose-inducible promoter fused to *comR* (P_*xyl2*_*-comR*). Competence was activated at different xylose concentrations (0, 0.1, 0.2, 0.3 and 0.4%) and the dCas9 repression system was activated at 100 μM of IPTG. Dots show biological triplicates, the bar shows the mean, and error bar denotes standard deviation. Statistical *t*-test was performed for each condition in comparison to the related control (WT) (**, *P* < 0.01; ***, *P* < 0.001; ****, *P* < 0.0001). (D) Growth (OD_600_) and kinetics of P_*comX*_ specific luciferase activity (Lum.) monitored over time with (*pepF* |-) or without *pepF* inhibition (WT). Data shown are the xylose 0.4% induction of panel C. Full lines and dashed lines are representative of the mean of biological triplicates, shaded lines represent standard deviation.

Since our model suggested the presence of a negative effector lowering pheromone activity, we aimed to search for such a player. Because recent studies showed post-translational pheromone control through peptidase activity in other streptococci [30,41] and a previous model advocated for the presence of a shut-off player under ComX control [36], we searched within the ComX regulon for putative peptidases. Using previous transcriptional data on the ComR/ComX regulons [7] together with the MEROPS database [42], we found that the genes coding for PepF and PepP were consistently activated by ComR, ComX and XIP addition (> 2.5 fold) [7]. While PepF (HSISS4_00369) is a Zinc oligoendopeptidase (M3 family), PepP (HSISS4_01648) is a metallo-exopeptidase (aminopeptidase or dipeptidyl-peptidase, M24 family) with unclear specificity [42]. Although not induced by competence activation, we also targeted the Zinc oligoendopeptidase PepO (HSISS4_01784) of the M13 family for its role in pheromone degradation in other streptococci [30,41] and the aminopeptidase PepQ (HSISS4_00546) as a functional homolog of PepP from the M24 family [42]. We succeeded to knock-out *pepP*, *pepO* and *pepQ* genes that showed no impact on competence activation (S2 Fig), but were unsuccessful to mutate *pepF*. Testing the involvement of *pepF* in competence shut-off the other way around, we overexpressed the peptidase gene with a strong constitutive promoter (P_32_*-pepF*) and monitored P_*comX*_ activation thanks to luciferase activity (P_*comX*_*-luxAB*). Competence was triggered either with a xylose-inducible promoter fused to *comR* (P_*xyl2*_*-comR*) or with sXIP addition (Fig 2A). For both competence-inducing conditions, we observed a decrease of P_*comX*_ activation in the *pepF* overexpressing strain, supporting previous theoretical assumptions.

As *pepF* overexpression inactivates competence, we expected PepF inhibition to result in higher competence activation. Since we were unsuccessful to knock out the gene, we designed a CRISPR-interference (CRISPRi) strategy with a guide targeting the upstream region of *pepF* (Fig 2B). Using the same reporter (P_*comX*_*-luxAB*) and competence-inducing (P_*xyl2*_*-comR*) systems as reported above, we monitored P_*comX*_ activation over time at different xylose concentrations (Fig 2C and 2D). Conversely to PepF overproduction, we observed higher competence induction in the PepF-depleted condition for each tested xylose concentration (Fig 2C). Those observations were confirmed by fine-tuning the level of *pepF* repression in the same strain with an IPTG gradient (P_*lac*_-*dcas9* under IPTG control), which displayed a gradual increase in intensity and timing (S3 Fig). We also observed a gradual growth inhibition corroborating our unsuccess to directly inactivate the *pepF* gene (Figs S3A and 2C). Notably, luciferase kinetics showed longer and earlier competence activation in the *pepF*-interfered condition, suggesting a dual role of the peptidase as a shut-off player and as a locking device preventing activation (Fig 2D). This latter feature was confirmed by spontaneous natural transformation upon *pepF* repression (transformation rate ~ 10^−5^). Altogether, these results underline the antagonist activity of PepF to limit both competence initiation and timing of activation.

### Transcription of *pepF* is competence-dependent

**Fig 3:**
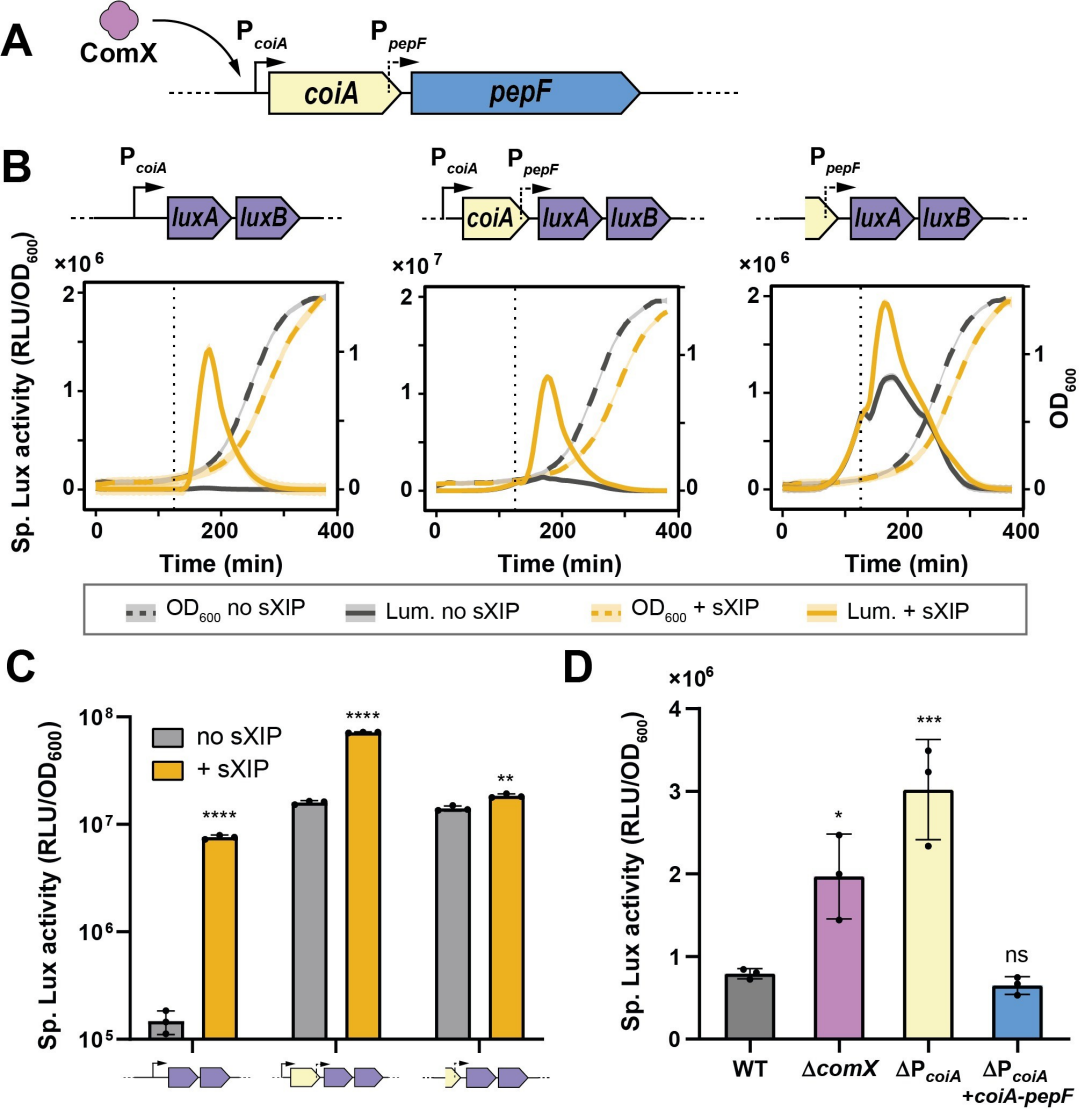
Transcriptional analysis of *pepF* and its ComX control. (A) Cartoon depicting the organization of the *coiA-pepF* operon. ComX controls P_*coiA*_ and a constitutive promoter of *pepF* was found in the coding sequence of *coiA*. (B) and (C) Kinetics (growth, OD_600_ and Lux activity, Lum.) (panel B) and mean specific luciferase activity (RLU/OD_600_) (panel C) reporting the transcription of the *coiA-pepF* operon upon competence activation. Lux activity was monitored with P_*coiA*_*-luxAB*, P_*coiA*_*-coiA-*P_*pepF*_*-luxAB*, and P_*pepF*_*-luxAB* luciferase reporter systems. The three constructs were incubated with (+ sXIP) or without (no sXIP) sXIP at a final concentration of 500 nM. sXIP was added after 120 min of growth (dotted lines in panel B) (D) Impact on competence activation of the ComX-control unplug on the expression of the *coiA-pepF* operon. Data show specific luciferase activity measured with a P_*comX*_*-luxAB* reporter strain (WT) and derivatives with *comX* deletion (Δ*comX*), P_*coiA*_ deletion (ΔP_*coiA*_), and P_*coiA*_ deletion complemented with an ectopic copy of a P_*coiA*_*-coiA-pepF* operon (ΔP_*coiA*_ + *coiA-pepF*). Competence was activated thanks to a xylose-inducible promoter fused to *comR* (P_*xyl2*_*-comR*, 1% xylose). In panel B, full lines and dashed lines are representative of the mean of biological triplicates, shaded lines depict standard deviation. In panels C and D, dots show biological triplicates, the bar shows the mean, and error bar the standard deviation. Statistical *t*-tests (panel C) or one-way ANOVA with Dunett’s test (panel D) were performed for each condition in comparison to the related mock to generate *P* values (*, *P* < 0.05; **, *P* < 0.01; ***, *P* < 0.001, ****, *P* < 0.0001; ns, non-significative).

Since a true shut-off mechanism implies its inception when the system turns on, we examined *pepF* expression during competence activation. A glimpse on its genomic context showed that *pepF* is located downstream of *coiA* (Fig 3A), a widely conserved late competence gene yet with unclear function [43]. Previous transcriptional data indicated upregulation of this bicistronic operon upon competence activation [7]. We confirmed those results using a P_*coiA*_*-luxAB* luciferase reporter system inserted at an ectopic locus (Fig 3B and 3C), showing P_*coiA*_ activation upon sXIP addition. To monitor more accurately *pepF* expression, we designed a new construct where we fused the whole *coiA* gene to the luciferase reporter genes (P_*coiA*_*-coiA-P*_*pepF*_*-luxAB*). As expected, we recorded an up-regulation upon competence activation via sXIP addition, presumably through P_*coiA*_ activation (Fig 3B and 3C). However, in contrast to the direct P_*coiA*_ fusion, this construct displayed a higher basal luciferase activity. Suspecting an additional *pepF* promoter within the *coiA* gene, we built a direct reporter fusion with the 3’ end of the *coiA* coding sequence (P_*pepF*_-*luxAB*). Luciferase monitoring revealed a high basal transcriptional activity of this region, slightly influenced by competence activation (Fig 3B and 3C). Together, these results suggest that *pepF* has a constitutive level of expression that is increased when competence is activated through the specific activation of P_*coiA*_.

To confirm the role of PepF as a shut-off effector under ComX control, we unplugged *pepF* from competence activation through the inactivation of *comX* (Δ*comX*) or the deletion of the promoter of *coiA* (ΔP_*coiA*_). Triggering competence thanks to ComR overproduction and monitoring activation with a P_*comX*_*-luxAB* fusion, we observed higher signals for the two ComX-unwired constructs (Fig 3D). Supporting those results, a ΔP_*coiA*_ complementation with an ectopic insertion of the *coiA-pepF* operon (ΔP_*coiA*_ + *coiA-pepF*) restored the signal observed in the wild-type background (Fig 3D).

Altogether, those results reveal that PepF has a dual impact on competence regulation. While its control through ComX triggers competence shut-off, its basal production through the constitutive promoter in the *coiA* coding sequence could prevent competence initiation in unfavorable conditions, acting as an additional locking device.

### PepF degrades XIP *in vitro*

**Fig 4:**
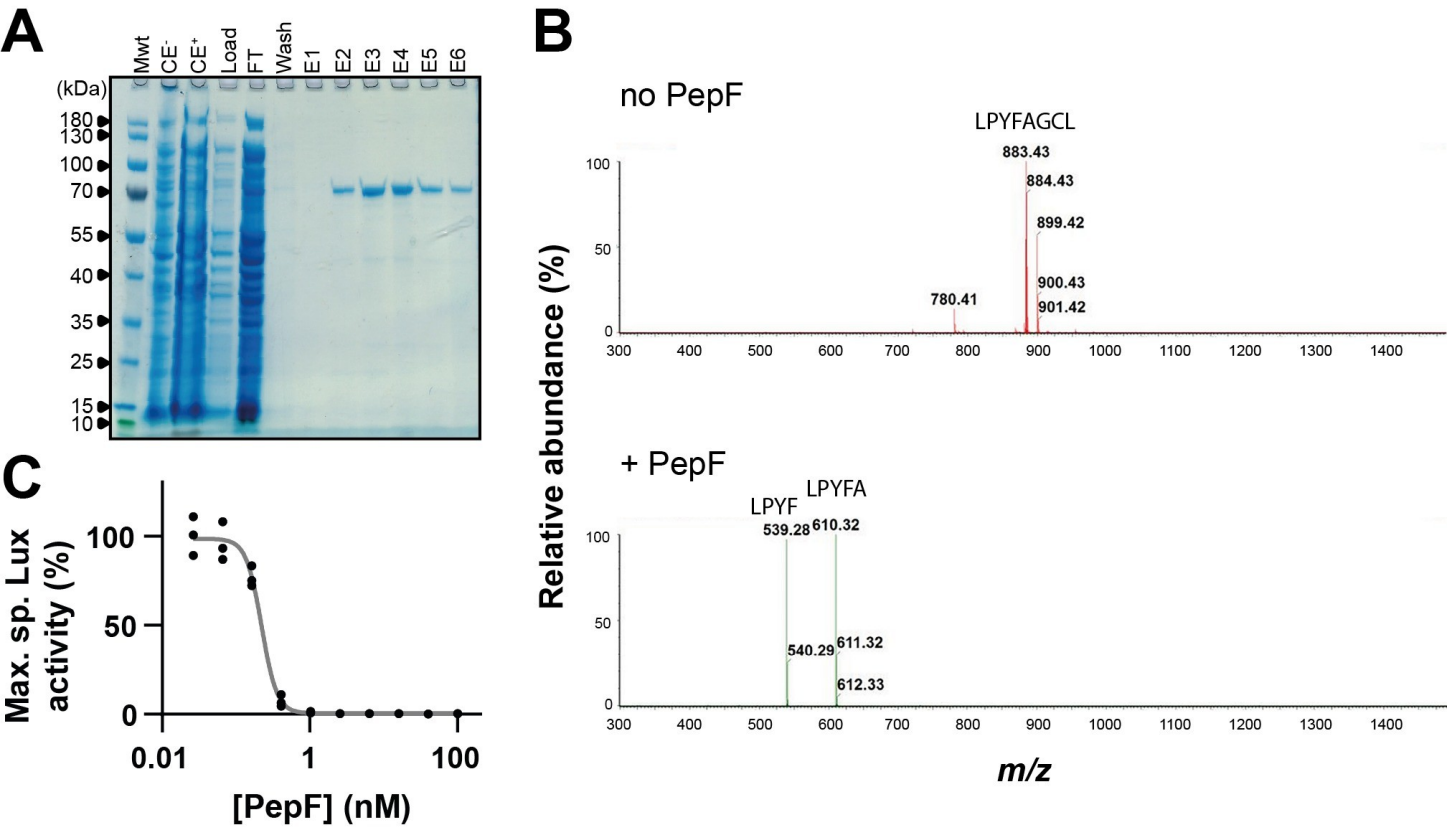
PepF degrades XIP *in vitro*. (A) SDS-PAGE of PepF purification (Mwt: molecular weight, CE: crude extract with (+) or without arabinose induction (-), FT: flow-through, E1-6: elution fractions 1 to 6.) (B) LC-MS chromatogram of sXIP incubated without (no PepF, red) or with PepF (+ PepF, green). sXIP (500 nM) was incubated at 37°C for 4 h with PepF (100 nM). The *m/z* value of 883.43 ([M+H]^+^) corresponds to the unprocessed peptide LPYFAGCL. The *m/z* values of 539.28 ([M+H]^+^) and 610.32 ([M+H]^+^) correspond to fragments LPYF and LPYFA, respectively. (C) Maximum luciferase activity (RLU/OD_600_) of a competence reporter strain defective for the genetically-encoded pheromone XIP (P_*comS*_*-luxAB* Δ*comS*). sXIP (500 nM) was incubated at 37°C for 4 h with increasing concentrations of PepF (0, 0.025, 0.065, 0.16, 0.4, 1, 2.56, 6.4, 16, 40, and 100 nM). The reaction mixture was then 10-fold diluted by addition to an exponential growing culture (CDM) of the reporter strain. Maximum specific luciferase activity is displayed as the percentage of signal in comparison to a control culture with an addition of sXIP without PepF digestion. Dots represent technical replicates and the curve is a non-linear fit of inhibition. EC_50_ = 0.22 ± 0.01 nM (standard error).

To get insights into the molecular mechanism of the PepF-mediated competence inhibition, we purified PepF fused to a StrepTag (Fig 4A) and incubated it with sXIP. We next analyzed the peptide integrity thanks to mass spectrometry. While incubation of the peptide without the peptidase generated a major peak (*m/z* value of 883.43, [M+H]^+^) corresponding to the intact sXIP peptide (LPYFAGCL), incubation with PepF resulted in the loss of the full-length peptide and the appearance of two main peaks (*m/z* values of 539.28 and 610.32, [M+H]^+^), corresponding to LPYF and LPYFA fragments, respectively (Fig 4B).

We then sought to assess the efficiency of the peptidase to degrade the pheromone. We used an indirect pheromone quantification method [41] by monitoring luminescence from a P_*comS*_*-luxAB* reporter strain defective for the genome-encoded XIP (Δ*comS*). By incubating sXIP at a constant concentration (500 nM) with a gradient of PepF concentration, we measured an enzyme concentration affording half luminescence (EC_50_) of ~ 0.2 nM, corresponding to a ~2,500-fold pheromone-peptidase ratio or to an apparent turnover rate of ~ 0.2 sec^-1^ (Fig 4C). This measurement falls within the range reported for other streptococcal oligoendopeptidases tested with a similar assay (~ 30 to ~ 0.01 sec^-1^) [30,41]. Together, those data suggest that PepF competence inhibition acts through XIP degradation.

### XIP positions 4 and 5 are crucial for PepF activity *in vitro*

**Fig 5:**
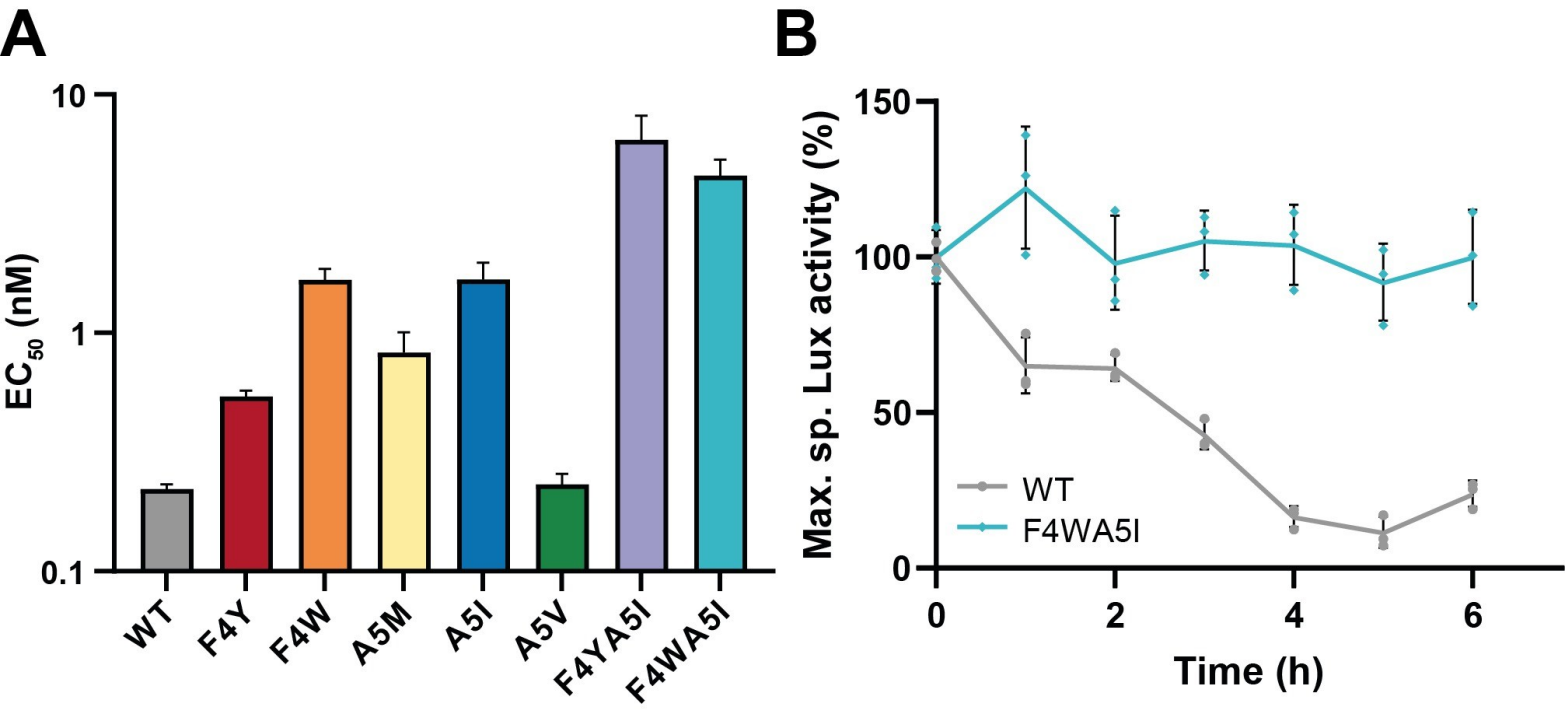
Degradation of PepF-resistant sXIP peptides. (A) PepF degradation efficiency for XIP (LPY[F/Y/W][A/M/I/V]GCL) variants. PepF was incubated 4 hours at 37°C at various concentration with XIP variants (500 nM) and EC_50_ was determined (S4 Fig). Bars denote standard deviations on the EC_50_ computation. (B) XIP and XIP_F4WA5I_ kinetics of degradation by PepF. PepF (8 nM) and the peptides (500 nM) were incubated 6 hours at 37°C and sampled every hour for analysis. Reaction was stopped by adding 1 mM EDTA and further added to the P_*comS*_*-luxAB* Δ*comS* reporter strain as previously described. Dots show technical triplicates, the line connects mean values with error bars (standard deviation).

We next investigated which residues of the pheromone are crucial for peptidase cleavage and screened 20 peptides variants with single XIP mutations. We incubated those peptides with PepF at a mid-range concentration previously identified for the native XIP ([PepF] of 7 nM and [XIP] of 500 nM). Next, we added the peptides incubated or not with PepF to the P_*comS*_*-luxAB* Δ*comS* reporter strain and analyzed the percentage of signal loss upon incubation with the peptidase. Comparing the signal loss of the peptide variants at the maximum luciferase activity, we identified 4 mutations (F4Y, F4W, A5I, A5M) probably resulting in a higher peptidase resistance (S4 Fig). Notably, those mutations were located at XIP positions 4 and 5, both at position -1 (P1) regarding the two cleavage sites reported above. To quantify more accurately the PepF resistance of variants at those two positions, we used the same degradation assay as used for native sXIP and measured the EC_50_ for each variant (Figs 5A and S5). Since F4W, F4Y and A5I variants were the most resistant, we combined mutations at positions 4 and 5 in a single peptide to increase its robustness to degradation. The F4W-A5I and F4Y-A5I variants exhibited slower proteolysis *in vitro* (Fig 5A) and the F4W-A5I variant showed a reduced degradation kinetics compared to the WT pheromone (Fig 5B and 5C). Because our degradation assay is based on an indirect method of quantification through the use of a reporter strain, bias could be introduced by the effect of the mutations on the ComR-pheromone affinity. To rule out this possibility, we measured the ComR-binding properties of the peptides by electrophoretic mobility shift assays (S6 Fig). Since no correlation between peptide-binding to ComR and pheromone sensitivity to degradation could be drawn, we concluded that the luciferase degradation assay reflects the peptide susceptibility to PepF.

Together, those results show that residues at XIP positions 4 and 5 play a key role for PepF degradation *in vitro*.

### PepF-resistant XIP variants result in higher and longer competence activation

**Fig 6:**
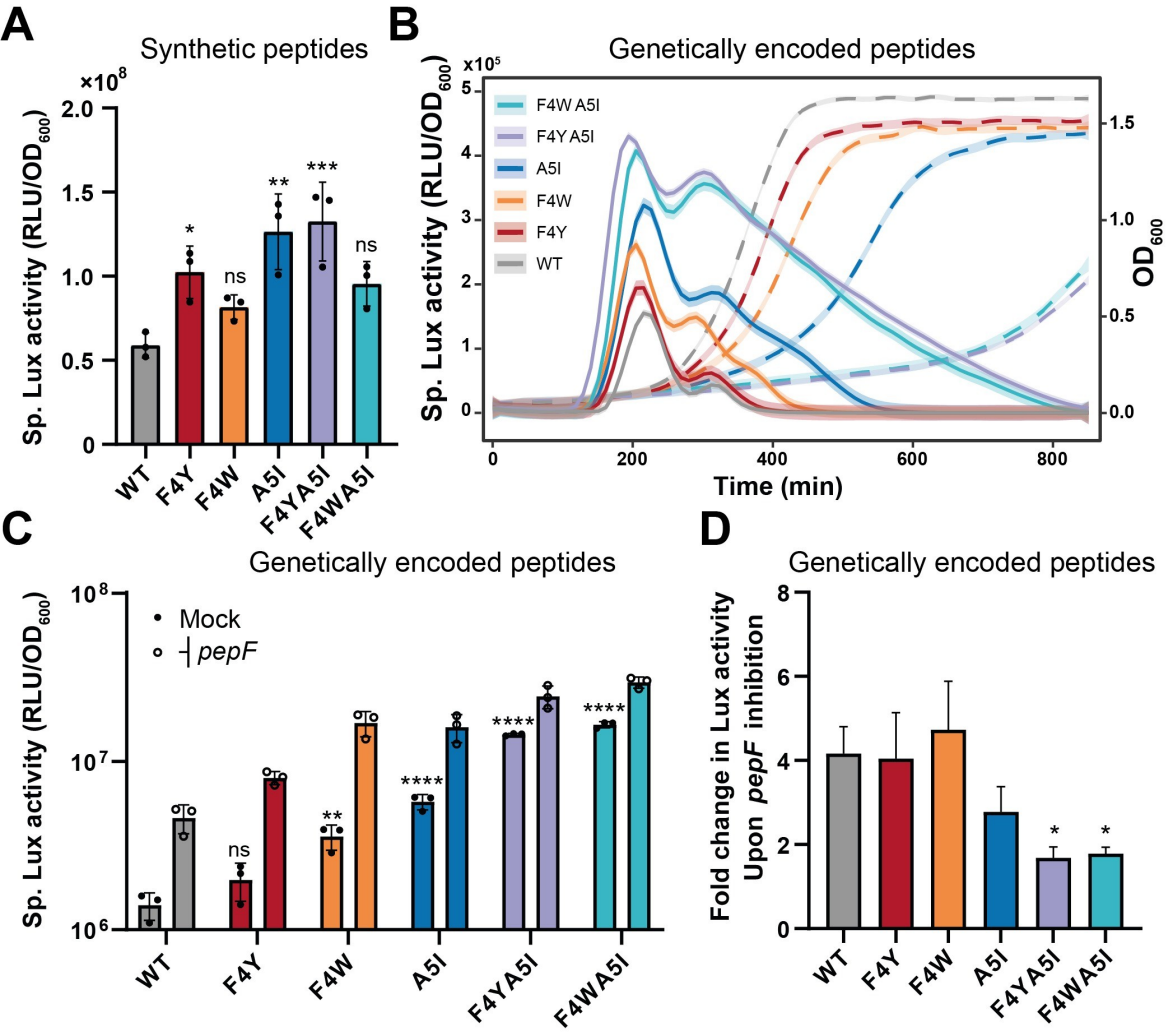
PepF-resistant peptides escape shut-off *in vivo*. (A) Impact on competence activation of the addition of the synthetic peptide variants. Data show specific luciferase activity (RLU/OD_600_) measured with a P_*comS*_*-luxAB* reporter strain defective for the pheromone (Δ*comS*) supplemented with the synthetic peptide variants (5 nM). (B) and (C) Kinetics (OD_600_, dashed line; Sp. Lux activity, solid line) (panel B) and mean specific luciferase (panel C) of competence activation by genetically-encoded peptide variants (ComS, 24 aa). The *comS* native gene was mutated to produce the XIP variants into a xylose-inducible *comR* (P_*xyl2*_*-comR*) P_*comX*_*-luxAB* reporter background together with the *pepF* dCas9 inhibition module. The *comR* native gene was deleted to avoid any toxic spontaneous competence activation. Competence was induced by adding 0.5% of xylose. The bar represents the mean of biological triplicates (plain dots for competence activation and empty dots for competence activation together with *pepF* inhibition). (D) Fold change in specific luciferase activity for competence activation upon *pepF* inhibition for each peptide variant based on results presented in panel C. Bars denote standard deviations on specific luciferase activity or fold change computation. Dots denote biological replicates. In panel B, solid lines and dashed lines are representative of the mean of biological triplicates of specific luciferase activity and growth, respectively. Shaded lines depict standard deviation. One-way ANOVA with Dunett’s test was performed for each condition in comparison to WT (WT without *pepF* inhibition for panel C; *, *P* < 0.05; **, P<0.01; ***, P<0.001; ****, *P* < 0.0001).

To confirm *in vivo* the relevance of *in vitro* data, we first measured the activation of a competence reporter strain defective for the pheromone (P_*comS*_*-luxAB* Δ*comS*) with the addition of synthetic peptides. To avoid a saturation of PepF with synthetic peptides that would mask the effect of the mutations, we supplemented the culture of the reporter strain with very low concentrations (5 nM) of pheromone variants (Figs 6A and S7). For most of the peptides tested, we observed a higher and longer activation. Strikingly, the most resistant peptides *in vitro* did not result in the best activators, presumably because of the observed change in ComR binding (S6 Fig) or because the sudden sXIP increase saturates quickly PepF, as suggested by our mathematical simulations.

To overcome this issue and to evaluate the effect of those mutations in a more relevant physiological context, we next transferred the most resistant peptide variants to the native *comS* locus of *S*. *salivarius*. We introduced the variants as full-length precursors (24 aa) under P_*comS*_ control together with the upstream deletion of *comR* to avoid any toxic effect due to spontaneous competence activation [7]. The strain also carried a P_*comX*_*-luxAB* reporter fusion, a xylose-inducible *comR* system as competence activator, and a CRISPRi repression system targeting *pepF*. In a first set of experiments, we overexpressed *comR* and monitored competence activation with the different XIP variants (WT, F4Y, F4W, A5I, F4Y-A5I, F4W-A5I) (Fig 6B). Simple mutation at position 4 or 5 resulted in longer and higher competence activation but double mutations F4W-A5I and F4Y-A5I displayed dramatic effects on both growth and competence activation, resulting in a 7-hours growth delay and a ~10 and ~15-fold increase in competence activation, respectively (Fig 6B and 6C). Noteworthy, growth delays were only recorded when competence was activated. This suggests that the extension of the timing of competence generated by XIP mutations leads to a toxic phenotype. We next induced the CRISPRi system to repress PepF-mediated shut-off together with *comR* overexpression to activate competence. We assumed that for a PepF-resistant peptide, inhibiting *pepF* would not increase competence activation. We observed a lower increase of competence activation for F4W-A5I and F4Y-A5I peptides upon *pepF* inhibition (Fig 6C and 6D), suggesting that ultra-resistant pheromones can bypass the PepF-mediated shut-off.

Together, those results show that PepF-resistant XIP variants escape the shut-off mechanism, which drastically alter the kinetics and the strength of competence activation in *S*. *salivarius*.

## Discussion

**Fig 7:**
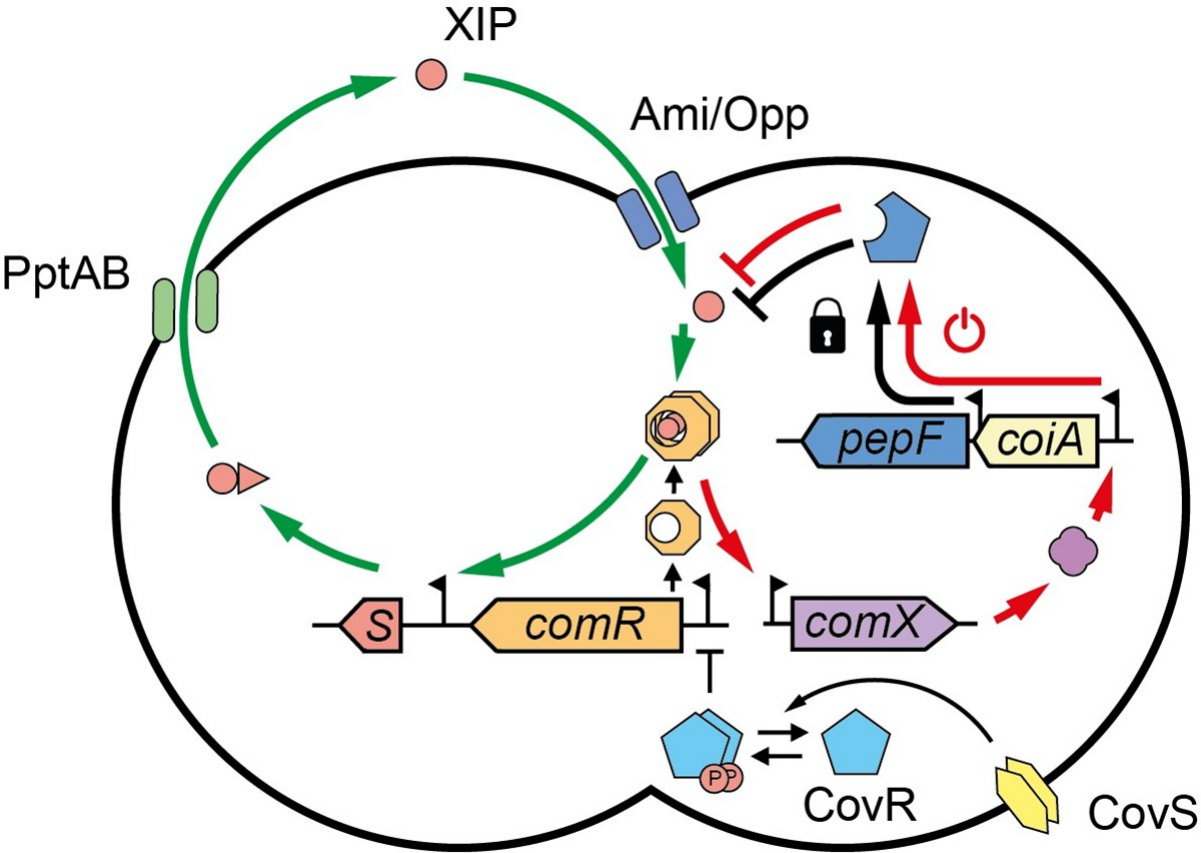
Model of competence regulation integrating the PepF-mediated shut-off in *S*. *salivarius*. Upon CovRS repression release, ComR reaches a threshold concentration allowing the activation of a positive feedback loop (green arrows). The positive loop is triggered by XIP binding to ComR, producing the ComR•XIP complex which activate *comS* transcription. ComS is then exported by the transporter PptAB and maturated. The mature XIP pheromone can then enter the cell by the oligopeptide generic transporter Ami/Opp and bind ComR to enhance the loop. This activation can take place if XIP levels are sufficient to saturate the activity of PepF at native expression (locker icon). In parallel, the ComR•XIP complex will trigger the transcription of *comX*, encoding the central regulator of competence. This will activate all the late genes necessary for natural transformation including the *coiA-pepF* operon. PepF accumulation will result in XIP degradation, generating a negative feedback-loop (red arrows, power-off icon) on the ComRS system, ultimately leading to competence exit.

Although competence activation has been extensively studied in streptococci, authentic exit mechanisms from ComRS initiation remained challenging to identify. Investigations presented in this work show that PepF, an essential oligoendopeptidase in *S*. *salivarius*, is responsible for the shut-off of the ComRS pathway through ComX activation. This finding bridges the positive feedback loop on the production of the ComS pheromone with a negative feedback loop acting on ComS/XIP degradation. This regulation based on opposite loops sets up a new model (Fig 7) explaining the excitability of the system and the limited time window of competence activation in *S*. *salivarius*.

One striking feature of this novel ComRS antagonist player is its essentiality in *S*. *salivarius*. Although PepF inactivation in *Lactococcus lactis* results in a slight growth defect only in chemically-defined medium [44] and has no major impact in *Bacillus subtilis* [45], we were unable to produce a knock-out of the peptidase (S2 Fig) and observed a strong growth defect upon *pepF* transcriptional inhibition (Figs 2D and S3). Remarkably, we could uncouple *pepF* from the negative feedback loop by knocking out the promoter of *coiA* controlled by ComX (Fig 3D), which suggests that essentiality is associated to its constitutive production rather than its role in competence shut-off. Since *pepF* inhibition remains toxic in competence-deficient strains (Δ*comX* and Δ*comRS*; S8 Fig), *pepF* essentiality in *S*. *salivarius* originates most probably from another function than competence regulation. In *L*. *lactis*, PepF was shown to play a major role in signal peptide recycling [46], a function also described for the homolog of PepF (OpdA) in *Salmonella enterica* [47]. In addition, OpdA of *Escherichia coli* was shown to be linked to cell-cycle regulation [48]. Those previous results suggest that *S*. *salivarius* PepF may have multiple functions in addition to competence regulation that could explain its essentiality.

As previously mentioned, PepF is a metallo-endopeptidase from family M3 containing a classical Zinc-binding motif HEXXH [49], which is involved in catalysis of peptide cleavage [50]. Regarding the crystal structures of the M3 family, two variations in the global fold could be found. While the bacterial Pz-peptidase A from *Geobacillus collagenovorans* is globular and displays an inner closed channel where the peptide docks, the eukaryotic Thimet oligopeptidase resembles to an open bivalve with two distinct domains [50]. Unfortunately, no structural data are available to know which is the typical M3 structure displayed by PepF in order to understand its peptide specificity. Yet, the analysis of hydrolyzed substrates by PepF homologs from *Streptococcus agalactiae* and *L. lactis* (72% and 64% of identity, respectively) provides interesting data on the selectivity of peptide length and cleavage sites [49,51]. Indeed, the peptidase shows a wide specificity with size-limitation from 7 to 17 amino-acids and with a high prevalence for hydrophobic residues at the P1 site (position -1 regarding the cleavage site). Although double peptide cleavage was reported, phenylalanine was observed at P1 in many cases but no alanine until now [49,51]. Those results can be compared to our data showing two cleavage sites, the first one after the fourth residue (phenylalanine) and the second after the fifth residue (alanine). Interestingly, among the 20 XIP variants maintaining competence activation properties that were screened for PepF resistance, the most resistant pheromones exhibited mutations at P1 regarding the two cleavage sites. For single substitutions, *in vitro* degradation data (Fig 5A) showed that F4W and A5I variants generated the most resistant peptides. Since tryptophan and isoleucine are the bulkiest residues tested at these positions, it is likely that a higher steric effect of those residues at P1 interferes with peptide access to the catalytic site or its positioning regarding catalytic residues. Besides PepF, we also show that the exopeptidase PepP of the ComR/ComX regulon does not seem to be involved in the control of competence. The *pepP* gene is directly induced by the ComRS system (~11 fold) and slightly up-regulated by ComX (~3 fold) [7]. Since *pepP* is included in a bacteriocin gene cluster (*slvW-ComEB*), its functional role could be related to bacteriocin processing and/or immunity. Alternatively, PepP exopeptidase activity may be required for a further processing of XIP after its cleavage by PepF.

In this work, we have uncovered a true shut-off mechanism in *S*. *salivarius* involving the degradation of XIP through a negative feedback loop and restricting competence to a limited time window. Since we were unable to completely abolish competence exit by inhibiting PepF (Fig 2D) or using XIP mutations (Fig 6B), we can hypothesize that one or more additional player(s) allow the exit from competence in *S*. *salivarius* such as found in *S*. *pneumoniae* where at least two shut-off mechanisms were reported [26,27,29]. Nevertheless, one could question if the PepF-mediated mechanism can be expanded to other species. In the salivarius group, the XIP sequence of *S*. *salivarius* HSISS4 (LPYFAGCL) is identical in *S*. *thermophilus*, suggesting that PepF can also limit the pheromone availability in this species. Yet, the third member of the salivarius group, *S*. *vestibularis*, displays another XIP sequence (XIP_Sve_, VPFFMIYY). Interestingly, PepF from HSISS4 that is sharing high identity (98.5%) with *S*. *vestibularis* PepF (S9A Fig) is also able to degrade XIP_Sve_ (S9B Fig). However, PepF-mediated XIP_Sve_ degradation is ~15-fold less efficient compared to XIP_Ssa/Sth_. Since XIP_Sve_ displays lower affinity (~10 fold) for its cognate ComR [39], it is tempting to speculate that the pheromone sequence has evolved differently in those species to maintain a balance between peptidase susceptibility and ComR affinity. Since PepF is able to recognize a broad spectrum of peptides [49,51], we can ask whether this peptidase plays a role in other ComRS-containing species for competence shut-off and initiation. Although streptococci display a high XIP pheromone diversity (for a review, see [10]), PepF in other ComRS-containing streptococci could have different specificities, more adapted to recognize their native XIP peptides. Moreover, high synteny conservation between *coiA* and *pepF* in *Firmicutes* (S10 Fig) suggests a key-maintained role of PepF in competence control. Interestingly, PepF was previously shown to control peptide signaling for competence and sporulation in *B*. *subtilis* [45].

This study sheds light on the first true competence shut-off mechanism in ComRS-containing streptococci, which is mediated by the activation of a peptidase embedded in a negative feedback loop. Since quorum-sensing based on peptide pheromones is a widely spread system in Gram-positive bacteria, it is likely that this proteolytic mechanism can be broadened to many other communication devices.

## Materials and methods

### Mathematical modeling

A deterministic model was built using ODEs (Ordinary Differential Equations) with the MATLAB software and its ode23 solver. The mathematical formulation of the ComRS system was mainly built on two pre-existing models [36,38]. In short, we used luciferase data (P_*comR*_*-luxAB*, P_*comX*_*-luxAB* and P_*comS*_*-luxAB*) to calibrate the model and find parameters values as previously described [36]. We then validated the model using single-cell microscopic data together with semi-quantitative immunoblotting of ComR (P_*comX*_*-gfp*^*+*^ P_*xyl2*_*-comR*, see S1 Appendix and [24]). Details on the modeling methodology, the MATLAB code, and specific experimental data used for model calibration and validation can be found in S1 and S2 Appendices, and S1 Dataset, respectively.

### Bacterial strains, plasmids, and oligonucleotides

Bacterial strains, plasmids, and oligonucleotides used in this study are listed and described in supplementary information (S1–S4 Tables).

### Growth conditions

*S*. *salivarius* HSISS4 [52] and derivatives were grown at 37°C without shaking in M17 (Difco Laboratories, Detroit, MI) or in chemically-defined medium (CDM) [53] supplemented with 1% (w/v) glucose (M17G, CDMG, respectively). *E*. *coli* TOP10 (Invitrogen) was cultivated with shaking at 37°C in LB (Lysogeny Broth). Chromosomal genetic constructions were introduced in *S*. *salivarius* via natural transformation [54]. Electrotransformation of *E*. *coli* was performed as previously described [55]. We added D-xylose (0.1 to 1%; w/v), IPTG (1 mM), ampicillin (250 μg/mL), spectinomycin (200 μg/mL), chloramphenicol (5 μg/mL) or erythromycin (10 μg/mL), as required. The synthetic peptides (purity of 95%), were supplied by Peptide 2.0 Inc. (Chantilly, VA) and resuspended first in dimethyl-formamide (DMF) and diluted in water to reach low DMF concentration (final of 0.02%). Solid plates inoculated with streptococci cells were incubated anaerobically (BBL GasPak systems, Becton Dickinson, Franklin lakes, NJ) at 37°C.

### Competence induction and construction of mutants

To induce competence, overnight CDMG precultures were diluted at a final OD_600_ of 0.05 in 500 μL of fresh CDMG and incubated 100 min at 37°C. Then, the pheromone sXIP and DNA (Gibson assembled PCR products or plasmids) were added and cells were incubated for 3 h at 37°C before plating on M17G agar supplemented with antibiotics when required. Null mutants were constructed by exchanging (double homologous recombination) the coding sequences (CDS) of target genes (sequence between start and stop codons) for chloramphenicol, erythromycin or spectinomycin resistance cassette. If stated, mutants were cleaned for the *lox* site-flanked resistance cassette as previously described [54]. Integration of the antibiotic resistance cassette at the right location was subsequently checked by PCR and sequencing.

Genetic *comR* or *pepF* overexpression were designed by ATG fusion to a mild xylose-inducible promoter (P_*xyl2*_, associated to the *xylR* regulator gene) or to a strong 5’-UTR optimized constitutive promoter (P_32_-*opt*), respectively. The constructs were associated to a chloramphenicol resistance cassette and integrated at the permissive tRNA serine locus (*HSISS4_r00062*) for P_*xyl2*_-*comR* or at a second permissive locus (*tnpII*, downstream of *HSISS4_01854*) for P_32_*-pepF*. Luciferase reporter systems were constructed by fusing promoters to the *luxAB* reporter genes and inserted with a spectinomycin resistance cassette at the permissive tRNA threonine locus (*HSISS4_r00061*) or *tnpII* locus (downstream of *HSISS4_01854*).

For transcriptional inhibition of *pepF* by CRISPR-interference, a previously constructed strain harboring a codon-optimized version of *lacI* together with a Cas9 catalytic mutant (dCas9) under the control of a P_*lac*_ promoter with a P_*comX*_*-luxAB* reporter system was used [24,56]. We introduced in this strain a xylose-inducible *comR* module (P_*xyl2*_*-comR*) at the *suc* locus (upstream of *HSSS4_01641*). After excising the newly introduced resistance cassette by the Cre-*lox* system, a guide targeting the upstream region of *pepF* was introduced at the *gor* locus (downstream of *HSISS4_00325*) as previously described [24,56].

For genetically-encoded *comS* mutants, a scareless OroP system was used [57]. Briefly, a P_32_*-cat-oroP* cassette was introduced at the *comRS* locus and cassette insertion was selected on chloramphenicol. A second transformation was performed on the strain, introducing a *comS* modified construct with *comR* deletion. The transformants were selected on plates with 1 mg/mL 5-Fluoroorotic acid (5-FOA) and sequenced to validate the DNA insertion. The same strategy was used to generate the *comS-*defective mutant used as a reporter for sXIP-PepF degradation assays.

For ΔP_*coiA*_ complementation, the complete *PcoiA-coiA-pepF* operon was amplified from WT and introduced at an ectopic neutral locus (*fba*, upstream of *HSISS4_01793*). Finally, to construct the P_*comS*_*-opt-gfp*^*+*^ strain used for model validation, a 5’-UTR optimized version of P_*comS*_ was order as a Gblock (IDT) and introduces at the *tRNA*_*thr*_ locus with a spectinomycin resistance cassette.

### Luciferase activity

Overnight precultures were diluted at a final OD_600_ of 0.05. A volume of 300 μL of culture samples was transferred in the wells of a sterile covered white microplate with a transparent bottom (Greiner, Alphen a/d Rijn, The Netherlands). These culture samples were supplemented with D-xylose or IPTG if stated or further incubated for 100 min at 37°C before supplementation with sXIP or other peptides variants. Growth (OD_600_) and luciferase (Lux) activity (expressed in relative light units, RLU) were monitored at 10 min intervals during 6 to 24 h in a Hidex Sense microplate reader (Hidex, Lemminkäisenkatu, Finland). Specific or maximum specific Lux activity were obtained by dividing Lux activity by the OD_600_ for each measurement and summing all the data obtained over time or selecting the maximum value, respectively. Biological or technical triplicates were then averaged. Statistical analyses of simple and multiple comparisons to the control mean were performed with *t*-test (unilateral distribution, heteroscedastic) and one-way ANOVA with Dunnett’s test, respectively. For both, standard deviations and *P* values were calculated.

### Protein purification

For PepF purification, the PCR-amplified *pepF* gene from strain HSISS4 was cloned into the pBAD-covR-ST_N-ter_ vector [24]. Because the catalytic region of PepF is predicted at the C-terminus, the StrepTag was placed at the N-terminus by Gibson assembly, electroporated into *E*. *coli* Top10 as previously described [55] and the final construct was verified by DNA sequencing. Purification of proteins was performed as described previously with some modifications [39,58]. One liter of culture at an initial OD_600_ of 0.05 was incubated at 42°C with shaking until an OD_600_ of 0.5. Protein production was induced by adding 0.05% of L-arabinose and the culture was incubated at 30°C for 4 h with continuous shaking. Cells were then harvested by centrifugation (5,000 × *g* for 15 min), and pellets were resuspended in cold buffer W (100 mM Tris-HCl, pH 8.0, 150 mM NaCl, without EDTA) supplemented with 0.5 mg/mL of lysozyme, incubated for 30 min at 4°C, and sonicated at 4°C. The soluble fractions were collected after centrifugation (15,000 × *g* for 60 min at 4°C) and purification of the recombinant proteins was performed by one-step affinity chromatography with Strep-Tactin Superflow column (IBA BioTAGnology, Göttingen, Germany) according to the manufacturer’s instructions. The same protocol was used to purify ComR with a previously constructed Top10 strain carrying a pBAD-comR-ST_C-ter_ plasmid [7]. Purified proteins were aliquoted and stored at −80°C in buffer W with 10% glycerol [v/v]. Protein purity was analyzed by SDS-PAGE and concentration was measured using a Nanodrop apparatus (A280 method; ThermoFisher Scientific).

### Peptide separation using nanoUPLC

sXIP was incubated with or without recombinant PepF at 37°C for 0 or 4 h in reaction buffer (20 mM Tris HCl, pH 7.0) at a final concentration of 100 nM and 500 nM, respectively. 90 μL of sample were acidified by adding 10 μL of 0.1% (v/v) TFA. Samples were desalted and concentrated by elution with 20 μL of 50% acetonitrile, 0.1% formic acid using C18 ZipTip (Millipore) according to manufacturer’s instructions. C18 ZipTip was also used to remove PepF (69 kDa). Peptide mixture was separated by reverse phase chromatography on a NanoACQUITY UPLC MClass system (Waters) working with MassLynx V4.1 (Waters) software. 5 μL were injected on a trap C18, 100 Å 5 μm, 180 μm x 20 mm column (Waters) and desalted using isocratic conditions with a flow rate of 15 μL/min using a 99% formic acid and 1% (v/v) ACN buffer for 3 min. Peptide mixture was subjected to reverse phase chromatography on a C18, 100Å 1.8 μm, 75 μm x 150 mm column (Waters) PepMap for 130 min at 35°C at a flow rate of 300 nL/min using a two part linear gradient from 1% (v/v) ACN, 0.1% formic acid to 35% (v/v) ACN, 0.1% formic acid for 90 min and from 35% (v/v) ACN, 0.1% formic acid to 85% (v/v) ACN, 0.1% formic acid for 10 min. The column was re-equilibrated at initial conditions after washing 30 min at 85% (v/v) ACN, 0.1% formic acid at a flow rate of 300 nL/min. For online LC-MS analysis, the nanoUPLC was coupled to the mass spectrometer through a nano-electrospray ionization (nanoESI) source emitter.

### LC-QTOF-MS/MS analysis (DDA)

DDAs (Data Dependent Analysis) were performed on an SYNAPT G2-Si high definition mass spectrometer (Waters) equipped with a NanoLockSpray dual electrospray ion source (Waters). Precut fused silica PicoTip Emitters for nanoelectrospray, outer diameters: 360 μm; inner diameter: 20 μm; 10 μm tip; 2.5” length (Waters) were used for samples and Precut fused silica TicoTip Emitters for nanoelectrospray, outer diameters: 360 μm; inner diameter: 20 μm; 2.5” length (Waters) were used for the lock mass solution. The eluent was sprayed at a spray voltage of 2.8 kV with a sampling cone voltage of 25 V and a source offset of 30 V. The source temperature was set to 80°C. The cone gas flow was 20 L/h with a nano flow gas pressure of 0.4 bar and the purge gas was turned off. The SYNAPT G2Si instrument was operated in DDA (data-dependent mode), automatically switching between MS and MS2. Full scan MS and MS2 spectra (*m*/*z* 400–2000) were acquired from 2 min after injection to 30 min in resolution mode (20,000 resolution FWHM at *m*/*z* 400) with a scan time of 0.1 sec. Tandem mass spectra of up to 10 precursors were generated in the trapping region of the ion mobility cell by using a collision energy ramp from 17/19 V (low mass, start/end) to up to 65/75 V (high mass, start/end). Charged ions (+1, +2, +3) were selected in order to be submitted to the MSMS fragmentation over the *m/z* range from 50 to 2,000 with a scan time of 0.25 sec. For the post-acquisition lock mass correction of the data in the MS method, the doubly-charged monoisotopic ion of [Glu^1^]-fibrinopeptide B was used at 100 fmol/μL using the reference sprayer of the nanoESI source with a frequency of 30s at 0.5 μL/min into the mass spectrometer.

### ESI-QTOF data processing

Data were processed with UNIFI (Waters) and MassLynx (Waters) software packages using known peptide sequences to do the MSMS fragments search and the spectra profile comparison.

### Peptide degradation assays

sXIP and derivatives were incubated at 500 nM with PepF at different concentrations ranging from 100 to 0.025 nM during 4 h at 37°C as described in the previous section. 30 μL of the mix was then added to 270 μL of exponential phase culture of a P_*comS*_ reporter strain defective for the pheromone (P_*comS*_*-luxAB* Δ*comS*) and maximum luciferase activity was computed as reported above. Maximum values obtained were normalized by the signal recorded for the peptide incubated without the peptidase and enzyme concentration affording half luminescence (EC_50_) was extrapolated using Graphpad Prism and the non-linear extrapolation equation:

$$Signal=b+\frac{\left(t-b\right)}{\left(1+\frac{{IC}_{50}}{{\left[PepF\right]}^{HS}}\right)}$$

where *t* stands for top, *b* for bottom, and *HS* for Hill-Slope.

### Electrophoretic mobility shift assays (EMSAs)

A fixed concentration of purified ComR protein was mixed with a 30-bp dsDNA fragment (20 ng) carrying the ComR box of P_*comX*_ coupled to the Cy3 fluorophore in absence or presence of increasing amounts of synthetic XIP peptide variants as previously described [58]. Purified StrepTagged ComR (3 μM) was mixed with twofold serial dilutions of XIP variants at an initial concentration of 6 μM. The samples (20 μL) prepared in binding buffer (20 mM Tris·HCl, pH 8.0, 150 mM NaCl, 1 mM EDTA, 10% glycerol [v/v], 1 mg/mL BSA) were incubated at 37°C for 10 min prior to run at 100 V for approximately 2 h on a native 4–20% gradient gel (iD PAGE gel; Eurogentec) in Tris-MOPS buffer (50 mM Tris-base, 50 mM 3-(*N*-morpholino) propanesulfonic acid, 1 mM EDTA, pH 7.7). DNA complexes were detected by fluorescence on the Amersham Typhoon Scanner with bandpass excitation and emission filters of 540/25 and 595/25 nm, respectively (GE Healthcare).

## Supporting information

S1 Fig Mathematical model validation. (A) ComR, ComS, ComRS and ComX intracellular concentrations computed over time regarding different fold changes in ComR abundance. Fold changes and kinetics of ComR abundance were computed based on experimental data (i.e. immunoblotting semi-quantification and luciferase activity of a P_*comR*_*-luxAB* reporter strain). ComS, ComRS and ComX abundance (basal and upon activation) were inferred from a previous model of *S*. *thermophilus*, luciferase activity (P_*comS*_*-luxAB* and P_*comX*_*-luxAB* reporter strains) and biochemical characterization (see S1 Appendix for complete methodology). (B) and (C) Validation of the model using single-cell microscopy data displaying the percentage of P_*comX*_-activated cells in the population related to fold change in ComR abundance computed with immunoblotting semi-quantification. The model uses as a source of heterogeneity either uneven number of ComS (in panel B) or ComR (in panel C). The distribution of those players in the population was computed thanks to single-cell distribution of P_*comS*_*-gfp*^*+*^ or P_*comR*_*-gfp*^*+*^ activation, respectively.
(TIF)

S2 Fig Effect of the deletion of ComX-regulated peptidases on competence activation. Specific luciferase activity measured for WT (with P_*comX*_*-luxAB* as proxy) compared to strains where *pepO*, *pepP or pepQ* are deleted. Competence was activated with *comR* overexpression thanks to a xylose-inducible promoter fused to *comR* (P_*xyl2*_*-comR*, 0.5% xylose). Dots show biological triplicates, the bar shows the mean, and error bar denotes standard deviation. One-way ANOVA with Dunett’s test were performed for each condition in comparison to WT to generate *P* values (ns, non-significative).
(TIF)

S3 Fig Progressive inhibition of *pepF*. (A) Kinetics of P_*comX*_ activation in a strain harboring a P_*comX*_*-luxAB* reporter system with a dCas9 module targeting *pepF* (P_F6_-*lacI* P_*lac*_*-dcas9* P_3_-*gRNA*_*9*) and a xylose-inducible ComR module (P_*xyl2*_*-comR*). Cells were incubated with increasing IPTG concentrations (0, 0.27, 0.82, 2.47, 7.41 μM) and 0.25% xylose. Data show the mean specific luciferase activity (RLU/OD_600_, solid lines) and mean growth (OD_600_, dashed lines) for biological triplicates. Shaded lines denote standard deviation. (B) Total specific luciferase activity of the experiment reported in panel A for cells incubated with increasing IPTG concentrations (0, 0.01, 0.03, 0.09, 0.27, 0.82, 2.47, 7.41, 22, 66 and 200 μM) and 0.25% xylose. The bars denote the mean and the dots biological triplicates.
(TIF)

S4 Fig Screening of sXIP variants for competence activation and susceptibility to PepF degradation. Specific maximum luciferase activity (RLU/OD_600_) upon peptide addition is plotted against the loss of signal with the peptide pre-incubated with PepF (plotted as a percentage of signal loss). A total number of 20 sXIP variants of the native *S*. *salivarius* sXIP (LPYFAGCL) were tested. The different peptides (500 nM) were incubated during 4 h at 37°C with or without PepF (7 nM). The reaction mixture was then 10-fold diluted by addition to an exponential growing culture (CDM) of the reporter strain defective for the genetically-encoded pheromone XIP (P_*comS*_*-luxAB* Δ*comS*). Dots represents mean of technical triplicates. The specific sequence of the peptide is displayed with mutated residues in red. Red/blue dots denote peptides discarded/selected for subsequent characterization due to their weak/strong induction of competence.
(TIF)

S5 Fig Susceptibility of sXIP variants to PepF degradation (complete dataset). Maximum luciferase activities of a reporter strain defective for the genetically-encoded pheromone XIP (P_*comS*_*-luxAB* Δ*comS*). sXIP (500 nM) was incubated at 37°C for 4 h with increasing concentrations of PepF (0, 0.025, 0.065, 0.16, 0.4, 1, 2.56, 6.4, 16, 40, and 100 nM). The reaction mixture was then 10-fold diluted by addition to an exponential growing culture (CDM) of the reporter strain. Maximum specific luciferase activity (RLU/OD_600_) is displayed as the percentage of signal in comparison to an addition of sXIP without PepF digestion. Dots represent technical replicates. The curve is a non-linear fit of inhibition.
(TIF)

S6 Fig Electrophoretic mobility shift assays of ComR·sXIP binding to P_*comX*_. Labeled P_*comX*_ 30-bp DNA fragments (20 ng) were incubated with a fixed concentration of ComR_Ssa_ WT (3 μM) in absence of peptide (−) or in presence of increasing concentrations of sXIP variant (0, 0.9, 0.19, 0.38, 0.75, 1.5, 3, and 6 μM). The ComR-XIP-DNA complex and multimeric complexes are indicated by arrowheads.
(TIF)

S7 Fig Kinetics of competence activation with sXIP variants. Data show specific luciferase activity (RLU/OD_600_, solid lines) and growth (OD_600_, dashed lines) measured with a P_*comS*_*-luxAB* reporter strain defective for the pheromone (Δ*comS*) supplemented with sXIP variants (5 nM) at t = 120 min. Shaded lines represent standard deviations.
(TIF)

S8 Fig PepF essentiality is not competence-related. Maximum OD_600_ measured after growth with (PepF |-) or without (Control) *pepF* inhibition in strains with the complete set of competence genes (WT) or deficient for activation of early (Δ*comRS*) or late (Δ*comX*) competence genes. In the three strains, *pepF* was inhibited thanks to a guide targeting the promoter of *pepF* (P_3_*-gRNA_9*) together with the IPTG-inducible dCas9 system (P_F6_*-lacI* P_*lac*_*-dcas9*) induced at 1 mM IPTG. Statistical *t*-test was performed for each strain in comparison to the related control and one-way ANOVA with Dunett’s test were performed to compare the *pepF*-inhibited strains with WT to generate *P* values (***, *P* < 0.001; ****, *P* < 0.0001; ns, non-significative).
(TIF)

S9 Fig Conservation of PepF in salivarius streptococci and susceptibility of *S*. *vestibularis* sXIP to PepF degradation. (A) Alignment of PepF performed with CLC Main Workbench multiple alignment tool (http://www.clcbio.com/products/clc-main-workbench/). PepF from 4 representative strains (i.e. *S*. *salivarius* HSISS4, *S*. *thermophilus* LMD-9, *S*. *thermophilus* LMG18311 and *S*. *vestibularis* NTC12167) were used for the alignment. Red, orange, and yellow denote 100%, 75%, and 50% conservation, respectively. The conserved Zinc-binding motif HEXXH is underlined. (B) Maximum luciferase activities of a *S*. *thermophilus* LMD-9 reporter strain defective for the genetically-encoded pheromone XIP and harboring *S*. *vestibularis* ComR (P_*comS*_*-luxAB* Δ*comS comR*_*Sve*_). sXIP_Sve_ (500 nM) was incubated at 37°C for 4 h with PepF_Ssa_ at concentrations of 0, 0.025, 0.065, 0.16, 0.4, 1, 2.56, 6.4, 16, 40, and 100 nM. The reaction mixture was then 10-fold diluted in a CDM exponential growing culture of the reporter strain. Maximum specific luciferase activity (RLU/OD_600_) is displayed as the percentage of signal in comparison to an addition of sXIP_Sve_ without PepF digestion. Dots represent technical replicates. The curve is a non-linear fit of inhibition. The enzyme concentration affording half luminescence (EC_50_) is 3.06 ± 0.74.
(TIF)

S10 Fig Synteny analysis of *pepF* in *Firmicutes*. The HSISS4 sequence of PepF was used as a proxy for genomic context analysis of *pepF* in *Firmicutes* thanks to the SyntTax server [4]. Species were selected from various genera across *Firmicutes*. Central bold arrow: *pepF* homologs, purple: *coiA* homologs, left light blue: *sapR* homologs (transcriptional regulator from LysR family), light green: hypothetical, yellow: hypothetical, right light blue: *oxlT* homologs, right green: O-methyltransferase family protein C1 homologs, pink: *prtM* homologs, blue: hypothetical protein, and green: *alaS* homologs.
(TIF)

S1 Table List of bacterial strains used in this study. (PDF)

S2 Table List of plasmids used in this study. (PDF)

S3 Table List of oligonucleotides used in this study. (PDF)

S4 Table List of PCR fragments, synthetic DNA constructs, and EMSA probes. (PDF)

S1 Appendix Mathematical model of the ComRS system in *Streptococcus salivarius*. (PDF)

S2 Appendix MATLAB code for the mathematical model. (DOCX)

S1 Dataset Specific experimental data used for mathematical model calibration and validation. (XLSX)

S2 Dataset Numerical values of experimental data shown in Figs 2–6 and S2–S5, S7 and S8 Figs. (XLSX)
