# Supplementary figures and images for: Competence shut-off by intracellular pheromone degradation in salivarius streptococci
Source: PLoS Genet. 2022 May 25;18(5):e1010198. doi: 10.1371/journal.pgen.1010198 (PMC9173638; doi:10.1371/journal.pgen.1010198)

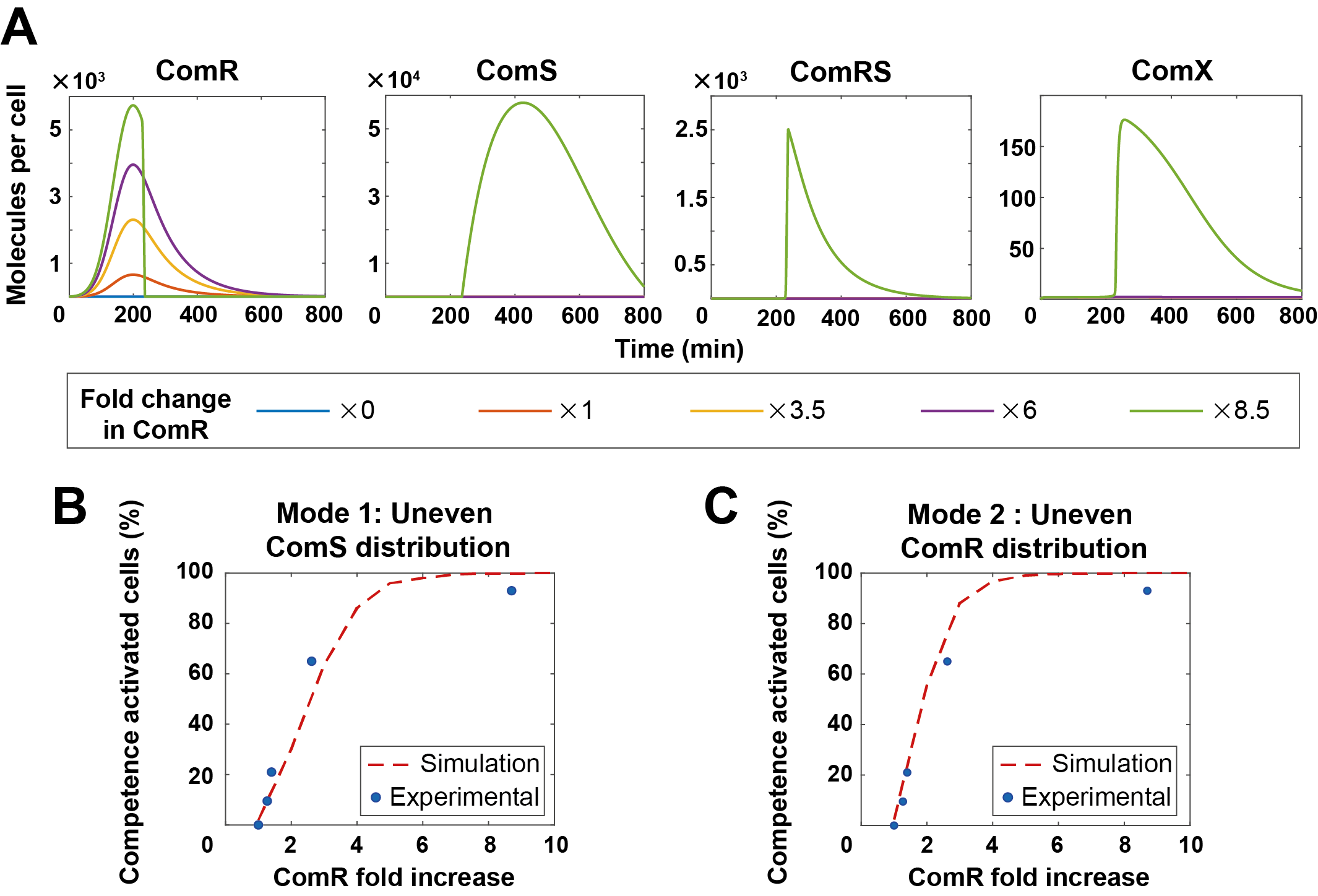

Supplement: S1 Fig — (A) ComR, ComS, ComRS and ComX intracellular concentrations computed over time regarding different fold changes in ComR abundance. Fold changes and kinetics of ComR abundance were computed based on experimental data (i.e. immunoblotting semi-quantification and luciferase activity of a PcomR-luxAB reporter strain). ComS, ComRS and ComX abundance (basal and upon activation) were inferred from a previous model of S. thermophilus, luciferase activity (PcomS-luxAB and PcomX-luxAB reporter strains) and biochemical characterization (see S1 Appendix for complete methodology). (B) and (C) Validation of the model using single-cell microscopy data displaying the percentage of PcomX-activated cells in the population related to fold change in ComR abundance computed with immunoblotting semi-quantification. The model uses as a source of heterogeneity either uneven number of ComS (in panel B) or ComR (in panel C). The distribution of those players in the population was computed thanks to single-cell distribution of PcomS-gfp+ or PcomR-gfp+ activation, respectively. (TIF) [file pgen.1010198.s001.tif]

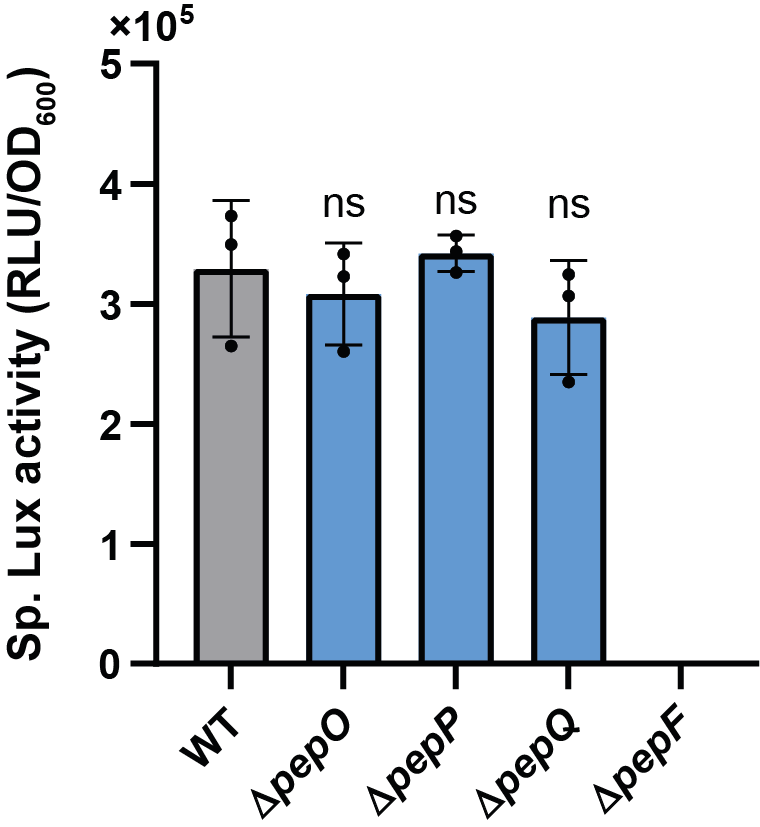

Supplement: S2 Fig — Specific luciferase activity measured for WT (with PcomX-luxAB as proxy) compared to strains where pepO, pepP or pepQ are deleted. Competence was activated with comR overexpression thanks to a xylose-inducible promoter fused to comR (Pxyl2-comR, 0.5% xylose). Dots show biological triplicates, the bar shows the mean, and error bar denotes standard deviation. One-way ANOVA with Dunett’s test were performed for each condition in comparison to WT to generate P values (ns, non-significative). (TIF) [file pgen.1010198.s002.tif]

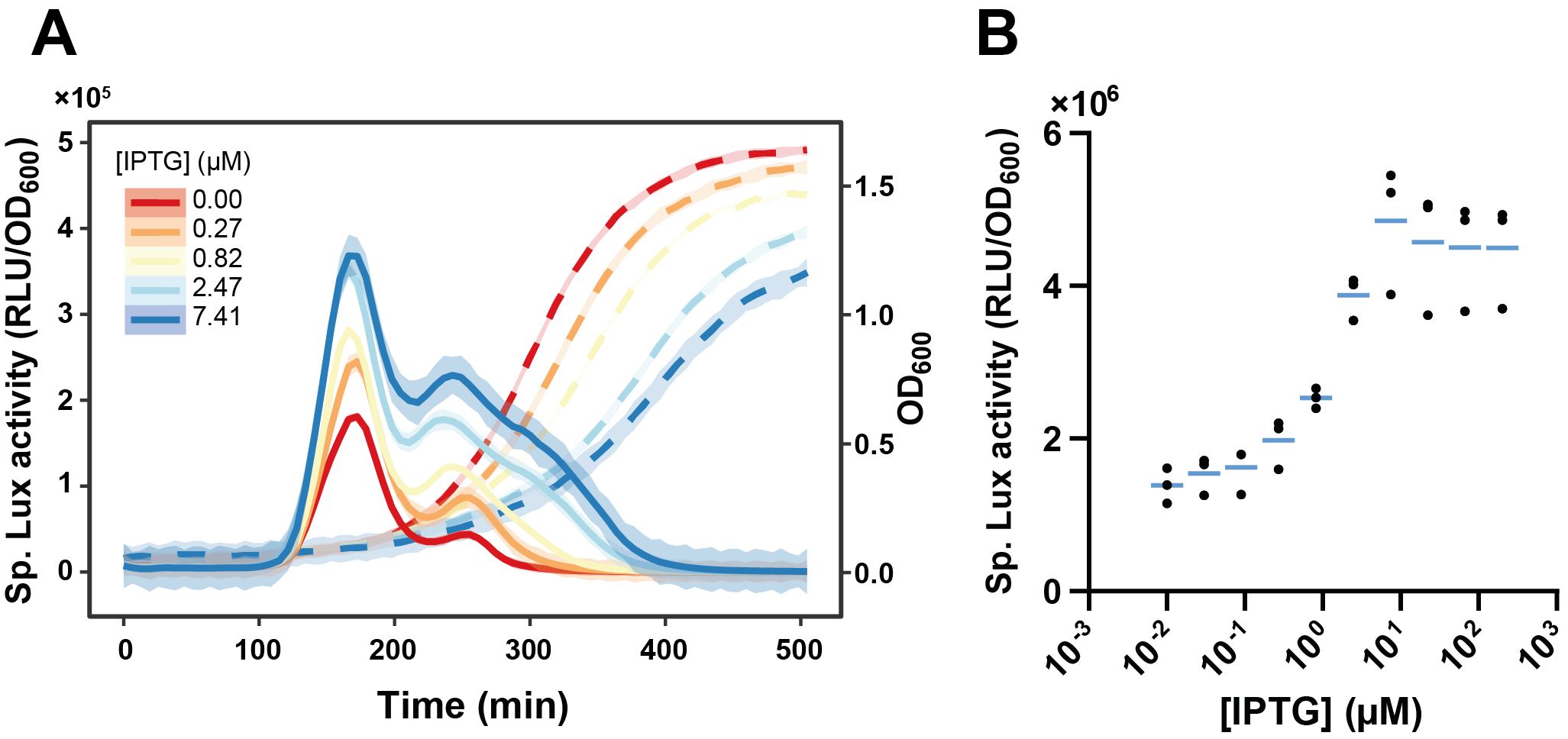

Supplement: S3 Fig — (A) Kinetics of PcomX activation in a strain harboring a PcomX-luxAB reporter system with a dCas9 module targeting pepF (PF6-lacI Plac-dcas9 P3-gRNA_9) and a xylose-inducible ComR module (Pxyl2-comR). Cells were incubated with increasing IPTG concentrations (0, 0.27, 0.82, 2.47, 7.41 μM) and 0.25% xylose. Data show the mean specific luciferase activity (RLU/OD600, solid lines) and mean growth (OD600, dashed lines) for biological triplicates. Shaded lines denote standard deviation. (B) Total specific luciferase activity of the experiment reported in panel A for cells incubated with increasing IPTG concentrations (0, 0.01, 0.03, 0.09, 0.27, 0.82, 2.47, 7.41, 22, 66 and 200 μM) and 0.25% xylose. The bars denote the mean and the dots biological triplicates. (TIF) [file pgen.1010198.s003.tif]

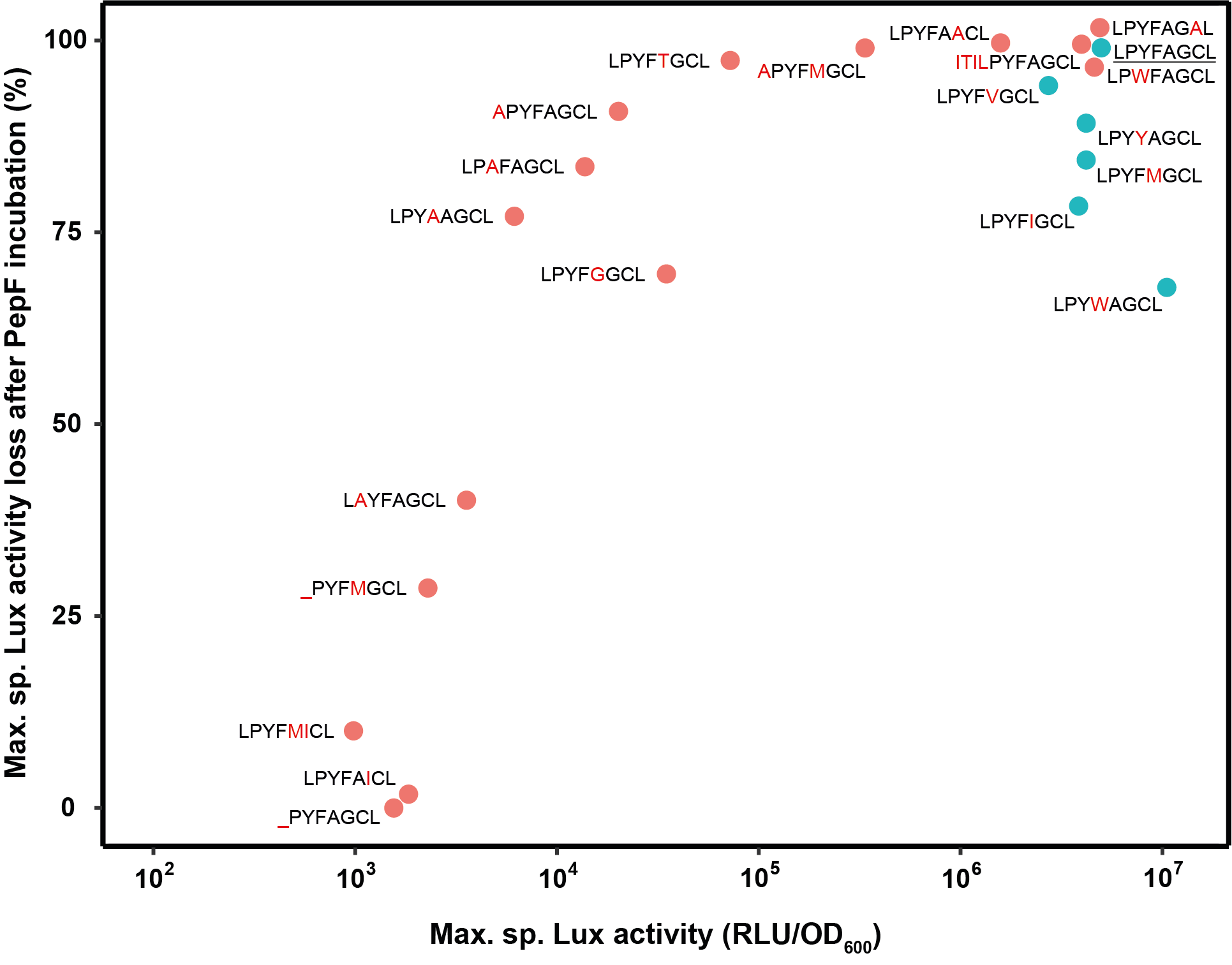

Supplement: S4 Fig — Specific maximum luciferase activity (RLU/OD600) upon peptide addition is plotted against the loss of signal with the peptide pre-incubated with PepF (plotted as a percentage of signal loss). A total number of 20 sXIP variants of the native S. salivarius sXIP (LPYFAGCL) were tested. The different peptides (500 nM) were incubated during 4 h at 37°C with or without PepF (7 nM). The reaction mixture was then 10-fold diluted by addition to an exponential growing culture (CDM) of the reporter strain defective for the genetically-encoded pheromone XIP (PcomS-luxAB ΔcomS). Dots represents mean of technical triplicates. The specific sequence of the peptide is displayed with mutated residues in red. Red/blue dots denote peptides discarded/selected for subsequent characterization due to their weak/strong induction of competence. (TIF) [file pgen.1010198.s004.tif]

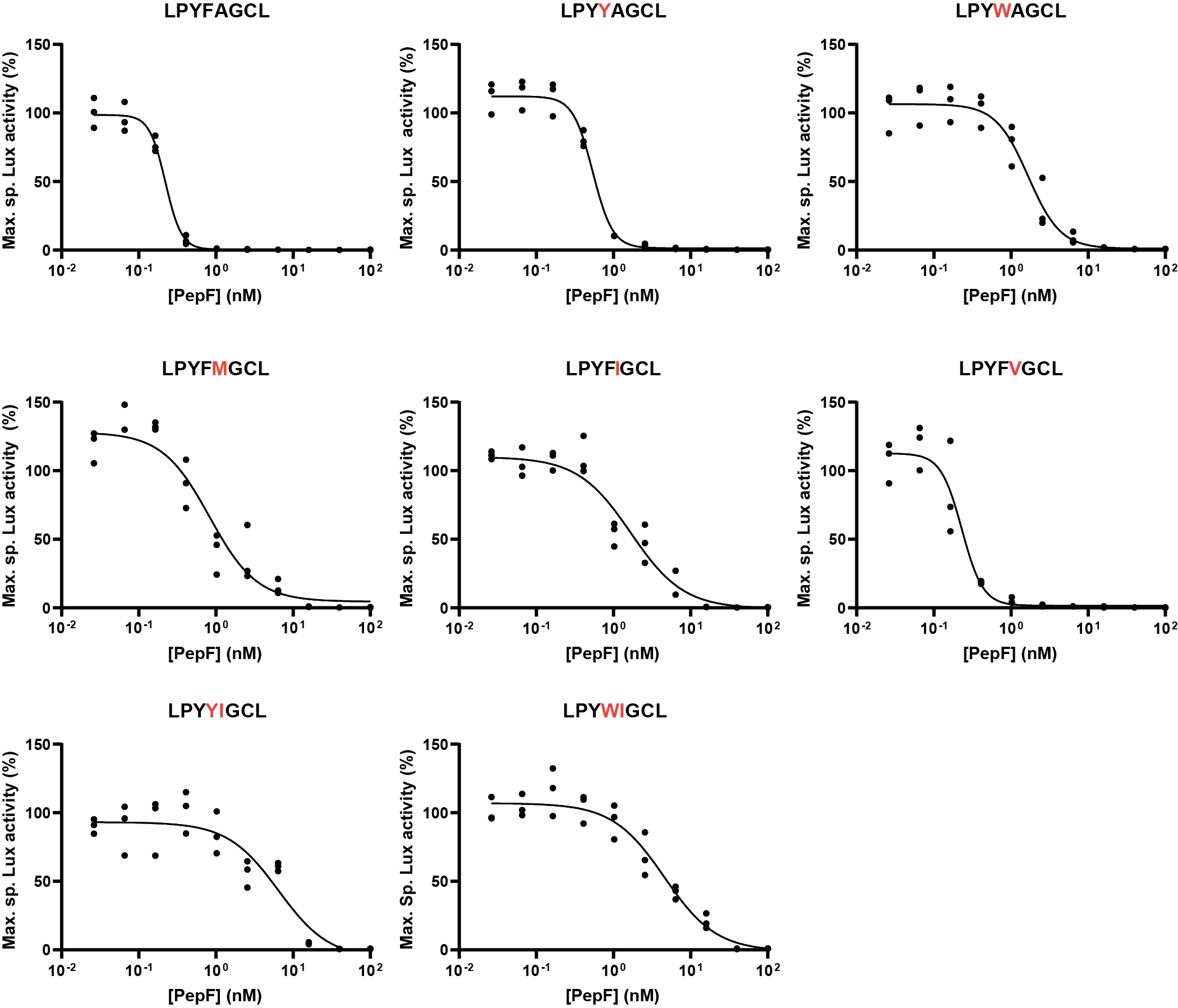

Supplement: S5 Fig — Maximum luciferase activities of a reporter strain defective for the genetically-encoded pheromone XIP (PcomS-luxAB ΔcomS). sXIP (500 nM) was incubated at 37°C for 4 h with increasing concentrations of PepF (0, 0.025, 0.065, 0.16, 0.4, 1, 2.56, 6.4, 16, 40, and 100 nM). The reaction mixture was then 10-fold diluted by addition to an exponential growing culture (CDM) of the reporter strain. Maximum specific luciferase activity (RLU/OD600) is displayed as the percentage of signal in comparison to an addition of sXIP without PepF digestion. Dots represent technical replicates. The curve is a non-linear fit of inhibition. (TIF) [file pgen.1010198.s005.tif]

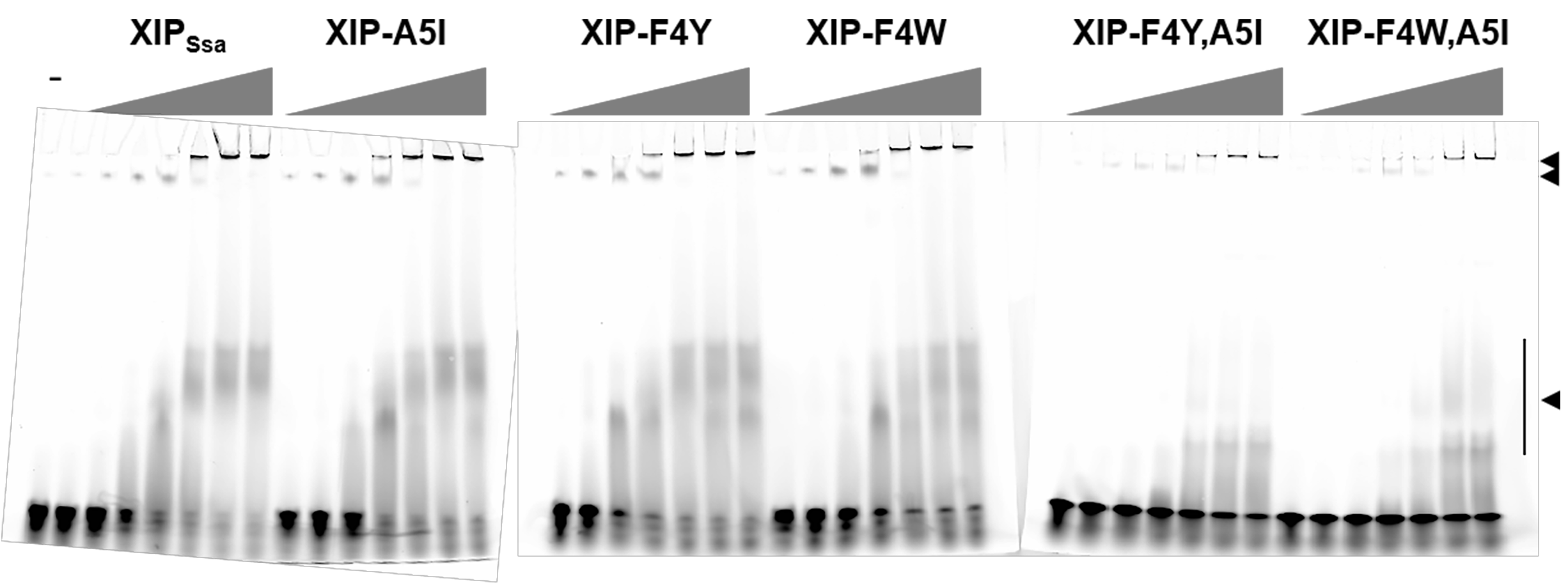

Supplement: S6 Fig — Labeled PcomX 30-bp DNA fragments (20 ng) were incubated with a fixed concentration of ComRSsa WT (3 μM) in absence of peptide (−) or in presence of increasing concentrations of sXIP variant (0, 0.9, 0.19, 0.38, 0.75, 1.5, 3, and 6 μM). The ComR-XIP-DNA complex and multimeric complexes are indicated by arrowheads. (TIF) [file pgen.1010198.s006.tif]

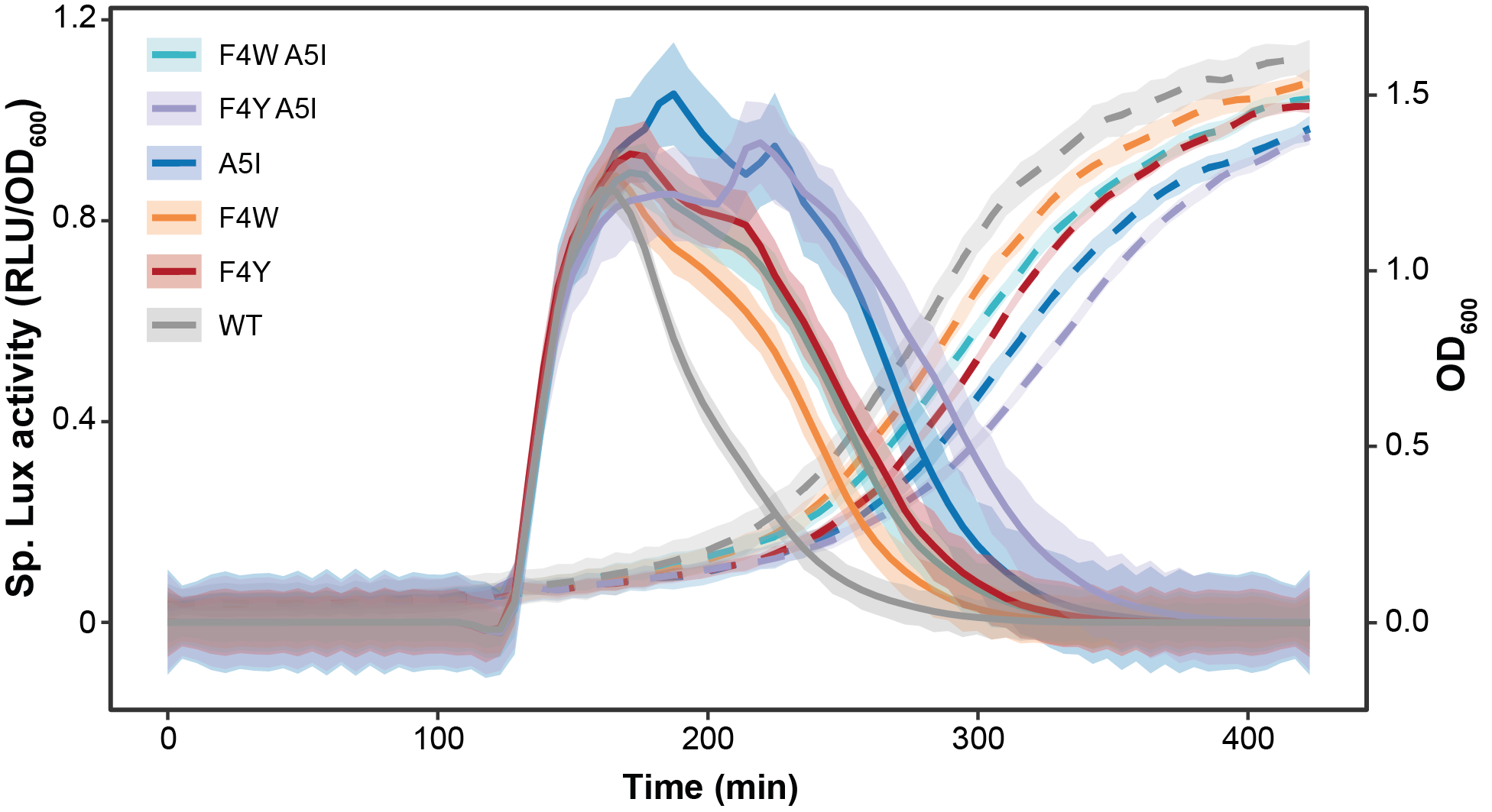

Supplement: S7 Fig — Data show specific luciferase activity (RLU/OD600, solid lines) and growth (OD600, dashed lines) measured with a PcomS-luxAB reporter strain defective for the pheromone (ΔcomS) supplemented with sXIP variants (5 nM) at t = 120 min. Shaded lines represent standard deviations. (TIF) [file pgen.1010198.s007.tif]

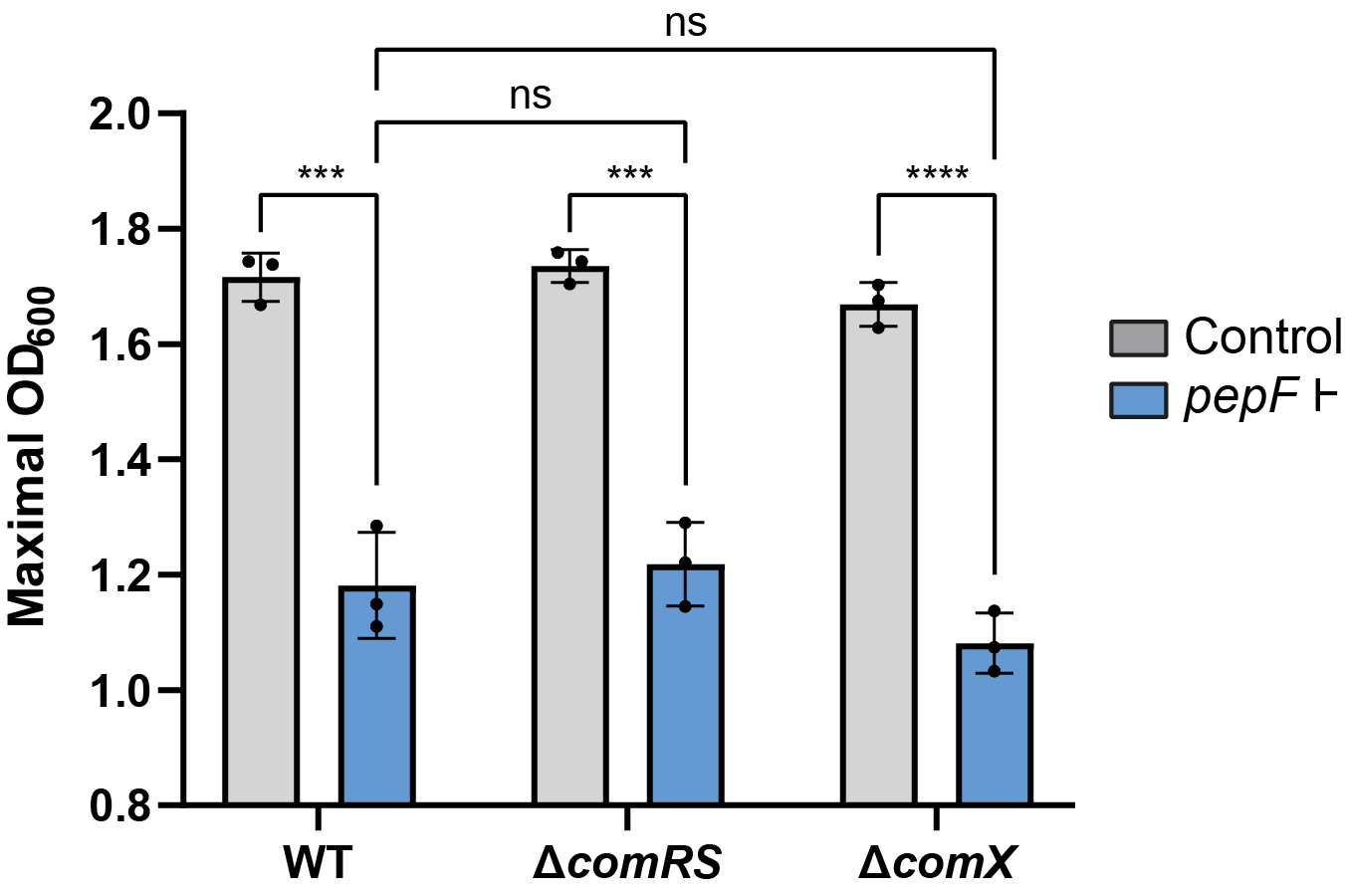

Supplement: S8 Fig — Maximum OD600 measured after growth with (PepF |-) or without (Control) pepF inhibition in strains with the complete set of competence genes (WT) or deficient for activation of early (ΔcomRS) or late (ΔcomX) competence genes. In the three strains, pepF was inhibited thanks to a guide targeting the promoter of pepF (P3-gRNA_9) together with the IPTG-inducible dCas9 system (PF6-lacI Plac-dcas9) induced at 1 mM IPTG. Statistical t-test was performed for each strain in comparison to the related control and one-way ANOVA with Dunett’s test were performed to compare the pepF-inhibited strains with WT to generate P values (***, P < 0.001; ****, P < 0.0001; ns, non-significative). (TIF) [file pgen.1010198.s008.tif]

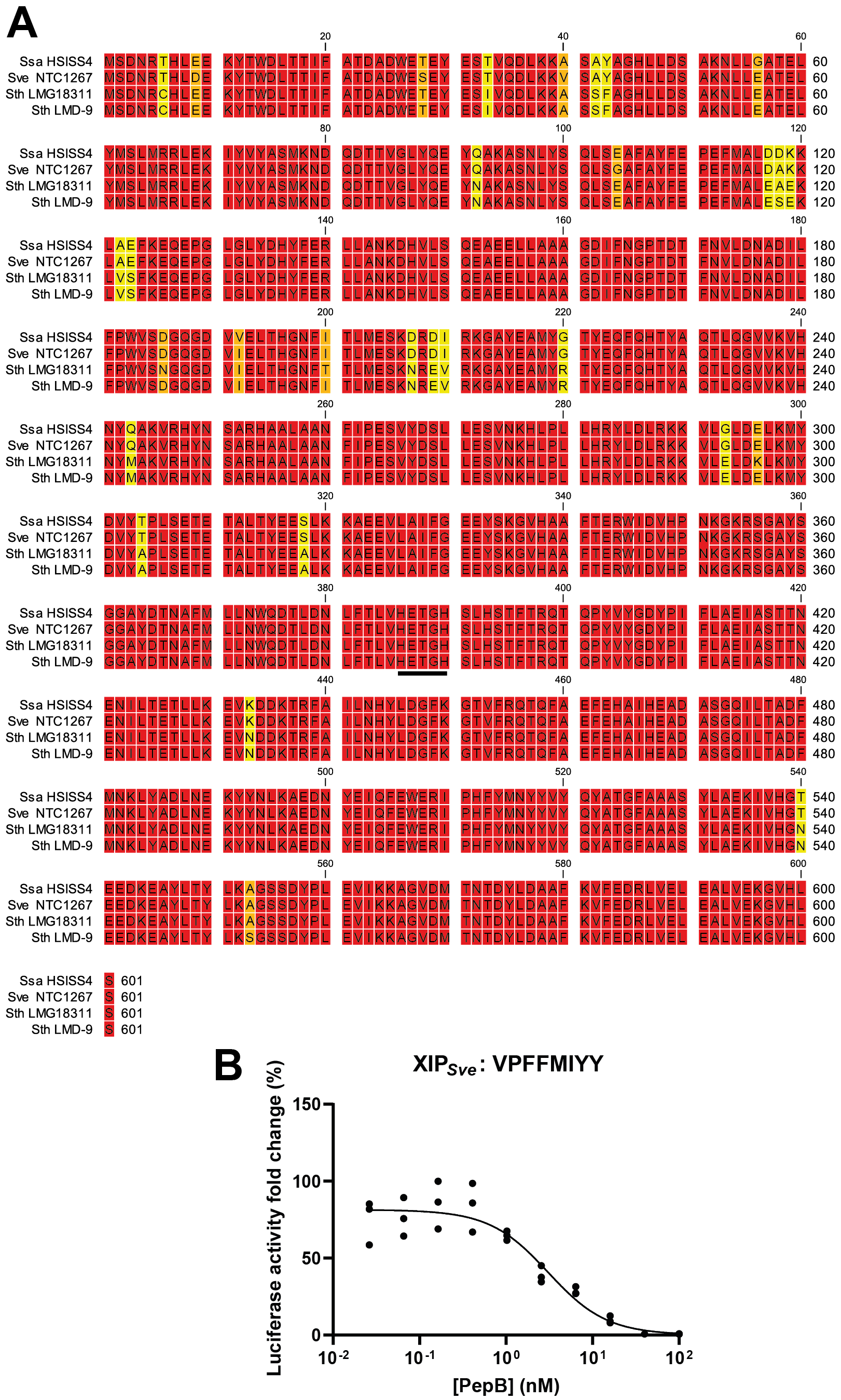

Supplement: S9 Fig — (A) Alignment of PepF performed with CLC Main Workbench multiple alignment tool (http://www.clcbio.com/products/clc-main-workbench/). PepF from 4 representative strains (i.e. S. salivarius HSISS4, S. thermophilus LMD-9, S. thermophilus LMG18311 and S. vestibularis NTC12167) were used for the alignment. Red, orange, and yellow denote 100%, 75%, and 50% conservation, respectively. The conserved Zinc-binding motif HEXXH is underlined. (B) Maximum luciferase activities of a S. thermophilus LMD-9 reporter strain defective for the genetically-encoded pheromone XIP and harboring S. vestibularis ComR (PcomS-luxAB ΔcomS comRSve). sXIPSve (500 nM) was incubated at 37°C for 4 h with PepFSsa at concentrations of 0, 0.025, 0.065, 0.16, 0.4, 1, 2.56, 6.4, 16, 40, and 100 nM. The reaction mixture was then 10-fold diluted in a CDM exponential growing culture of the reporter strain. Maximum specific luciferase activity (RLU/OD600) is displayed as the percentage of signal in comparison to an addition of sXIPSve without PepF digestion. Dots represent technical replicates. The curve is a non-linear fit of inhibition. The enzyme concentration affording half luminescence (EC50) is 3.06 ± 0.74. (TIF) [file pgen.1010198.s009.tif]

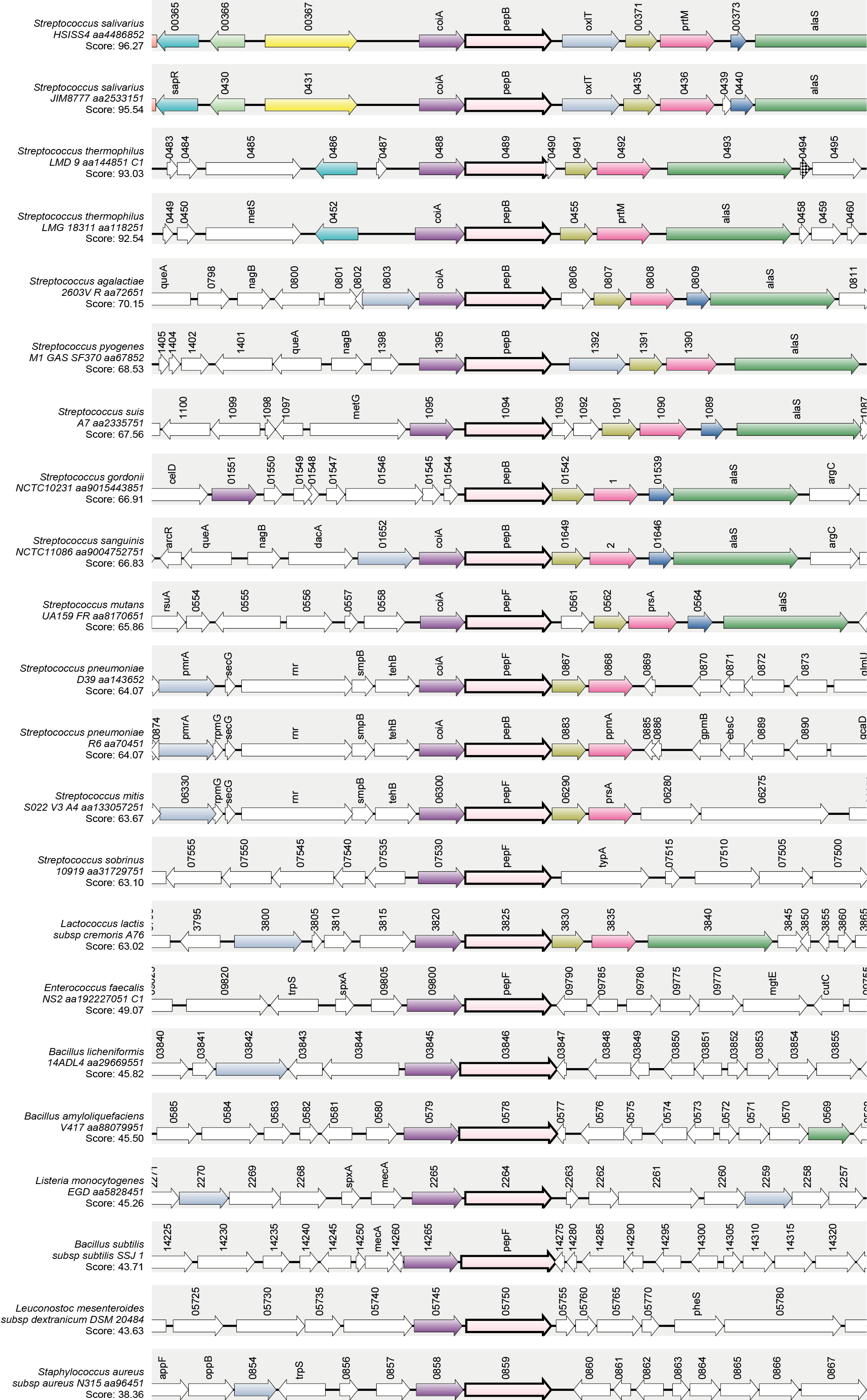

Supplement: S10 Fig — The HSISS4 sequence of PepF was used as a proxy for genomic context analysis of pepF in Firmicutes thanks to the SyntTax server [4]. Species were selected from various genera across Firmicutes. Central bold arrow: pepF homologs, purple: coiA homologs, left light blue: sapR homologs (transcriptional regulator from LysR family), light green: hypothetical, yellow: hypothetical, right light blue: oxlT homologs, right green: O-methyltransferase family protein C1 homologs, pink: prtM homologs, blue: hypothetical protein, and green: alaS homologs. (TIF) [file pgen.1010198.s010.tif]
