# Supplementary material for: Competence shut-off by intracellular pheromone degradation in salivarius streptococci
Source: PLoS Genet. 2022 May 25;18(5):e1010198. doi: 10.1371/journal.pgen.1010198 (PMC9173638; doi:10.1371/journal.pgen.1010198)
Supplement: S1 Table — (PDF) [file pgen.1010198.s011.pdf]

Table S1. List of bacterial strains used in this study

Strain	Characteristics	Reference/source
Escherichia coli		
TOP10	*mcrA*, Δ(*mrr-hsd*RMS-*mcrBC*), Phi80*lacZ(del)M15*, Δ*lacX74*, *deoR*, *recA1*, *araD139*, Δ(*ara-leu*)7697, *galU*, *galK*, *rpsL(SmR)*, *endA1*, *nupG*	Invitrogen, CA
Streptococcus salivarius		
HSISS4	Wild-type gastro-intestinal tract isolate	[1]
AK0004	*HSISS4 tRNAthr::PcomX-luxAB-spc*<br>*$tRNA_{ser}$::P_{xyl2}-comR-cat*	[2]
AK0010	*HSISS4 tRNAthr::PcomR-luxAB-spc*	[2]
AK0022	*HSISS4 tRNAthr::PcomR-opt-gfp-spc*	[2]
AK0043	*HSISS4 tRNAthr::PcomS-opt-gfp-spc*	This work
AK0044	*HSISS4 tRNAthr::PcomS-luxAB-spc*<br>*$tRNA_{ser}$::P_{xyl2}-comR-cat*	This work
AK0045	HSISS4 *$tRNA_{thr}$::P_{comX}-luxAB-lox72*<br>*$tRNA_{ser}$::P_{xyl2}-comR-lox72*<br>*tnpII::P_{32}-opt-pepF-cat*	This work
AK0046	HSISS4 *$tRNA_{thr}$::*P_{F6}-*lacI-lox72*<br>*$tRNA_{ser}$::*P_{lac}-*dcas9-lox72*<br>*tnpII::P_{comX}-luxAB-lox72*<br>*SUC::P_{xyl2}-comR-lox72*	This work
AK0047	HSISS4 *$tRNA_{thr}$::*P_{F6}-*lacI-lox72*<br>*$tRNA_{ser}$::*P_{lac}-*dcas9-lox72*<br>*tnpII::P_{comX}-luxAB-lox72*<br>*SUC::P_{xyl2}-comR-lox72*<br>*GOR::P3-g_9(PpepF)-erm*	This work
AK0048	HSISS4 *$tRNA_{thr}$::P_{comX}-luxAB-lox72*<br>*$tRNA_{ser}$::P_{xyl2}-comR-lox72*<br>Δ*pepO::cat*	This work
AK0049	HSISS4 *$tRNA_{thr}$::P_{comX}-luxAB-lox72*<br>*$tRNA_{ser}$::P_{xyl2}-comR-lox72*<br>Δ*pepP::cat*	This work
AK0050	HSISS4 *$tRNA_{thr}$::P_{comX}-luxAB-lox72*<br>*$tRNA_{ser}$::P_{xyl2}-comR-lox72*<br>Δ*pepQ::cat*	This work
AK0051	*HSISS4 tRNAthr::PcoiA-luxAB-spc*	This work
AK0052	*HSISS4 tRNAthr::PpepF-luxAB-spc*	This work
AK0053	*HSISS4 tRNAthr::PcoiA-coiA-PpepF-luxAB-spc*	This work

AK0054	HSISS4 *tRNA*$_{thr}$::*P*$_{comX}$*-luxAB-lox72*<br>*tRNA*$_{ser}$::*P*$_{xyl2}$*-comR-lox72*<br>*ΔcomX::erm*	This work
AK0055	HSISS4 *tRNA*$_{thr}$::*P*$_{comX}$*-luxAB-lox72*<br>*tRNA*$_{ser}$::*P*$_{xyl2}$*-comR-lox72*<br>*ΔPcoiA::spc*	This work
AK0056	HSISS4 *tRNA*$_{thr}$::*P*$_{comX}$*-luxAB-lox72*<br>*tRNA*$_{ser}$::*P*$_{xyl2}$*-comR-lox72*<br>*ΔPcoiA::spc*<br>*FBA::*P$_{coiA}$*-coiA-P*$_{pepF}$*-pepF*	This work
AK0057	HSISS4 *tRNA*$_{thr}$::P$_{F6}$*-lacI-lox72*<br>*tRNA*$_{ser}$::P$_{lac}$*-dcas9-lox72*<br>*tnpII::P*$_{comX}$*-luxAB-lox72*<br>*SUC::P*$_{xyl2}$*-comR-lox72*<br>*GOR::P3-g_9(PpepF)-erm*<br>Δ*comR-comS*$_{WT}$	This work
AK0058	HSISS4 *tRNA*$_{thr}$::P$_{F6}$*-lacI-lox72*<br>*tRNA*$_{ser}$::P$_{lac}$*-dcas9-lox72*<br>*tnpII::P*$_{comX}$*-luxAB-lox72*<br>*SUC::P*$_{xyl2}$*-comR-lox72*<br>*GOR::P3-g_9(PpepF)-erm*<br>Δ*comR-comS*$_{F4Y}$	This work
AK0059	HSISS4 *tRNA*$_{thr}$::P$_{F6}$*-lacI-lox72*<br>*tRNA*$_{ser}$::P$_{lac}$*-dcas9-lox72*<br>*tnpII::P*$_{comX}$*-luxAB-lox72*<br>*SUC::P*$_{xyl2}$*-comR-lox72*<br>*GOR::P3-g_9(PpepF)-erm*<br>Δ*comR-comS*$_{F4W}$	This work
AK0060	HSISS4 *tRNA*$_{thr}$::P$_{F6}$*-lacI-lox72*<br>*tRNA*$_{ser}$::P$_{lac}$*-dcas9-lox72*<br>*tnpII::P*$_{comX}$*-luxAB-lox72*<br>*SUC::P*$_{xyl2}$*-comR-lox72*<br>*GOR::P3-g_9(PpepF)-erm*<br>Δ*comR-comS*$_{A5I}$	This work
AK0061	HSISS4 *tRNA*$_{thr}$::P$_{F6}$*-lacI-lox72*<br>*tRNA*$_{ser}$::P$_{lac}$*-dcas9-lox72*<br>*tnpII::P*$_{comX}$*-luxAB-lox72*<br>*SUC::P*$_{xyl2}$*-comR-lox72*<br>*GOR::P3-g_9(PpepF)-erm*<br>Δ*comR-comS*$_{F4YA5I}$	This work

AK0062	HSISS4 $tRNA_{thr}$::P_{F6}-*lacI-lox72*<br>$tRNA_{ser}$::P_{lac}-*dcas9-lox72*<br>*tnpII::*P_{comX}*-luxAB-lox72*<br>*SUC::*P_{xyl2}*-comR-lox72*<br>*GOR::P3-g_9(PpepF)-erm*<br>Δ*comR-comS*$_{F4WA5I}$	This work
AK0063	HSISS4 $tRNA_{thr}$::P_{F6}-*lacI-lox72*<br>$tRNA_{ser}$::P_{lac}-*dcas9-lox72*<br>*tnpII::*P_{comX}*-luxAB-lox72*<br>*SUC::*P_{xyl2}*-comR-lox72*<br>*GOR::P3-g_9(PpepF)-erm*<br>Δ*comRS ::cat*	This work
AK0064	HSISS4 $tRNA_{thr}$::P_{F6}-*lacI-lox72*<br>$tRNA_{ser}$::P_{lac}-*dcas9-lox72*<br>*tnpII::*P_{comX}*-luxAB-lox72*<br>*SUC::*P_{xyl2}*-comR-lox72*<br>*GOR::P3-g_9(PpepF)-erm*<br>Δ*comX ::cat*	This work
LL0052	*HSISS4 tRNAthr::PcomS-luxAB-spc*	This work
LL053	*HSISS4 tRNAthr::PcomS-luxAB-spc*<br>Δ*comS*	This work
Streptococcus thermophilus		
LL01	*LMD-9 (blpD-blpX)::PcomS-luxAB*<br>*comR::comR*$_{Sve}$<br>Δ*comS::*P_{32}-*cat*	[3]

References

1. Van den Bogert B, Boekhorst J, Herrmann R, Smid EJ, Zoetendal EG, Kleerebezem M. Comparative genomics analysis of Streptococcus isolates from the human small intestine reveals their adaptation to a highly dynamic ecosystem. PLoS One. 2013; 8(12):e83418.

2. Knoops A, Vande Capelle F, Fontaine L, Verhaegen M, Mignolet J, Goffin P et al. The CovRS environmental sensor directly controls the ComRS signaling system to orchestrate competence bimodality in salivarius streptococci. mBio. 2022 Jan 4; e0312521.

3. Ledesma-Garcia L, Thuillier J, Guzman-Espinola A, Ensinck I, de la Sierra-Gallay IL, Lazar N, et al. Molecular dissection of pheromone selectivity in the competence signaling system ComRS of streptococci. Proc Natl Acad Sci U S A. 2020;117: 7745–7754.
