# Supplementary material for: Competence shut-off by intracellular pheromone degradation in salivarius streptococci
Source: PLoS Genet. 2022 May 25;18(5):e1010198. doi: 10.1371/journal.pgen.1010198 (PMC9173638; doi:10.1371/journal.pgen.1010198)
Supplement: S2 Table — (PDF) [file pgen.1010198.s012.pdf]

Table S2. List of plasmids used in this study

Plasmid name	Characteristics	Reference/source
pBad-comR-$ST_{C\text{-}ter}$	pBADhisA derivative encoding $ComR_{HSISS4}$ fused to a C-terminal StreptagII	[1]
pBad-covR-$ST_{N\text{-}ter}$	pBAD plasmid encoding $CovR_{HSISS4}$ fused to a N-terminal StreptagII	[2]
pBad-pepF-$ST_{N\text{-}ter}$	pBAD-covR-$ST_{N\text{-}ter}$ derivative encoding *pepF*$_{HSISS4}$ fused to a N-terminal StreptagII	This work
pGhostCre	Thermosensitive replication origin vector in *S. salivarius*, encoding the Cre recombinase; *erm*	[3]
pGIUD0855erm	pUC18 derivative containing the *erm* gene	[3]
pJUDspecmut1-gfp+ter	Terminator associated-*gfp+* ORF cloned in pJUDspecmut1	[1]
pJIMcat	pJIM4900 derivative with a *cat* cassette	[1]

References

1. Mignolet J, Fontaine L, Sass A, Nannan C, Mahillon J, Coenye T et al. Circuitry rewiring directly couples competence to predation in the gut dweller *Streptococcus salivarius*. Cell Rep. 2018 Feb 13; 22(7):1627-38.

2. Knoops A, Vande Capelle F, Fontaine L, Verhaegen M, Mignolet J, Goffin P et al. The CovRS environmental sensor directly controls the ComRS signaling system to orchestrate competence bimodality in salivarius streptococci. mBio. 2022 Jan 4; e0312521.

3. Fontaine L, Dandoy D, Boutry C, Delplace B, de Frahan MH, Fremaux C et al. Development of a versatile procedure based on natural transformation for marker-free targeted genetic modification in *Streptococcus thermophilus*. Appl Environ Microbiol. 2010 Dec; 76(23):7870-7.
