# Supplementary material for: Competence shut-off by intracellular pheromone degradation in salivarius streptococci
Source: PLoS Genet. 2022 May 25;18(5):e1010198. doi: 10.1371/journal.pgen.1010198 (PMC9173638; doi:10.1371/journal.pgen.1010198)
Supplement: S3 Table — (PDF) [file pgen.1010198.s013.pdf]

Table S3. List of oligonucleotides used in this study

Name	Sequence (5' to 3')
**1022SA**	TTATAAAAATTTAATGAATATTATTCCGGTC
**1110SA**	TAAGGAAGATAAATCCCATAAGG
**AK72**	GCAAAAATGATAGAGTTACGTG
**AK73**	TAATGATGAAATCTGTCCAAAAATG
**AK74**	CATTTTTGGACAGATTTCATCATTATAAGGAAGATAAATCCCATAAGG
**AK75**	GGGGAATTTTGTTATAATGAAAGCTTCACGTTACTAAAGGGAATGTA
**AK76**	GCTTTCATTATAACAAAATTCCC
**AK77**	CAAATCATTCAAGCCAAATC
**AK78**	GGCTCAAATGTTTTATAGAATC
**AK79**	GATTTGGAGGAATACTTTATGC
**AK80**	GGCATAAAGTATTCCTCCAAATCTAAGGAAGATAAATCCCATAAGG
**AK81**	CCTAAAAGACCAAAGGAGTAATTTATATTCACGTTACTAAAGGGAATGTA
**AK82**	TATAAATTACTCCTTTGGTCTTTTAG
**AK83**	GACTATTTTGGTTTTAGAGTTAG
**AK88**	CTTATGGGATTTATCTTCCTTATAAATAATAAAAAAGCCGGATTAATAAT
**AK91**	CCTTATGGGATTTATCTTCCTTACTATTAGGTATATTCCATGTG
**AK106**	CTTTTGAAAAATTGAGAGGTATAAATCAACTGCAGAAAATTACAATAAGG
**AK211**	CATACAGTCTTTGGTCAATTG
**AK212**	CCTTATGGGATTTATCTTCCTTAGATGTCTAATTAATAAACATGGG
**AK213**	TACATTCCCTTTAGTAACGTGAATATTTCTTACACTCATTCTGTC
**AK214**	TGATACCGTCTACTTGTTTG
**AK262**	CATTTTGACTGCTATCTTTG
**AK264**	TTAAGTAGGAGTGCTTAATTC
**AK265**	AAATGAGTTATCCTTTCTCC
**AK266**	TTAAAATATAGATAAATATCATTTGATTAGTATTTGTTCACGTTACTAAAGGGAATGTA
**AK316**	CTTTTGAAAAATTGAGAGGTATAAATCAACTATTTTTAATGCTTATGTGGG
**AK317**	GTAAGCAAAAAGTTTCCAAATTTCATAACTCTCTCCTTCGTGCAC
**AK325**	CTATTGTTCTGTTAAAAATGCATTAAC
**AK330**	CCTTATGGGATTTATCTTCCTTAGTATAGTAACTTCTTTTTAAGAAAG
**AK331**	GTTTATACTCGTTCATATAATCAAATTGGAATGTCAGACAATCGTACTC
**AK338**	TTTTTACCTCCTTATTTTTTTTCTAG
**AK353**	GTTTGAATTAAGCACTCCTACTTAACTTTTGAAAAATTGAGAGG
**AK356**	CTTTTGAAAAATTGAGAGGTATAAATCAAGAAAAGAGATCAAATGGACTG
**AK357**	GTATGTAAGCAAAAAGTTTCCAAATTTCATAATATTCTCCTTAATTGTGTC
**AK384**	CTTCTAAATGAGTACGATTGTCTGAACCAGCAGCACCTTTCTCG
**AK385**	GAAAAAGGTGTTCACCTTTCTTAAGAATTCTAGAAGCTTGGC
**AK386**	CGAGAAAGGTGCTGCTGGTTCAGACAATCGTACTCATTTAGAAG
**AK387**	GCCAAGCTTCTAGAATTCTTAAGAAAGGTGAACACCTTTTTC
**AK458**	GTAGCAACACTCTTGTTTAAAG
**AK459**	GTGGCTGAATTATCAAAATAAATC
**AK472**	AAGTTTAAATAAGGCTAGTCCGTTATCAACTTG
**AK473**	TATAGTTATTATACCAGGGGGAC
**AK475**	CGGACTAGCCTTATTTAAAC
**AK476**	GTCCCCCTGGTATAATAACTATA
**AK487**	CTATTAAATTCTTTTATAGCTTTACC

**AK488**	TTAAGACGATAGGCACTACTG
**AK489**	GTTTTAAATAAAGTTTGGATAGAGG
**AK490**	TGACTATTTTTCTTACTTGTCTG
**AK495**	GACTAATCCTCTATCCAAACTTTATTTAAAACTTCACGTTACTAAAGGGAATGTA
**AK496**	CCTTTTTTTAGACAATAAATGCG
**AK497**	GAAAAGTATGAAGCAGAGTAG
**AK498**	CTTTTCTTTATGAGCCATTTAAC
**AK499**	ATCGTAAAGAAAAGCTAAACG
**AK516**	GTACAGACTACTCTGCTTCATACTTTTCACTTATAGGGGTAACACTTAAAAAAG
**AK517**	GCTTTTTTAGTTAAATGGCTCATAAAGAAAAGTTCACGTTACTAAAGGGAATGTA
**AK521**	AACGTTGACTGTAACATACAAC
**AK522**	TACATTCCCTTTAGTAACGTGAATTCTTCAAGGAAAATCCCC
**AK523**	CCTTATGGGATTTATCTTCCTTAATGTTAATTGCAAGAAATCAGAAAG
**AK524**	CAATCCAACGTTCCGTAAAG
**AK531**	CTTTTGAAAAATTGAGAGGTATAAATCAAGAAAAATCCAAAGTGGATGAG
**AK532**	GTAAGCAAAAAGTTTCCAAATTTCATAATATTCTCCTTAATTGTGTC
**AK563**	AGTACAGTCGGCATTATCTCATATTATAAAAATTTAATGAATATTATTCCGG
**AK564**	TATGAGATAATGCCGACTGTACT
**AK577**	CTACCAGTAGTGCCTATCGTCTTAAGAAAAGAGATCAAATGGACTG
**AK580**	CCTTATGGGATTTATCTTCCTTATAACTTCTTTTTAAGAAAGGTGAAC
**AK657**	CCTTATGGGATTTATCTTCCTTAAAAATAAGGCAAGATAGTGATAAG
**AK658**	GACCGGAATAATATTCATTAAATTTTTATAAATGGGTTGTCTTTAATAAGGAGTC
**AK677**	GATATTTTGACATGGTGACTCCTTATTACAAAATAAAACTCCTTTTAACTG
**AK678**	CATAACTAACAGTTAAAAGGAGTTTTATTTTGTAATAAGGAGTCACCATGTC
**AK687**	CATTTATGTCACCACCATTTCTACAAGAGACACTCCTTTATTTCG
**AK688**	CGAAATAAAGGAGTGTCTCTTGTAGAAATGGTGGTGACATAAATG
**AK689**	AGACAACCTATAAAATAAGGCAAGATAGTGATAAG
**AK690**	CCTTATTTTATAGGTTGTCTTTAATAAGGAGTC
**AK691**	CAACCTGCATAATAAGGCAAGATAGTGATAAGTAG
**AK692**	TTGCCTTATTATGCAGGTTGTCTTTAATAAG
**AK693**	ACAACCTGCCCAATAAGGCAAGATAGTGATAAGTAG
**AK694**	CCTTATTGGGCAGGTTGTCTTTAATAAGGAGTCACCATG
**AK695**	AAGACAACCTATATAATAAGGCAAGATAGTGATAAGTAG
**AK696**	CTTATTATATAGGTTGTCTTTAATAAGGAGTC
**AK697**	AAAGACAACCTATCCAATAAGGCAAGATAGTGATAAGTAG
**AK698**	TTATTGGATAGGTTGTCTTTAATAAGGAGTC
**Cy3_F_PcomX**	Cy3-TTTTATAGTGACATATATGTCGCTATTTTA
**Dn.comX 578**	TACTGGCTTCTTAGTATCCT
**Dn.Rv.lox71**	TTCACGTTACTAAAGGGAATGTA
**DR.THR**	AAGGAGAAAATTATGTACAC
**DR_SER**	TTGGATAAGGTCTTGACTTC
**DR_THR**	AAGGAGAAAATTATGTACAC
**LL61**	CATGGGAGTTCTCATCCTATAAATCAACCTCTTTGAACATA
**luxAB.ATG**	ATGAAATTTGGAAACTTTTTGC
**ML42**	CCTTATGGGATTTATCTTCCTTATCAACCTCCTATTAATAGATATAATTTTTG
**ML43**	TACATTCCCTTTAGTAACGTGAATCCTTAATATAGAACCTTGACTG
**ML45**	TAAGGAAGATAAATCCCATAAGGTAAACATAAACATGCAAGGAG
**ML46**	TTCACGTTACTAAAGGGAATGTAGAAAAGGCAATTGCGTTACC

**PH497**	TTGCTTACAGTTGCTATGGT
**PH499**	TCATCACAATGGTCACATCT
**R_PcomX**	TAAAATAGCGACATATATGTCACTATAAAA
**R_spec**	ATAGGATGAGAACTCCCATG
**UF.THR**	TGTCAAAGGATTAGGAAAAC
**UF_SER**	CAAGATTAACCATGACCTTC
**UF_THR**	TGTCAAAGGATTAGGAAAAC
**Up.comX 557**	CAAGATGAGATTTCCCATGA
**Up.fw.lox66**	TAAGGAAGATAAATCCCATAAGG
**UR.THR**	TTGATTTATACCTCTCAATTT
