# Supplementary material for: Competence shut-off by intracellular pheromone degradation in salivarius streptococci
Source: PLoS Genet. 2022 May 25;18(5):e1010198. doi: 10.1371/journal.pgen.1010198 (PMC9173638; doi:10.1371/journal.pgen.1010198)
Supplement: S4 Table — (PDF) [file pgen.1010198.s014.pdf]

Table S4. List of PCR fragments, synthetic DNA constructs, and EMSA probes

Strain/ Plasmid	PCR fragment	Template DNA	Primer 1	Primer 2
AK0043	Up HR of $tRNA_{thr}$	HSISS4	UF_THR	UR_THR
	P_{comS}-*opt-gfp*$^{+}$, *comS* promoter with 5'UTR optimized sequence fused to codon-optimized *gfp*$^{+}$	P_{comS}-*gfp*$^{+}$ (gBlocks DNA[a])	AK106	AK88
	Spc fused-Dw HR of $tRNA_{thr}$	Strain AK0001[b]	Up.fw.lox66	DR_THR
AK0044	P_{xyl2}-*comR* overexpression system fused to a chloramphenicol resistance cassette at the $tRNA_{ser}$ locus	Strain AK0004[b]	UF_SER	DR_SER
AK0045	Up HR of *tnpII*	HSISS4	AK262	AK266
	cat resistance cassette	pJIMcat	Up.fw.lox66	Dn.rv.lox71
	pepB	HSISS4	AK330	AK331
	P_{32}-*opt*, constitutive 5'UTR optimized P_{32} promoter	P_{32}-*opt-gfp*$^{+}$ (gBlocks DNA[a])	AK338	AK353
	Dw HR of *tnpII*	HSISS4	AK264	AK265
AK0046	Up HR of *SUC*	HSISS4	AK496	AK497
	P_{xyl2} xylose-inducible promoter fused to *comR* and to a chloramphenicol cassette resistance *cat*	Strain AK0004[b]	AK516	AK517
	Dw HR of *SUC*	HSISS4	AK498	AK499
AK0047	Up HR of *GOR* locus fused to the *erm* resistance, a terminator and the dcas9 handle	Strain AK0036[b]	AK458	AK472
	dCas9 handle together with a guide targeting the promoter of *pepF* (P_{pepF})	AK482	AK475	AK476
	P_{3} promoter together with Dw HR of *GOR* locus	Strain AK0036[b]	AK473	AK459
AK0048	Up HR of *pepO*	HSISS4	AK72	AK73
	cat resistance cassette	pJIMcat	AK74	AK75
	Dw HR of *pepO*	HSISS4	AK76	AK77
AK0049	Up HR of *pepP*	HSISS4	AK78	AK79
	cat resistance cassette	pJIMcat	AK80	AK81
	Dw HR of *pepP*	HSISS4	AK82	AK83
AK0050	Up HR of *pepQ*	HSISS4	AK211	AK212
	cat resistance cassette	pJIMcat	Up.fw.lox66	Dn.rv.lox71
	Dw HR of *pepQ*	HSISS4	AK213	AK214
AK0051	Up HR of $tRNA_{thr}$	HSISS4	UF.THR	UR.THR
	Promoter of *coiA* P_{coiA}	HSISS4	AK316	AK317

	luxAB genes together with *spc* resistance cassette and Dw HR of *tRNA*$_{thr}$	Strain AK0001[b]	luxAB.ATG	DR.THR
AK0052	Up HR of *tRNA*$_{thr}$	HSISS4	UF.THR	UR.THR
	Promoter of *pepF* (P_{pepF})	HSISS4	AK531	AK532
	luxAB genes together with *spc* resistance cassette and Dw HR of *tRNA*$_{thr}$	Strain AK0001[b]	luxAB.ATG	DR.THR
AK0053	Up HR of *tRNA*$_{thr}$	HSISS4	UF.THR	UR.THR
	Promoter of *coiA* P_{coiA} fused to *coiA* and the *pepF* promoter (P_{coiA}-*coiA*-P_{pepF})	HSISS4	AK356	AK357
	luxAB genes together with *spc* resistance cassette and Dw HR of *tRNA*$_{thr}$	Strain AK0001[b]	luxAB.ATG	DR.THR
AK0054	Up HR of *comX*	HSISS4	AK563	ML42
	Erm resistance cassette	pGIUD0855ery	ML45	ML46
	Dw HR of *comX*	HSISS4	ML43	AK564
AK0055	Up HR of P_{coiA}	HSISS4	AK521	AK522
	spc resistance cassette	pJUDspecmut1-gfp+ter	Dn.Rv.lox71	Up.Fw.lox66
	Dw HR of P_{coiA}	HSISS4	AK523	AK524
AK0056	Up HR of *FBA* locus	HSISS4	AK487	AK488
	P_{coiA}-*coiA*-P_{pepF}-*pepF* operon	HSISS4	AK577	AK580
	Cat resistance cassette	pJIMcat	Up.Fw.lox66	AK495
	Dw HR of *FBA* locus		AK489	AK490
AK0057, AK0058, AK0059, AK0060, AK0061, LL0053	Up HR of *comRS* locus	HSISS4	AK325	AK657
	cat-oroP cassette	*cat-oroP* cassette[d]	1110SA	1022SA
	Dw HR of *comRS locus*	HSISS4	AK658	PH499
AK0057	Up HR of *comRS* locus, without *comR*	HSISS4	PH497	AK687
	Dw HR of *comS*	HSISS4	AK688	PH499
AK0058	Up HR of *comRS* locus, without *comR*	HSISS4	PH497	AK687
	P_{comS}-*comS,* introducing F4Y mutation	HSISS4	AK688	AK691
	Dw HR of *comS*, introducing F4Y mutation	HSISS4	AK692	PH499
AK0059	Up HR of *comRS* locus, without *comR*	HSISS4	PH497	AK687
	P_{comS}-*comS* introducing F4W mutation	HSISS4	AK688	AK693
	Dw HR of *comS*, introducing F4W mutation	HSISS4	AK694	PH499
AK0060	Up HR of *comRS* locus, without *comR*	HSISS4	PH497	AK687
	P_{comS}-*comS* introducing A5I mutation	HSISS4	AK688	AK689

	Dw HR of *comS*, introducing A5I mutation	HSISS4	AK690	PH499
AK0061	Up HR of *comRS* locus, without *comR*	HSISS4	PH497	AK687
	P_{comS}-*comS* introducing F4YA5I mutation	HSISS4	AK688	AK695
	Dw HR of *comS*, introducing F4YA5I mutation	HSISS4	AK696	PH499
AK0062	Up HR of *comRS* locus, without *comR*	HSISS4	PH497	AK687
	P_{comS}-*comS* introducing F4YA5I mutation	HSISS4	AK688	AK697
	Dw HR of *comS*, introducing F4YA5I mutation	HSISS4	AK698	PH499
AK0063	Up and Dw HR of *comRS* locus fused to *cat*	Strain JM1002[c]	PH497	PH499
AK0064	Up and Dw HR of *comX* locus fused to *cat*	Strain JM1013[c]	Up.comX 557	Dn.comX 578
LL0052	Up HR of $tRNA_{thr}$ locus fused to P_{comS}-*luxAB*	JM1019	UF_THR	AK91
	spc, spectinomycin resistance cassette	pJUDspecmut1-gfp$_{+}$ter	Up.fw.lox66	R_spec
	Dw HR of $tRNA_{thr}$ locus	HSISS4	LL61	DR_THR
LL0053	Up HR of *comS*	HSISS4	PH497	AK677
	Dw HR of *comS*	HSISS4	AK678	PH499
pBad-pepF-ST_{N-ter}	pBAD plasmid backbone	pBAD-covR-ST_{Nter}	AK384	AK385
	$pepF_{HSISS4}$	HSISS4	AK386	AK387

**EMSA probes**	**Template**	**Primer 1**	**Primer 2**
HSISS4 promoter of *comX* fused to Cy3	HSISS4	Cy3_F_PcomX	R_PcomX

[a] gBlocks DNA are synthetic dsDNA provided by Integrated DNA Technologies (IDT)

[b] [1]

[c] [2]

[d] [3]

References

1. Knoops A, Vande Capelle F, Fontaine L, Verhaegen M, Mignolet J, Goffin P et al. The CovRS environmental sensor directly controls the ComRS signaling system to orchestrate competence bimodality in salivarius streptococci. mBio. 2022 Jan 4; e0312521.

2. Mignolet J, Fontaine L, Sass A, Nannan C, Mahillon J, Coenye T et al. Circuitry rewiring directly couples competence to predation in the gut dweller *Streptococcus salivarius*. Cell Rep. 2018 Feb 13; 22(7):1627-38.

3. Dorrazehi GM, Worms S, Chirakadavil JB, Mignolet J, Hols P, et al. Building scarless gene libraries in the chromosome of bacteria. In: Iranzo O, Roque A, editors. Peptide and Protein Engineering, Springer Protocols Handbooks. New York, NY: Humana; 2020. pp. 189-211.
