# Supplementary material for: Competence shut-off by intracellular pheromone degradation in salivarius streptococci
Source: PLoS Genet. 2022 May 25;18(5):e1010198. doi: 10.1371/journal.pgen.1010198 (PMC9173638; doi:10.1371/journal.pgen.1010198)
Supplement: S1 Appendix — (PDF) [file pgen.1010198.s015.pdf]

Appendix S1. Mathematical model of the ComRS system in *Streptococcus salivarius*

The ComRS signalization pathway has been extensively studied in salivarius streptococci but remains partially understood. In a previous paper, we determined experimentally the key processes involved in competence bimodality *i.e.* the heterogeneous activation of competence restricted to a subpopulation [1]. We showed that ComR abundance is central to bimodality and highlighted the essentiality of a functional positive feedback loop for bimodal activation. Unexpectedly, the system did not activate upon the pheromone *comS* overexpression at ComR native abundance while activating at low synthetic XIP addition (sXIP). To understand such counter-intuitive mechanisms, we aimed to build a deterministic model describing the ComRS system in salivarius streptococci. Of note, because we characterized thoroughly the ComRS system in *Streptococcus salivarius* at the single cell level in a previous paper [1], most of the experimental data used to parametrize and validate the model will come from this source.

1. Materials and experimental data

Strains, primers, and methods used for generating experimental data used for the parametrization and validation of the model are described in the Materials and Methods section of the main text or in [1]. The experimental data used for the modeling are available in the S1 Dataset. The Matlab code is available as S2 Appendix. Those two files can be run together using Matlab and take approximately 45 min to complete.

2. Key processes in ComRS signalization

The ComRS activation has been shown to mostly rely on ComR abundance [1–3]. Also, experimental data have underlined the necessity of a functional positive feedback loop for bimodal activation [1]. In addition, results obtained experimentally in *S. salivarius* showed a low sensitivity of the system regarding ComS (peptide pheromone signal), PptAB (peptide exporter), and Opp (peptide importer) abundance [1]. While those three players are crucial to maintain the functionality of the positive loop, it seems they do not stand as bottlenecks for activation. For this reason, we chose to build a model neglecting the export-processing-reimportation of the peptide by assuming the peptide maturation as a single process. In consequence, we will consider that the mature form of ComS (XIP) is equal to ComS and the ComS term will only be used, except if else stated.
The characterization of the formation of the ComRS complex in *Streptococcus thermophilus* showed a high affinity between ComR and ComS [4,5].We therefore simulated in the model that the binding between the two players is irreversible, even though the complex can be further degraded after binding.
Finally, because competence induces a cell-division arrest [6], we chose to neglect the dilution of the different players through cellular growth. Since we model the system during, before, and after competence induction, we will have an effect of this dilution. However, we decided to include it in the degradation rate of the different players.

In summary, the different assumptions are:
1. Exportation, maturation, and reimportation of the peptide is a one-step process (XIP=ComS).

2. ComR and ComS binding is irreversible.
3. Dilution of the players through growth is included in the degradation rate.

3. Mathematical formulation

The model was built using the four main players of the system: ComS (the pheromone peptide), ComR (intracellular receptor), the ComRS complex (formed by two ComS and two ComR) and ComX (alternative sigma factor activating the late phase of competence). Since we had few quantitative data on the ComR control through CovRS, we decided to model the basal abundance of ComR fluctuating over time according to luciferase expression data, independently of any other player. We based mainly our model on two other models described for *S. thermophilus* [2] and *Streptococcus mutans* [7]. Ordinary differential equations (ODEs) of the model were written as follow:

$$\frac{dComR}{dt} = b_R(t) - d_R.ComR(t) - k_{RS}\big(ComR(t).ComS(t)\big)^n$$

$$\frac{dComS}{dt} = max_S\left(\left(\frac{ComRS(t)}{ComRS(t) + K_S}\right) + b_S\right) - d_S.ComS(t) - k_{RS}\big(ComR(t).ComS(t)\big)^n$$

$$\frac{dComRS}{dt} = \frac{1}{2}k_{RS}\big(ComR(t).ComS(t)\big)^n - d_{RS}.ComRS(t)$$

$$\frac{dComX}{dt} = max_X\left(\left(\frac{ComRS(t)}{ComRS(t) + K_X}\right) + b_X\right) - d_X.ComX(t)$$

We modeled the fluctuation in ComR abundance as the result of basal rate production over time, subtracted by its degradation rate and its inclusion into the ComRS complex (through a non-linear mass action law modeled by a Hill equation). ComS amount was modeled as the result of a basal constant production plus the transcriptional activation by ComRS (through a classical Michaelis-Menten equation involving saturation). Similarly to ComR, the negative terms were associated to degradation and inclusion into the ComRS complex. Regarding ComRS, we described its production as the half quantity (since dimeric) of the non-linear association between ComS and ComR minus its degradation. Finally, ComX was computed as the result of a basal rate of production, plus the transcriptional activation through ComRS (Michaelis-Menten kinetics) and minus its degradation. The different parameters, their meaning, corresponding values, and origin are described in the next section and in the Table 1 at the end of this appendix.

3.1. Model calibration

With the mathematic model described above, we ended up with a total of 12 parameters. Fortunately, a previous modeling of the system in *S. thermophilus* [2] and experimental data obtained during this work helped us to determine the range of values for each parameter. Table 1 at the end of this appendix provides an overview of all the parameters of the model.

3.1.1. Literature-based parametrization

Previous deterministic modeling of the close relative *S. thermophilus* species helped us to determine mostly the degradation parameters: ComR, ComRS and ComX degradation rate (d_R, d_{RS}, d_X).
Regarding the degradation rate of ComS (d_S), we chose not to use the previously reported parameter from the *S. thermophilus* model because of contradictory experimental results obtained in *S. salivarius* [1].
K_S and K_X represent the affinity of the ComRS complex for P_{comS} and P_{comX}, respectively. Because ComR-binding box at the DNA level and its interaction with the ComRS complex are highly conserved between *S. thermophilus* and *S. salivarius* [3,5], we chose to keep an upscaled version of the parameters used in our previous *S. thermophilus* model [2]. Since the amount of ComRS molecules produced in our model in comparison to the *S. thermophilus* model is 322 times higher, we upscaled K_S and K_X by this factor.
We used a Hill coefficient for ComR binding to ComS of two due to the dimeric nature of the binding, which involves two molecules of ComS and two molecules of ComR [4].

3.1.2. Experimental parametrization

Of note, all the experimental luciferase and growth data used in this section (P_{comS}/P_{comR}/P_{comX}-luxAB) and showed in Dataset 1 were shifted (delayed or brought forward) in time in order to have similar growth curves, allowing us to compute and compare different reporter strains together.

ComR basal rate ($b_R(t)$)

Since competence is a transient state that is mainly depending on ComR abundance, we suspected that *comR* expression is also transient. Transcriptional fusion between the *comR* promoter and a luciferase gene (P_{comR}-*luxAB*) described in [1] allowed us to monitor the activation of the promoter over time. Indeed, we observed a strong activation at the early stage of exponential phase, with a sharp decrease upon entry into stationary phase (Fig. 1A).

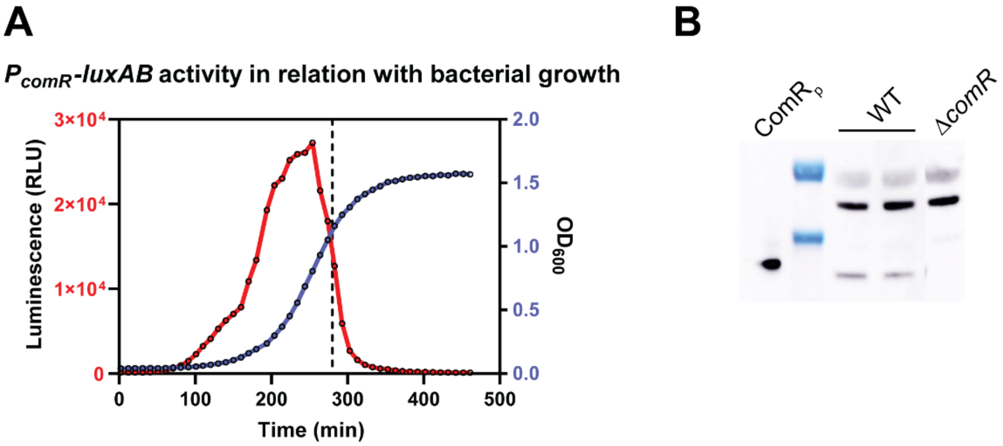

Figure 1. A. P_{comR}-*luxAB* activity in comparison to growth. Red and blue lines denote experimental mean values (biological triplicates) of luminescence measurement and optical density at 600nm, respectively. The vertical black dashed line shows the time of sampling for Immunoblotting. **B.** ComR semi-quantification through western blot. The ComR amount in WT was quantified thanks to the recombinant purified $ComR_p$ used as a standard. Number of ComR molecules on the blot were then divided by the number of cells present in the original extract, assuming a complete lysis of the cells.

In parallel, semi-quantification through western blotting experiments on the WT strain gave us an estimate of the amount of ComR (Fig. 1B) at a given time (280 min, dashed line on Fig. 1A). Combining those two data, we were able to generate an approximation of ComR production over time. We used the same method as previously described [2] for processing luminescence measurement into protein amount production per cell. The only change that was brought to this method was the choice of the luminescence to protein amount factor based on the western blot semi-quantification (Fig. 1B). Of note, this quantification relies on the assumption of a complete lysis of the cell population before blotting. Because this hypothesis is certainly not accurate, this quantification should be taken as a model parametrization and certainly not as an absolute real value.

Maximum synthesis rate of comS (max_s)

We used the same approach for modeling the maximum synthesis of ComS than for the basal synthesis of ComR. In short, we used absorbance data describing growth and luciferase expression of a P_{comS}-*luxAB* strain to model the growth curve and growth rate together with the activity of the P_{comS} promoter upon competence activation. For activating the competence, we overexpressed *comR* thanks to a xylose-inducible promoter fusion (P_{xyl2}-*comR*) present in a neutral locus of the strain by adding xylose at a final concentration of 0.5%.

Since we had no quantification of ComS or XIP available, we used the same *RLUmol* factor obtained for P_{comR}-*luxAB*. Because we know that transcription efficiency can vary from one fusion to another and that translation properties of a mRNA will influence translation efficiency, we compared the total amount of ComS produced during our modeling with existing data in *S. mutans*

[8] for validation (see below). Finally, we calculated the maximum production rate and used it for max_s.

Basal rate of comS (b_s)

As previously described, we first computed the growth curve and growth rate for a non-activating competence population (0% xylose added) and then modeled the ComS synthesis rate thanks to the P_{comR} *RLUmol* factor previously calculated. We then compared the maximum synthesis rate obtained for the non-activating vs activating competence population to obtain the fold increase in P_{comS} expression upon competence activation. We then used this fold increase (384-fold) to compute the basal rate of ComS synthesis (b_s) such as:

$$\frac{1 + b_s}{b_s} = fold\ increase$$

Where 1 represents the maximum P_{comS} activation through the ComRS complex and (1+b_s) is thus the maximum rate synthesis (basal rate and ComRS induction).

Basal rate of comX (b_x)

We used exactly the same procedure for b_x as we used for b_s. The max_x parameter used as well as the *RLUmol* factor were chosen as described in the next free parameter calibration section. We used the same equations as described for b_s computation, and calculated a P_{comX} fold-change between competence activated cells and non-competent activated cells of 95.

3.1.3. Free parameters calibration

To calibrate our model with the three *comS*-related free parameters left describing ComS activity (k_{rs}, max_s, d_s), we used microscopic data showing that *S. salivarius* cells do not activate *comX* at basal ComR amount (401±32,5 molecules/cell) while they nearly all (95%) activate *comX* at higher ComR amount (3507±713 molecules/cell representing a 8.5-fold increase) [1]. We used those two data (0% of cells activated vs ~100%) in a deterministic frame, considering the bacterial population as one entity being either ON (~100%) or OFF (0%). Because the three parameters (k_{rs}, max_s, d_s) influence ComS activity, we calibrated them together, computing the maximum number of ComX produced per simulation, using either low or high ComR values (Fig. 2). As we expected the system to be in an ON state (production of ComX) for high ComR values and in an OFF state (no production of ComX) for low ones, we used as output the factor $ComX_{ON/OFF}$:

$$ComX_{ON/OFF} = Max\left(ComX_{High\ ComR}(t)\right) - Max\left(ComX_{Low\ ComR}(t)\right)$$

and draw the output as surface values for different parameter values as shown in Fig. 2. High values of $ComX_{ON/OFF}$ are associated with permissive parameters for bimodality (*i.e.* ComX not activated at basal ComR amount and ComX activated at 8.5-fold basal ComR amount). Of note, higher k_{rs} values can generate a region where $ComX_{ON/OFF}$ has intermediate values meaning that the basal or 8.5-fold basal ComR system is not completely OFF or ON within the range of parameters under

consideration. For this reason, we will consider those parameter ranges as non-permissive for bimodality.

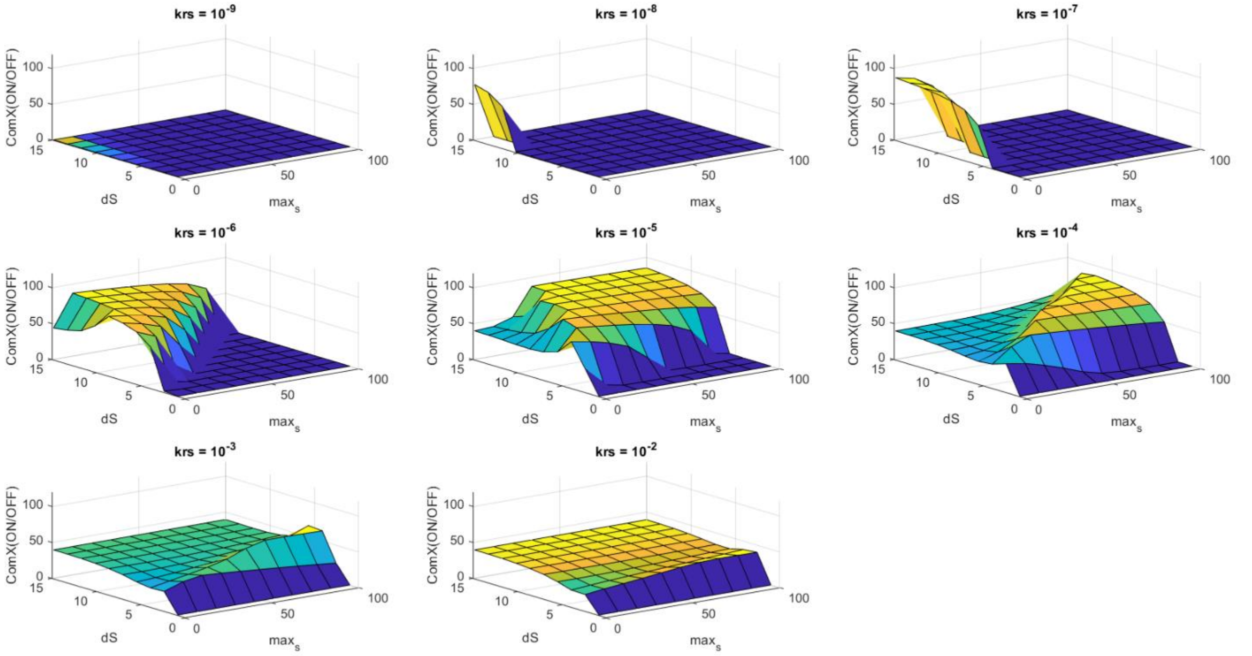

Figure 2. Surface plots showing the value of $\mathbf{ComX_{ON/OFF}}$ for different parameter values of $\boldsymbol{k_{rs}}$, $\boldsymbol{d_S}$ and $\boldsymbol{max_S}$. Results show that a bimodal state (meaning high $ComX_{ON/OFF}$ values) would only be possible for parameters values ranging from 10^{-8} to 10^{-4} for k_{rs}, and either high d_S rate for low k_{rs} or high max_S for higher k_{rs}.

Using parameter values permissive for bimodal activation (k_{rs} values of 10^{-8} to 10^{-4}, d_S of 5 to 15 min^{-1} and max_S production of 1 to 100 $mol.cell^{-1}.min^{-1}$), we were able to generate a non-linear solution resulting in ComRS activation only at 8.5 (or higher) ComR fold-change (Fig. 3). Dynamics of the system displayed on Fig. 3 showed that our model responds in a non-linear fashion and could produce bimodality. Indeed, we had a system turning ON for small increase in ComR basal expression, suggesting that small heterogeneity in the ComR distribution could produce different phenotypes. We used those results to tune the amount of ComX produced when competence was activated based on the amount of ComX boxes found in the genome of *S. salivarius* (~150, [3]). For this purpose, we set the max_X parameter at 40 $mol.cell^{-1}.min^{-1}$, resulting in a maximum concentration of 150 $mol.cell^{-1}$.

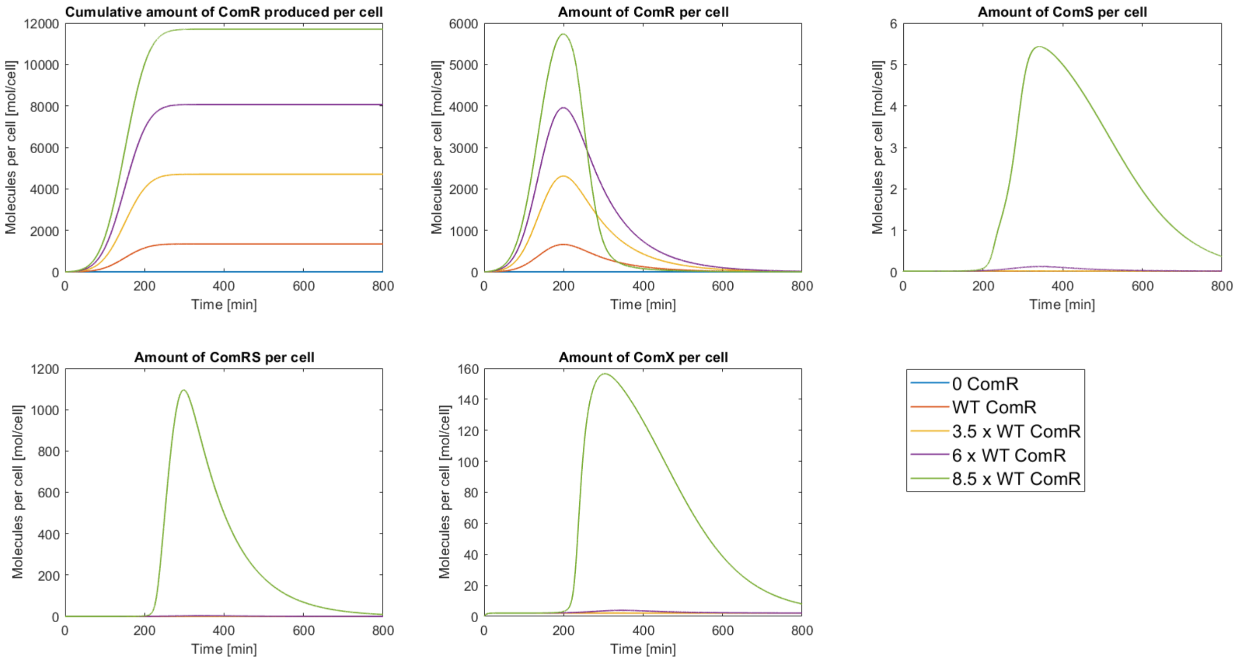

Figure 3. Simulation of the four variables of the system (ComR, ComS, ComRS and ComX) over time depending on the ComR concentration. First chart depicts cumulative amount of ComR for the 5 simulations, neglecting ComR degradation and incorporation into the ComRS complex. Second, third, fourth and fifth chart show the evolution over time of the different variables (ComR, ComS, ComRS, and ComX, respectively) regarding the amount of basal ComR computed in the system.

While our system showed bimodal properties, the free pheromone ComS produced in the cell was extremely low (max. ~5 mol/cell; Fig. 3, panel 3) and not in agreement with previous reports [8,9]. Because the parametrization of max_S, k_{rs} and d_S had shown that a low activity of ComS was necessary for bimodal activation (Fig. 2) and that experimental data suggested high k_{rs} and max_S values, we tried to conciliate those observations by re-examining the degradation rate. Various types of negative regulation of the activity of the communication pheromone have been described in streptococci: peptide interference [10], transcriptional inactivation [11,12], peptidase degradation [13,14]. Other mechanisms such as specific RNase activity have also been hypothesized [3], suggesting a high diversity of competence control mechanisms through peptide negative regulation. Therefore, we hypothesized the presence of an additional negative player of the system, which is degrading ComS. We aimed to model the presence of a constitutively expressed player (named *deg*) that would reduce the basal amount of ComS. In parallel, this new player had to be quickly saturated by ComS upon ComRS activation in order to allow high ComS concentration in competence activated cells. We modeled this *deg* saturating activity through a new Michaelis-Menten equation term in the ComS equation:

$$-k_{cat} \times deg \times \frac{ComS}{ComS + K_{deg}}$$

Where k_{cat} is the apparent constant rate of degradation of ComS through the negative regulator, *deg* is the constant *deg* concentration due to constitutive expression, and K_{deg} is the ComS concentration required for half max degradation of ComS.

We implemented this new term in our model and searched for permissive values for bimodal activation with parameters d_S = 0.01 min^{-1}, max_S = 1000 mol.cell^{-1}.min^{-1} and k_{rs}= 0.01 (d_S and k_{rs} values were chosen based on the previous simulation [2]. The max_S value was chosen to approach literature data [8], suggesting ComS production upon competence activation in the nanomolar to micromolar range. This value was also chosen in accordance with recent data published on the rate quantification of bacterial translation synthesis [15].

We used the same approach described before to generate surface plot showing bimodal landscape (data not shown) but using instead $ComS_{ON/OFF}$ in function of two parameters values: $k_{cat} \times deg$ and K_{deg}. $ComS_{ON/OFF}$ was used in order to have an idea of the maximum ComS values for activating cells. Because computation time were far too long for low K_{deg} values permissive for bimodal activation, we decided to keep low k_{rs} values (10^{-6} cell^3.mol^{-3}.min^{-1}). We realized that those two parameters (K_{deg} and k_{rs}) were responsible for the same effect on the model: increasing the ComS production necessary for activating the system. While low K_{deg} has much more biological significance than k_{rs} regarding our *in vitro* ComRS binding data, low K_{deg} was too difficult to compute in our model.

The parameter landscape regarding $ComS_{ON/OFF}$ (thus bimodality) showed that only some particular values were permissive for bimodality. Because low K_{deg} was necessary for bimodality and low $k_{cat} \times deg$ could provide a fair number of ComS molecules produced when competence is activated, we hypothesized that the properties of the negative player would directly influence the bimodal behavior. While high ComS affinity for the degradation regulator could provide a bimodal activation through efficient low ComS degradation, fast saturation can account for the high ComS production in competence activating cells.

Then, we used this new set of equations to model competence over time in relation to ComR abundance. Regarding previous parametrization, we decided to use $k_{cat} \times deg$ = 140 mol.cell^{-1}.min^{-1} and K_{deg} = 1 mol.cell^{-1} for computations (Fig. 4). As expected, we could not see any competence activation for lower values than 8.5×ComR. However, unlike our previous model (showing low level of unbound ComS when competence was activated), we could reach about 60,000 molecules per cell of ComS in the activating state. Because this amount would correspond to a nanomolar range of concentration in the growth medium, we concluded that our degradation model described more accurately the features of the ComRS system.

Of note, the ComS amount at the late stage of growth in our model is still important (Fig. 4, panel 3), probably because we did not include a specific shut-off mechanism of the system. We designed our model with this typology because we were mainly interested in using the simplest model as possible describing bimodal activation and were lacking experimental data regarding shut-off mechanisms at this point of the research.

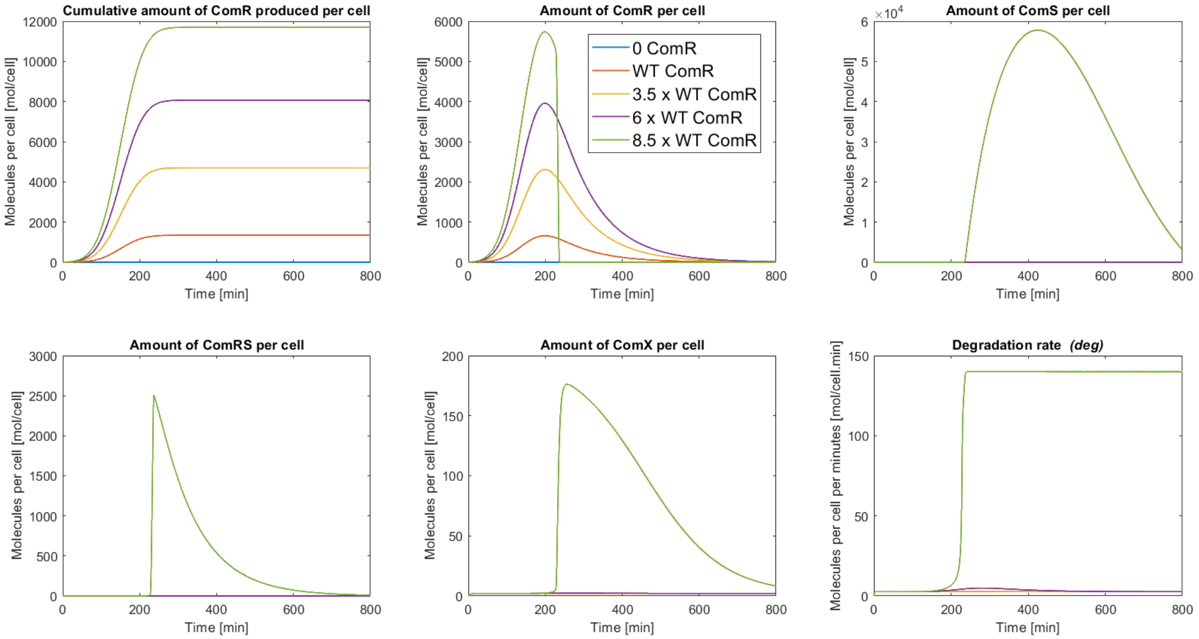

Figure 4. Modeling of the four variables of the system (ComR, ComS, ComRS and ComX) and the degradation rate of ComS over time depending on ComR concentration. First chart depicts cumulative amount of ComR for the 5 simulations, neglecting ComR degradation and incorporation into the ComRS complex. Second, third, fourth and fifth charts show the evolution over time of the different variables (ComR, ComS, ComRS, ComX and the degradation rate, respectively) regarding the amount of basal ComR computed in the system. Only the 8.5-times ComR increase triggers ComS, ComRS and ComX production and saturates fastly the degradation mechanism (panel 6).

3.2. Validation

We then sought to validate our model with a set of experimental data. To do this, we determined if our model could predict the percentage of cells activating competence in ComR overexpressing strains [1].

To this aim, we first determined the hypothetical maximum and minimum basal ComS production. For the minimum value, we used the fact that an 8.5-fold increase in WT ComR abundance is the threshold for the activation of the whole population. It means that all the cells in this situation, even the ones with the lowest basal ComS values, are able to trigger the system. We therefore determined that the minimum basal ComS production for the population was $b_S = 0.0026$, the basal value used in our simulation until now.

To compute the maximum basal ComS value, we used our microscopy data together with ComR semi-quantitative immunoblotting showing that a 1.27-fold ComR increase results in the first cells to activate competence.

We computed the basal ComS value necessary to obtain activation at a threshold of 1.27-fold ComR increase. We found a value of 0.03 for activating the system. We therefore hypothesized

that the ComS basal distribution within the population should range from 0.0026 to 0.03, which corresponds to a basal rate synthesis of ComS of 2.6 to 31 mol.cell^{-1}.min^{-1}. Of note, because experimental data obtained showed that ComR is homogeneously distributed in the population [1], we neglected the effect of small variations in ComR abundance from cell to cell. This approximation seems relevant, as weakly expressed genes such as *comS* show higher noise in expression [16]. Nevertheless, this assumption was not proven experimentally in *S. salivarius*, and still needs to be validated.

Recent theoretical and single molecule studies have shown that the random distribution of proteins can be modelized as a simple gamma function, knowing the mean and the dispersion of the protein amount in the population [7,17]. We considered the rough assumption that b_S is directly linked to the basal ComS concentration and we modeled this basal rate as a gamma function using single-cell data to have an idea of the dispersion and the mean of b_S. For this purpose, we analyzed the fluorescence of a P_{comS}-*gfp*$^+$ strain for competence non-activated cells (Fig. 5). Those cells were displaying only non-activated *comS* promoters, which correspond to basal ComS production. We first computed the Fano factor (F) where μ stands for the mean and σ^2 for the variance.

$$F = \frac{\sigma^2}{\mu}$$

Then, we used those values to generate a gamma distribution of the fluorescent intensity of the P_{comS}-*luxAB* population thanks to the equation [17]:

$$p(x) = \frac{x^{a-1}.\, e^{\frac{-x}{b}}}{\Gamma(a).\, b^a}$$

Where Γ is a gamma function, a is the inverse of the noise, and b is the Fano factor of the fluorescent distribution. With this equation, we generated the probability density function and the cumulative density function of the fluorescence intensity, as shown on the Fig. 5.

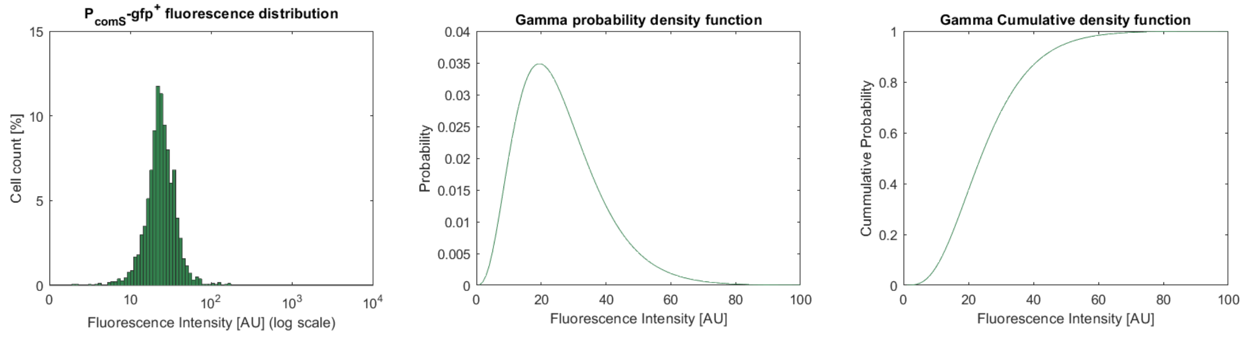

Figure 5. ComS distribution within the population. Left panel shows the experimental values obtained from a P_{comS}-*gfp*$^+$ strain for a competence non-activated population. Central and left panels show the associated gamma distribution.

Knowing that extreme values of fluorescence intensity would be associated to extreme values of predicted basal rates, we used the lower and upper quantile of the cumulative gamma function

(α=0.05) and computed their corresponding intensities (8.8 and 50, respectively). Using the maximum and minimum basal rate of ComS (b_{Smin}=0.0026 and b_{Smax}=0.03), we were able to scale the fluorescence intensity to the basal rate thanks to the equation:

$$I = b_S \times \frac{(I_{max} - I_{min})}{(b_{Smax} - b_{Smin})} \text{ [AU]}$$

We then computed the critical b_{Scrit} values for competence activation for several ComR concentrations and computed the associated I_{crit} factor thanks to the scaling equation (Fig. 6). Finally, we reported the I_{crit} value computed on the gamma cumulative density function (Fig. 5, panel 3) for every ComR fold increase to determine the percentage of cells with higher intensities than the I_{crit} value. This percentage represents cells with sufficient ComS basal activity to trigger the system. We were then able to generate a chart depicting the percentage of activated cells in the populations as a function of ComR abundance and compared it to experimental data obtained (Fig. 6).

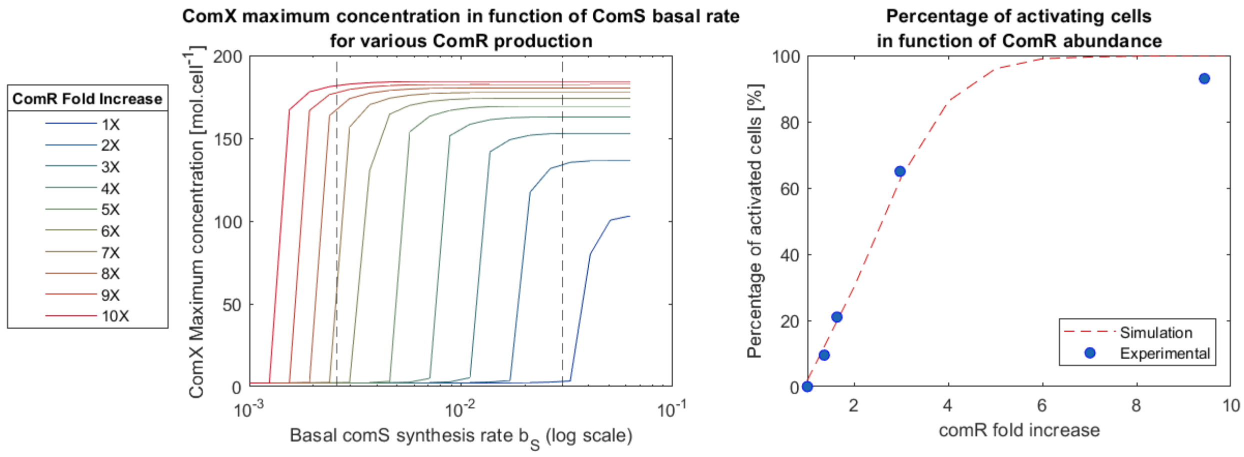

Figure 6. Model validation using percentage of competence-activated cells for various ComR concentrations. A. ComX maximum concentration computed in function of ComS basal rate for various ComR-fold increase and **B.** subsequent percentage of cells activating competence determined based on the inferred gamma distribution of b_s (Fig. 5, panel 3). Dashed lines in panel A depict threshold values for b_s (0.0026 and 0.03).

The comparison between experimental values and simulation showed that our model was able to describe quantitatively the bimodal competence activation through the ComRS system.

3.2.1. Competence bimodality and *comR* heterogeneity

Because our system has shown to be extremely ComR sensitive, we thought that although its distribution in the population is quite homogeneous (see experimental data on P_{comR}-*gfp*$^+$, P_{comR}-*comR::gfp*$^+$ and XIP population-wide dose in [1]) it was possible that small uneven ComR distribution in the population could account for bimodality. To validate this hypothesis, we challenged our model with a similar approach as performed with the *comS* distribution. First, we generated a gamma distribution fitted to the P_{comR}-*gfp*$^+$ data as previously described (Fig. 7).

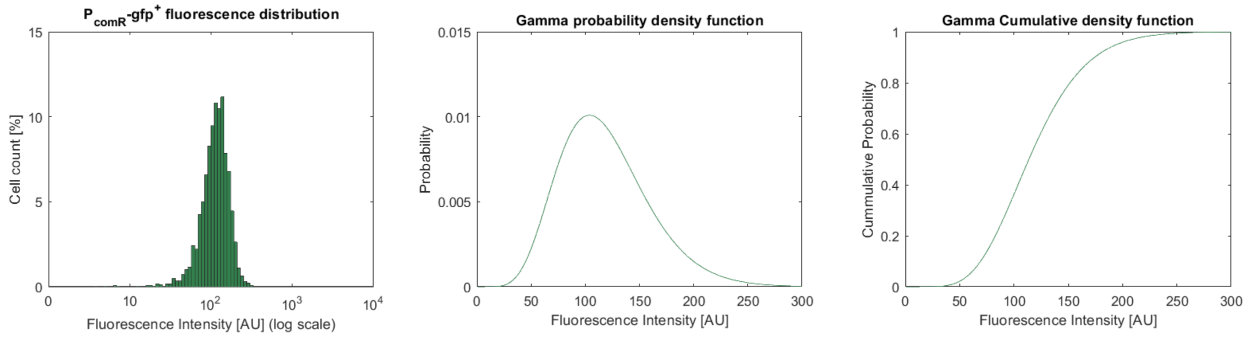

Figure 7. ComR distribution within *S. salivarius* population. Left panel shows the experimental values obtained from a P_{comR}-*gfp*$^{+}$ strain for a competence non-activated population. Central and left panel show the associated gamma distribution.

We first generated a 1.27-fold gamma distribution representing the ComR distribution in an overexpressing strain harboring a xylose-inducible promoter fused to *comR* and induced with 0.1% of xylose. To do this, we multiplied the mean and the standard deviation of our experimental data in the WT by 1.27 and generated a new fano factor and a noise parameter that we used to compute the gamma distribution of the 1.27-fold ComR amount strain. This strategy implies that the new distribution generated has the same mean over standard deviation ratio. We could reasonably make this assumption since P_{xyl2}-*gfp*$^{+}$ data obtained suggested homogeneous P_{xyl2} activation and would therefore only increase ComR amount without changing its distribution among the population [1].

We next used the 0.90 quantile value (corresponding to the 10% of cells activated measured experimentally) of the 1.27-fold distribution generated and defined it as the critical value for activation. This value corresponds to a 1.9-fold ComR change.

We then generated several ComR gamma distribution for different fold changes and computed the number of cells activated with the threshold (1.9-fold) defined. We then compared the computed percentages obtained with experimental values (Fig. 8).

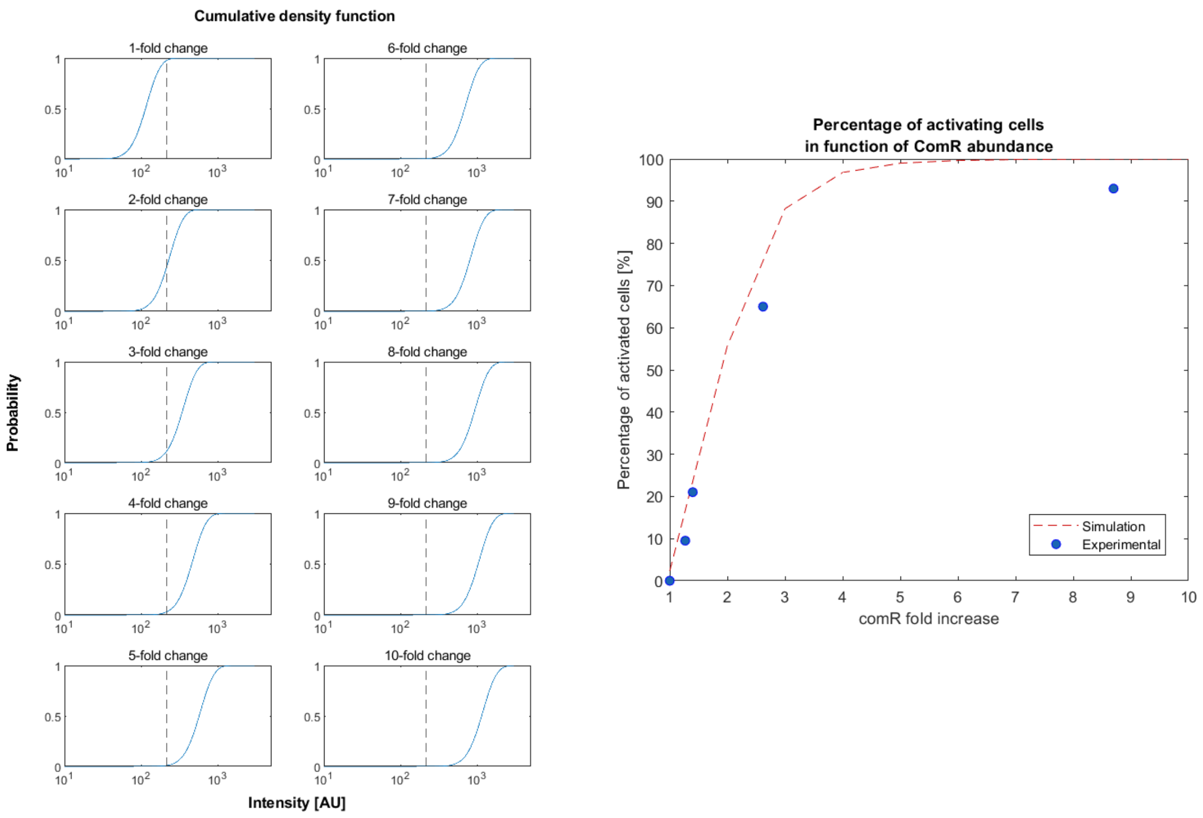

Figure 8. Validation of the model based on a ComR noisy expression as a source for bimodal competence. Left panel shows the computed gamma cumulative distribution based on experimental P$_{comR}$-*gfp*$^+$ data. The distribution is shifted to higher intensities values to simulate ComR overexpression. Dashed line denotes the ComR threshold value for competence activation previously computed. Right panel shows the simulated (red dashed line) vs the experimental percentage of activated cells (blue dots) for different values of ComR fold increase (regarding WT).

Fig. 8 shows clearly that ComR heterogeneity can be sufficient to explain bimodal activation of competence. Nevertheless, the parametrization used to obtain the fit presented in Fig. 8 must be considered for interpreting the validation. Indeed, the threshold determined based on experimental values is quite low (1.9-fold ComR increase). For triggering the system at this ComR concentration, high basal rate of ComS is needed (about 40 mol.cell^{-1}.min^{-1}) which is not in accordance with experimental data, suggesting low *comS* expression. Since the model suggests that both ComS and ComR heterogeneity could explain the bimodal activation of competence, it is most likely that heterogeneity on both partners is at the core of the stochastic process giving rise to bimodality.

3.3. sXIP addition vs *xip* overexpression prediction

Now that we had performed parametrization and validation, we could explore the model to ask biological relevant questions. We first investigated puzzling experimental results obtained regarding pheromone-based competence activation in *S. salivarius*. Particularly, the addition of nanomolar concentration of synthetic sXIP (sXIP) is able to trigger the system while the overexpression of the 12-amino acid maturated form of ComS (*xip*) fails to activate competence.

In order to evaluate if our model could explain such unexpected behavior, we modeled the addition of sXIP by a sharp and sudden increase in ComS concentration and modeled the *xip* overexpression by a constant ComS production over time. Of note, we will still consider in this section that XIP and ComS are equivalent.

Since recent investigations have shown the high processivity of the Opp transporter in *Bacillus subtilis* [18], we assumed that addition of XIP in the nanomolar concentration range would result in a fast uptake by the cells within a few minutes. Because our luciferase experimental data show an activation of the ComRS complex after 5 min upon sXIP addition, we modeled the total uptake taking place within 15 minutes and reaching a maximum rate after 5 minutes. In parallel, experimental sXIP titration with P_{comX}-gfp^{+} strains showed a threshold activation at a ~1 nM concentration [1]. Since we do not know the processivity of the Opp transporter and the resulting intracellular concentration in sXIP, we performed a simulation for sXIP uptake gradient, starting with a maximum value and considering that every molecule added to the medium is imported by the cells. To model this, we assumed that at t = 120 min, XIP concentration in the medium reach suddenly 10^{-9} M. Direct uptake through the Opp transporter results in an intracellular concentration given by the equation:

$$[XIP]_{cell} = \frac{[XIP]_{out}}{X(t=120)} \text{ [mol.cell}^{-1}\text{]}$$

Where $[XIP]_{cell}$ is the intracellular concentration of XIP, $[XIP]_{out}$ is the extracellular concentration of sXIP added and $X(t=120)$ is the bacterial concentration in the medium at the time t = 120 min. Because a complete uptake of every single sXIP molecule present in the medium by the cells is rather unrealistic, we chose a range of total amount of sXIP taken up by the cell weaker than the one calculated for a complete uptake with a sXIP concentration in the medium of 10^{-9} M. We then generated Fig. 9, showing the effect of sXIP addition on the different variables of the system.

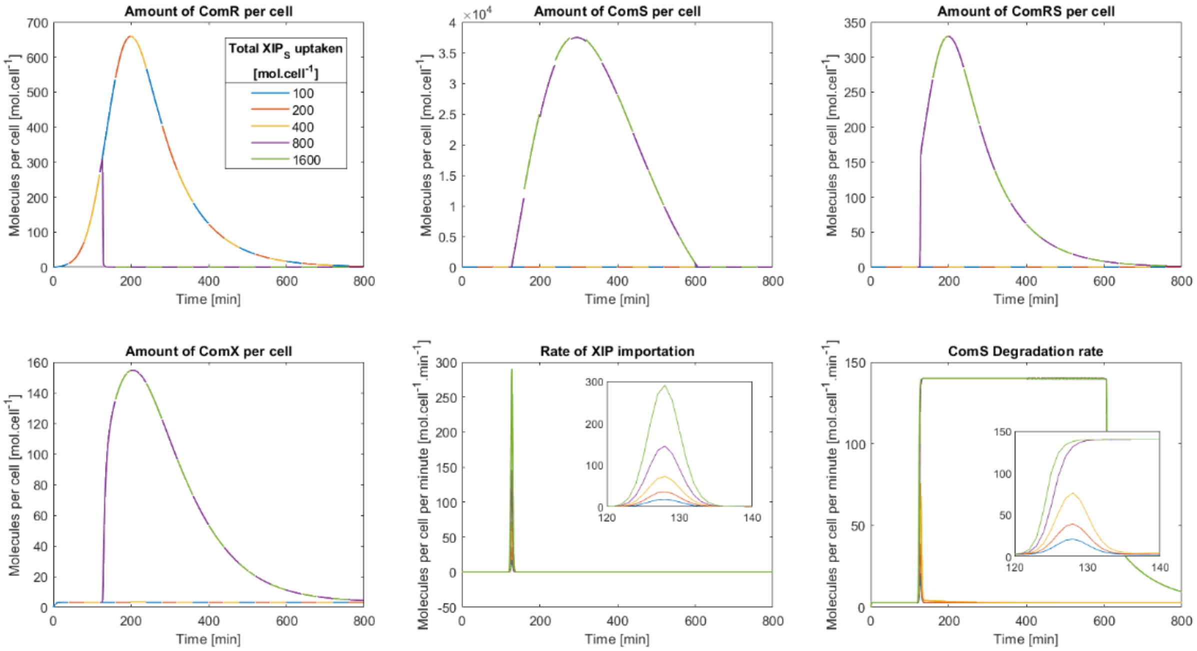

Figure 9. Modeling of sXIP importation efficiency and its effect on competence activation. ComR, ComS, ComRS, ComX abundance, XIP importation rate, and ComS degradation rate were computed over time depending on $_S$XIP uptake. Total amount of $_S$XIP molecules imported through the Opp transporter are depicted by color (blue=100, orange=200 ComR, yellow=400, purple =800 and green=1600 mol.cell^{-1}). Those values correspond to an intracellular XIP concentration of 300, 600, 1200, 2400 and 4800 nM.

We found an intracellular concentration of 1.5 µM (500 molecules per cell) sufficient to overcome the degradation rate through the degradation machinery (*deg*) and trigger competence. Regarding the experimental data showing an effect starting at 1 nM, it would suggest that the Opp system is quite efficient.

To investigate and compare the effect of an intracellular overexpression of *xip*, we modeled a constant production of ComS over time and checked how the overexpression was able to activate the system (Fig. 10).

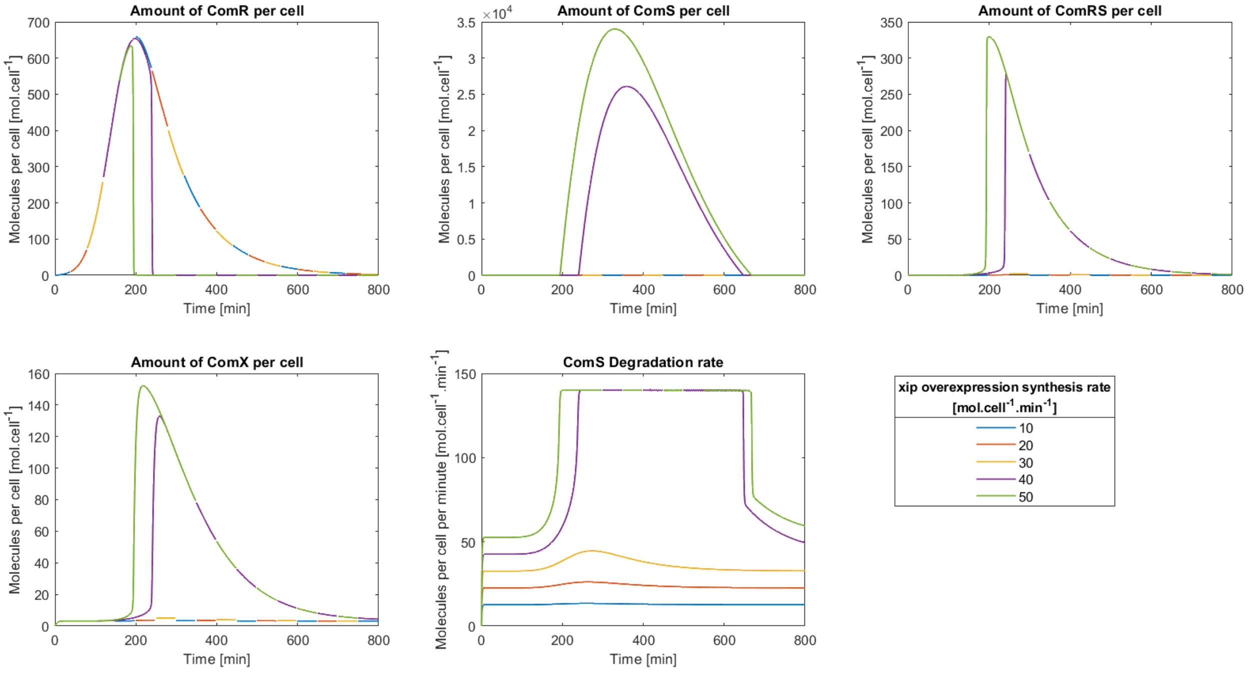

Figure 10: Modeling of xip overexpression and its effect on competence activation. ComR, ComS, ComRS, ComX abundance, and ComS degradation rate, were computed over time depending on the *xip* overexpression level. Computed XIP synthesis rates are depicted by color (blue=15, orange=20, yellow=25, purple =30 and green=35 mol.cell^{-1}). Those values correspond to a cumulative intracellular ComS concentration of 2250, 3656, 5063, 6469 and 7875 mol.cell^{-1}, respectively.

We then computed the total amount of ComS produced until competence was activated (t = 225 min for the highest *xip* overexpression) through *xip* overexpression and compare it to the sXIP quantity necessary for competence activation. We found a much higher ComS value produced through overexpression (6469 mol.cell^{-1}) than through sXIP addition (500 mol.cell^{-1}). This difference is explained by the discrepancy between the two modes of ComS production. In the first one, the overexpression is gradual and rather slow, giving the time for the negative player *deg* to degrade it. By contrast, sXIP addition results in a sharp increase of ComS, directly overcoming the *deg* capacity. Of note, those speculations are based on the suggested high processivity of the Opp transporter [18]. Nevertheless, regarding the experimental reactivity of the system to sXIP addition, this can reasonably be hypothesized.

Table 1: Final parameters values used in the model

Parameter	Description	Value	Unit	Origin
$b_R(t)$	Basal synthesis rate of ComR (fluctuates over time)	0-12	[mol.cell^{-1}.min^{-1}]	Experimental
d_R	ComR degradation rate constant	0.01	[min^{-1}]	[2]
k_{RS}	Complex formation rate constant	10^{-6}	[cell^3.mol^{-3}.min^{-1}]	Free parameter
n	Hill coefficient	2	[ ]	[4]
max_S	Maximum synthesis rate of ComS upon ComRS activation	1000	[mol.cell^{-1}.min^{-1}]	Free parameter
K_S	Required concentration of ComRS for half maximum synthesis rate of ComS	166	[mol.cell^{-1}]	[2]
b_S	Basal synthesis rate of ComS constant	0.0026-0.031	[ ]	Experimental & Free parameter
d_S	ComS degradation rate constant	0.01	[min^{-1}]	Free parameter
d_{RS}	ComRS degradation rate constant	0.01	[min^{-1}]	[2]
max_x	Maximum synthesis rate of ComX upon ComRS activation	60	[mol.cell^{-1}.min^{-1}]	Free parameter
K_X	Required concentration of ComRS for half maximum synthesis rate of ComX	322	[mol.cell^{-1}]	[2]
b_X	Basal synthesis rate of ComX constant	0.0107	[ ]	Experimental
d_X	ComX degradation rate constant	0.2	[min^{-1}]	[2]
$k_{cat} \times deg$	Basal number of XIP-peptidase molecules times specific degradation rate	140	[mol.cell^{-1}.min^{-1}]	Free parameter
K_{deg}	Required concentration of ComS for half maximum degradation rate	1	[mol.cell^{-1}]	Free parameter

REFERENCES

1. Knoops A, Vande Capelle F, Fontaine L, Verhaegen M, Mignolet J, Goffin P, et al. The CovRS Environmental Sensor Directly Controls the ComRS Signaling System To Orchestrate Competence Bimodality in Salivarius Streptococci. Biswas I, editor. MBio. 2022 [cited 14 Jan 2022]. doi:10.1128/mbio.03125-21

2. Haustenne L, Bastin G, Hols P, Fontaine L. Modeling of the ComRS signaling pathway reveals the limiting factors controlling competence in *Streptococcus thermophilus*. Front Microbiol. 2015;6. doi:10.3389/fmicb.2015.01413

3. Mignolet J, Fontaine L, Sass A, Nannan C, Mahillon J, Coenye T, et al. Circuitry Rewiring Directly Couples Competence to Predation in the Gut Dweller *Streptococcus salivarius*. Cell Rep. 2018;22: 1627–1638. doi:10.1016/j.celrep.2018.01.055

4. Talagas A, Fontaine L, Ledesma-García L, Mignolet J, Li de la Sierra-Gallay I, Lazar N, et al. Structural Insights into Streptococcal Competence Regulation by the Cell-to-Cell Communication System ComRS. Annu Rev Genet. 2017;12: e1005980. doi:10.1371/journal.ppat.1005980

5. Ledesma-Garcia L, Thuillier J, Guzman-Espinola A, Ensinck I, de la Sierra-Gallay IL, Lazar N, et al. Molecular dissection of pheromone selectivity in the competence signaling system ComRS of streptococci. Proc Natl Acad Sci U S A. 2020;117: 7745–7754. doi:10.1073/pnas.1916085117

6. Zaccaria E, Wells JM, Baarlen P Van. Metabolic Context of the Competence- Induced Checkpoint for Cell Replication in *Streptococcus suis*. PLoS One. 2016;11: 1–13. doi:10.1371/journal.pone.0153571

7. Son M, Ahn S-J, Guo Q, Burne RA, Hagen SJ. Microfluidic study of competence regulation in *Streptococcus mutans*: environmental inputs modulate bimodal and unimodal expression of comX. Mol Microbiol. 2012;86: 258–72. doi:10.1111/j.1365-2958.2012.08187.x

8. Ricomini Filho AP, Khan R, Åmdal HA, Petersen FC. Conserved Pheromone Production, Response and Degradation by *Streptococcus mutans*. Front Microbiol. 2019;10: 2140. doi:10.3389/fmicb.2019.02140

9. Gardan R, Besset C, Gitton C, Guillot A, Fontaine L, Hols P, et al. Extracellular life cycle of ComS, the competence-stimulating peptide of *Streptococcus thermophilus*. J Bacteriol. 2013;195: 1845–55. doi:10.1128/JB.02196-12

10. Kaspar J, Shields RC, Burne RA. Competence inhibition by the XrpA peptide encoded within the *comX* gene of *Streptococcus mutans*. Mol Microbiol. 2018;109: 345–364. doi:10.1111/mmi.13989

11. Schnorpfeil A, Kranz M, Kovács M, Kirsch C, Gartmann J, Brunner I, et al. Target evaluation of the non-coding csRNAs reveals a link of the two-component regulatory system CiaRH to competence control in *Streptococcus pneumoniae* R6. Mol Microbiol. 2013;89: 334–349. doi:10.1111/mmi.12277

12. Laux A, Sexauer A, Sivaselvarajah D, Kaysen A, Brückner R. Control of competence by related non-coding csRNAs in *Streptococcus pneumoniae* R6. Front Genet. 2015;6: 246.

doi:10.3389/fgene.2015.00246

13. Underhill SAM, Shields RC, Burne RA, Hagen SJ. Carbohydrate and PepO control bimodality in competence development by *Streptococcus mutans*. Mol Microbiol. 2019;112: 1388–1402. doi:10.1111/mmi.14367

14. Wilkening R V, Chang JC, Federle MJ. PepO, a CovRS-controlled endopeptidase, disrupts *Streptococcus pyogenes* quorum sensing. Mol Microbiol. 2016;99: 71–87. doi:10.1111/mmi.13216

15. Li G-W, Burkhardt D, Gross C, Weissman JS. Quantifying absolute protein synthesis rates reveals principles underlying allocation of cellular resources. Cell. 2014;157: 624–35. doi:10.1016/j.cell.2014.02.033

16. Elowitz MB, Levine AJ, Siggia ED, Swain PS. Stochastic Gene Expression in a Single Cell. Science 2002;297: 1183–1186. doi:10.1126/SCIENCE.1070919

17. Taniguchi Y, Choi PJ, Li GW, Chen H, Babu M, Hearn J, et al. Quantifying E. coli proteome and transcriptome with single-molecule sensitivity in single cells. Science 2010;329: 533–538. doi:10.1126/science.1188308

18. Babel H, Naranjo-Meneses P, Trauth S, Schulmeister S, Malengo G, Sourjik V, et al. Ratiometric population sensing by a pump-probe signaling system in *Bacillus subtilis*. Nat Commun. 2020;11. doi:10.1038/s41467-020-14840-w
