# Supplementary material for: Competence shut-off by intracellular pheromone degradation in salivarius streptococci
Source: PLoS Genet. 2022 May 25;18(5):e1010198. doi: 10.1371/journal.pgen.1010198 (PMC9173638; doi:10.1371/journal.pgen.1010198)
Supplement: S2 Appendix — (DOCX) [file pgen.1010198.s016.docx]

function ssa_model()

%%

% To run our model, please copy-paste this text in a matlab file named

% 'ssa_model.m' and save it in the same folder as the

% excel datasheet named dataset1.xlsx

%

% This code contains :

% the model calibration (all parameters definitions),

% the model validation,

% and the model predictions.

%

% It takes approximately 60 minutes to run.

%

% If you have any question, feel free to contact Mr Adrien Knoops :

% adrien.knoops@uclouvain.be

%

clc

clear all

close all

%% defining constants

%%%%%%%%%%%%%%%%%%%%%

tic

dS = 20; % Degradation rate constant of ComS [MIN^-1]

% (Free parameter)

dRS = 0.01; % Degradation rate constant of ComRS [MIN^-1]

% (cfr. Haustenne et al., 2015)

max_s = 100; % Maximum comS production [MOL/CEL.MIN]

% (experimental parameter and based on Filho et al., 2019)

KcomRS_S = 161; % Required concentration of ComRS for half-maximum synthesis

% rate of ComS [mol x cell^-1] (cfr. Haustenne et al., 2015)

bS = 0.0026; % Constitutive comS expression [mol/cell.min](Experimental)

% (X200 upon activation)

krs = 10e-7; % Complex formation rate constant [mol^-4 x cell^4 x min^-1]

% (Free parameter)

n= 2; % Degree of ComRS complex oligomerization

% (cfr. Talagas et al., 2016)

dR = 0.01; % Degradation rate constant of ComR [min^-1]

% (Haustenne et al., 2015)

max_x = 20 ; % Maximum comX production [MOL/CEL.MIN] (Free parameter)

bX = 0.0107; % Basal rate of comX (Experimental) (X100 upon activation)

dx = 0.2; % Degradation rate of ComX (Haustenne et al., 2015)

KcomRS_X = 322; % Required concentration of ComRS for half-maximum synthesis

% rate of ComX [mol x cell^-1] (cfr. Haustenne et al., 2015)

echt = 1; % ComR overexpression factor (modelisation parameter)

%% DETERMINING EXPERIMENTAL PARAMETERS

disp('Determining experimental parameters...')

%% 1. Determining bR(t)

%%%%%%%%%%%%%%%%%%%%%%%%%%%%%%%%%%%%%%%%%%%%%%%%%%%%%%%%%%%%%%%%%%%%%%%%%%%

% 1.1 Let's compute growth curve and mu(t)

% (PcomR-lux)

%%%%%%%%%%%%%%%%%%%%%%%%%%%%%%%%%%%%%%%%%%

OD_600_r = xlsread('dataset1.xlsx','PcomR_lux','J3:J49');

Xm_r = OD_600_r*5*10^7; %5*10^7 = number of cells per OD unit

t_r = xlsread('dataset1.xlsx','PcomR_lux','B3:B49');

% Optimized sigmoïd function :

x2_r = [0.032 255]; %optimized parameters

X_r = @(x) (max(Xm_r)-min(Xm_r))./(1+exp(-x2_r(1)*(x-x2_r(2))))+min(Xm_r);

figure

subplot(2,1,1)

plot(t_r,Xm_r,'rx',t_r,X_r(t_r),'b')

title('PcomR-luxAB : Growth over time')

xlabel('Time [min]','Fontsize',13)

ylabel('Number of cells [cells/ml]')

hold on

%Sigmoïd derivation to find mu(t) :

mu_r_ = @(x) (max(Xm_r)-min(Xm_r)).*(x2_r(1).*(exp(-x2_r(1)*(x-x2_r(2)))))...

.*((1+exp(-x2_r(1)*(x-x2_r(2)))).^(-2)).*(1./X_r(x));

% mu = dX/dt * 1/X(t) = X(t)'/X(t)^2 * 1/X(t)

subplot(2,1,2)

plot(t_r,mu_r_(t_r),'-b');

title('PcomR-luxAB : Growth rate over time')

xlabel('Time [min]')

ylabel('Growth rate [1/min]')

% 1.2 Let's compute comR production rate for activated competent cells

% (PcomR-lux)

%%%%%%%%%%%%%%%%%%%%%%%%%%%%%%%%%%%%%%%%%%%%%%%%%%%%%%%%%%%%%%%%%%%%%%%

%Let's get the measurement in RLU

RLU_r = xlsread('dataset1.xlsx','PcomR_lux','I3:I49');

figure

subplot(3,2,1)

plot(t_r,RLU_r,'r')

title('A','Fontsize',20)

ylabel({'Expression of PcomR';'[RLU]'},'Fontsize',13)

xlabel('Time [min]','Fontsize',13)

axis([0 500 0 inf])

hold on

% Let's transform the RLU in RLU/cell

RLU_r = RLU_r./X_r(t_r);

subplot(3,2,2)

plot(t_r,RLU_r,'r')

title('B','Fontsize',20)

ylabel({'Expression of PcomR';'[RLU/cell]'},'Fontsize',13)

xlabel('Time [min]','Fontsize',13)

axis([0 500 0 inf])

% Approximation of these curves by skewed gaussians

x3_r= [0.24 117.7325 80.3264 1.75];

RLU_r_ = @(x) (x3_r(1)*(1/(x3_r(3)*sqrt(2*pi)))...

*exp((-((x-x3_r(2))/x3_r(3)).^2)./2)...

.*(1+erf(x3_r(4)*((x-x3_r(2))./x3_r(3))./(sqrt(2)))));

subplot(3,2,3)

plot(t_r,RLU_r,'-r',t_r,RLU_r_(t_r),'-b');

title('C','Fontsize',20)

ylabel({'Expression of PcomR';'[RLU/cell]'},'Fontsize',13)

xlabel('Time [min]','Fontsize',13)

axis([0 500 0 inf]);

hold on

% Computation of the production rates

d_lux = log(2)/45; % T 1/2 = 45' for luciferase

% Derivative : dR/dt in [RLU/cell*min]

derR_r_ = @(x) diff(RLU_r_(x))./diff(x);

derR_r = [derR_r_(t_r(1:2)); derR_r_(t_r(:))];

%Because the new vector has n-1 values, we add a value at the beginning

%of the vector to have the same size as t

%transforming derivative into comR production throug light-protein amount

%equation (see Haustenne et al., 2015)

subplot(3,2,4)

prodR = derR_r + d_lux*RLU_r_(t_r) + mu_r_(t_r).*RLU_r_(t_r);

plot(t_r,prodR,'r')

title('D','Fontsize',20)

ylabel({'Lux production rate';' [RLU/cell.min]'},'Fontsize',13)

xlabel('Time [min]','Fontsize',13)

hold on

% Smoothing the negative part (negative production is impossible).

% To do this, we approximate the curves by new skewed gaussians.

x4=[7.4*10e-4 150 47 0];

prodRplus_r = @(x) (x4(1)*(1/(x4(3)*sqrt(2*pi)))...

*exp((-((x-x4(2))/x4(3)).^2)./2).*...

(1+erf(x4(4)*((x-x4(2))./x4(3))./(sqrt(2)))));

subplot(3,2,5)

plot(t_r,prodR,'r',t_r,prodRplus_r(t_r),'b')

title('E','Fontsize',20)

ylabel({'Lux production rate';'[RLU/cell.min]'},'Fontsize',13)

xlabel('Time [min]','Fontsize',13)

hold on

% Scaling the comR production --> find the correct RLUmol factor

% [RLU/molecules] To do this, we use Western Blot data

% --> ~400 molecules/cell at OD of 1.1 which corresponds in our data to

% 280 min of growth) We use the ODE dcomR/dt = 1/RLUmol*prodRplus_r-dr*comR

RLUmol = 10^(-5);

valuess=NaN(100,1);

i=1;

function deriv = dcomR(t,statevar)

cR = statevar(1) ;

dcoR = ((i*0.05*RLUmol)^(-1))*prodRplus_r(t)-dR*cR;

deriv = dcoR ;

end

for i=1:100

hello = ode23(@dcomR,[0,500],0) ;

cR = hello.y;

valuess(i)=deval(hello,280);

end

valuess_2=(valuess-400).^2;

A = find(valuess_2==min(valuess_2));

RLUmol=A*0.05*RLUmol;

%Using this number to evaluate the production in molecules/cell*min

ProdR_ = @(x) (1/RLUmol).*prodRplus_r(x);

subplot(3,2,6)

plot(t_r,ProdR_(t_r))

title('F','Fontsize',20)

ylabel({'ComR synthesis rate';'[mol/cell.min]'},'Fontsize',13)

xlabel('Time [min]','Fontsize',13)

% Here we have converted the RLU in #molecules (through 1/RLUsurMOL)

%% 2. Determining bS and scaling max_s

%%%%%%%%%%%%%%%%%%%%%%%%%%%%%%%%%%%%%%%%%%%%%%%%%%%%%%%%%%%%%%%%%%%%%%%%%%%

% 2.1 Let's compute growth curve and mu(t) for

% activated competent cells(PcomS-lux Pxyl2-comR xyl0.5%)

%%%%%%%%%%%%%%%%%%%%%%%%%%%%%%%%%%%%%%%%%%%%%

OD_600_s_a = xlsread('dataset1.xlsx','PcomS_lux','T3:T33');

Xm_s_a = OD_600_s_a*5*10^7; %5*10^7 = number of cells per OD unit

t_s_a = xlsread('dataset1.xlsx','PcomS_lux','L3:L33');

% Optimized sigmoïd function :

x2_s_a = [0.027 240]; %optimized parameters

X_s_a = @(x) (max(Xm_s_a)-min(Xm_s_a))...

./(1+exp(-x2_s_a(1)*(x-x2_s_a(2))))+min(Xm_s_a);

figure

subplot(2,1,1)

plot(t_s_a,Xm_s_a,'rx',t_s_a,X_s_a(t_s_a),'b')

title('PcomS-luxAB activated : Growth over time')

xlabel('Time [min]','Fontsize',13)

ylabel('Number of cells [cells/ml]')

hold on

%Sigmoïd derivation to find mu(t) :

mu_s_a_ = @(x) (max(Xm_s_a)-min(Xm_s_a))...

.*(x2_s_a(1).*(exp(-x2_s_a(1)*(x-x2_s_a(2)))))...

.*((1+exp(-x2_s_a(1)*(x-x2_s_a(2)))).^(-2)).*(1./X_s_a(x));

% mu = dX/dt * 1/X(t) = X(t)'/X(t)^2 * 1/X(t)

subplot(2,1,2)

plot(t_s_a,mu_s_a_(t_s_a),'-b');

title('PcomS-lux Activated : Growth rate over time')

ylabel('Growth rate [1/min]')

xlabel('Time [min]')

hold on

% 2.2 Let's compute comS production rate for

% activated competent cells(PcomS-lux Pxyl2-comR xylose 0.5%)

%%%%%%%%%%%%%%%%%%%%%%%%%%%%%%%%%%%%%%%%%%%%

%Let's get the measurement in RLU

RLU_s_a = xlsread('dataset1.xlsx','PcomS_lux','S2:S33');

figure

subplot(3,2,1)

plot(t_s_a,RLU_s_a,'r')

title('A','Fontsize',20)

ylabel({'Expression of PcomS';'[RLU]'},'Fontsize',13)

xlabel('Time [min]','Fontsize',13)

axis([0 600 0 inf])

hold on

% Let's transform the RLU in RLU/cell

RLU_s_a = RLU_s_a./X_s_a(t_s_a);

subplot(3,2,2)

plot(t_s_a,RLU_s_a,'r')

title('B','Fontsize',20)

ylabel({'Expression of PcomS';'[RLU/cell]'},'Fontsize',13)

xlabel('Time [min]','Fontsize',13)

axis([0 600 0 inf])

% Approximation of these curves by skewed gaussians

x3_s_a= [13 25 80 6 35 195 40 0];

RLU_s_a_ = @(x) (x3_s_a(1)*(1/(x3_s_a(3)*sqrt(2*pi)))...

*exp((-((x-x3_s_a(2))/x3_s_a(3)).^2)./2)...

.*(1+erf(x3_s_a(4)*((x-x3_s_a(2))./x3_s_a(3))./(sqrt(2)))))...

+ (x3_s_a(5)*(1/(x3_s_a(7)*sqrt(2*pi)))...

*exp((-((x-x3_s_a(6))/x3_s_a(7)).^2)./2)...

.*(1+erf(x3_s_a(8)*((x-x3_s_a(6))./x3_s_a(7))./(sqrt(2)))));

subplot(3,2,3)

plot(t_s_a,RLU_s_a,'-r',t_s_a,RLU_s_a_(t_s_a),'-b');

title('C','Fontsize',20)

ylabel({'Expression of PcomS';'[RLU/cell]'},'Fontsize',13)

xlabel('Time [min]','Fontsize',13)

axis([0 600 0 inf]);

hold on

% Computation of the production rates

d_lux = log(2)/45; % T 1/2 = 45' for luciferase

% Derivative : dR/dt in [RLU/cell*min]

derR_s_a_ = @(x) diff(RLU_s_a_(x))./diff(x);

derR_s_a = [derR_s_a_(t_s_a(1:2)); derR_s_a_(t_s_a(:))];

%Because the new vector has n-1 values, we add a value at the beginning

%of the vector to have the same size as t

%transforming derivative into comS production throug light-protein amount

%equation (see Haustenne et al., 2015)

subplot(3,2,4)

prodS_a = derR_s_a + d_lux*RLU_s_a_(t_s_a) + mu_s_a_(t_s_a).*RLU_s_a_(t_s_a);

plot(t_s_a,prodS_a,'r')

title('D','Fontsize',20)

ylabel({'Lux production rate';' [RLU/cell.min]'},'Fontsize',13)

xlabel('Time [min]','Fontsize',13)

hold on

% Smoothing the negative part (negative production is impossible).

% To do this, we approximate the curves by new skewed gaussians.

x4=[2.3*10e-2 50 30 -2 1.07*10e-1 203 45 -2];

prodSplus_a = @(x) (x4(1)*(1/(x4(3)*sqrt(2*pi)))...

*exp((-((x-x4(2))/x4(3)).^2)./2).*...

(1+erf(x4(4)*((x-x4(2))./x4(3))./(sqrt(2)))))...

+ (x4(5)*(1/(x4(7)*sqrt(2*pi)))*...

exp((-((x-x4(6))/x4(7)).^2)./2)...

.*(1+erf(x4(8)*((x-x4(6))./x4(7))./(sqrt(2)))));

subplot(3,2,5)

plot(t_s_a,prodS_a,'r',t_s_a,prodSplus_a(t_s_a),'b')

title('E','Fontsize',20)

ylabel({'Lux production rate';'[RLU/cell.min]'},'Fontsize',13)

xlabel('Time [min]','Fontsize',13)

hold on

% Scaling the comS production --> Use of the RLUmol factor used for PcomR

RLUmol=5.5*10^(-6);

%Using this number to evaluate the production in molecules/cell*min

ProdS_a = @(x) (1/RLUmol).*((x4(1)*(1/(x4(3)*sqrt(2*pi)))...

*exp((-((x-x4(2))/x4(3)).^2)./2).*...

(1+erf(x4(4)*((x-x4(2))./x4(3))./(sqrt(2)))))...

+ (x4(5)*(1/(x4(7)*sqrt(2*pi)))*...

exp((-((x-x4(6))/x4(7)).^2)./2)...

.*(1+erf(x4(8)*((x-x4(6))./x4(7))./(sqrt(2))))));

subplot(3,2,6)

plot(t_s_a,ProdS_a(t_s_a))

title('F','Fontsize',20)

ylabel({'ComS synthesis rate';'[mol/cell.min]'},'Fontsize',13)

xlabel('Time [min]','Fontsize',13)

% Here we have converted the RLU in #molecules (through 1/RLUmol)

% 2.3 Let's now compute growth curve and mu(t) for

% unactivated competent cells(PcomS-lux Pxyl2-comR xylose 0%)

%%%%%%%%%%%%%%%%%%%%%%%%%%%%%%%%%%%%%%%%%%%%%%%%%%

OD_600_s_u = xlsread('dataset1.xlsx','PcomS_lux','J3:J33');

Xm_s_u=OD_600_s_u*5*10^7; %5*10^8 = number of cells per OD unit

t_s_u = xlsread('dataset1.xlsx','PcomS_lux','B3:B33');

% Optimized sigmoïd function :

x2_s_u = [0.035 230]; %optimized parameters

X_s_u = @(x) (max(Xm_s_u)-min(Xm_s_u))...

./(1+exp(-x2_s_u(1)*(x-x2_s_u(2))))+min(Xm_s_u);

figure

subplot(2,1,1)

plot(t_s_u,Xm_s_u,'rx',t_s_u,X_s_u(t_s_u),'b')

title('PcomS-lux Unactivated : Growth [cells/ml]')

hold on

%Sigmoïd derivation to find mu(t) :

mu_s_u_ = @(x) (max(Xm_s_u)-min(Xm_s_u))...

.*(x2_s_u(1).*(exp(-x2_s_u(1)*(x-x2_s_u(2)))))...

.*((1+exp(-x2_s_u(1)*(x-x2_s_u(2)))).^(-2)).*(1./X_s_u(x));

% mu = dX/dt * 1/X(t) = X(t)'/X(t)^2 * 1/X(t)

subplot(2,1,2)

plot(t_s_u,mu_s_u_(t_s_u),'-b');

title('PcomS-lux Unactivated : Growth rate [1/min]')

%2.4 Let's finally compute comS production rate for

% unactivated competent cells(PcomS-lux Pxyl2-comR xylose 0%)

%%%%%%%%%%%%%%%%%%%%%%%%%%%%%%%%%%%%%%%%%%%%%%%%%%%

%Let's get the measurement in RLU

RLU_s_u = xlsread('dataset1.xlsx','PcomS_lux','I3:I33');

figure

subplot(3,2,1)

plot(t_s_u,RLU_s_u,'r')

title('A','Fontsize',20)

ylabel({'Expression of PcomS';'[RLU]'},'Fontsize',13)

xlabel('Time [min]','Fontsize',13)

axis([0 600 0 inf])

hold on

% Let's transform the RLU in RLU/cell

RLU_s_u = RLU_s_u./X_s_u(t_s_u);

subplot(3,2,2)

plot(t_s_u,RLU_s_u,'r')

title('B','Fontsize',20)

ylabel({'Expression of PcomS';'[RLU/cell]'},'Fontsize',13)

xlabel('Time [min]','Fontsize',13)

axis([0 600 0 inf])

% Approximation of these curves by skewed gaussians

x3_s_u= [4.3*10^(-2) 70 70 2 7*10^(-2) 190 60 2.5];

RLU_s_u_ = @(x) (x3_s_u(1)*(1/(x3_s_u(3)*sqrt(2*pi)))...

*exp((-((x-x3_s_u(2))/x3_s_u(3)).^2)./2)...

.*(1+erf(x3_s_u(4)*((x-x3_s_u(2))./x3_s_u(3))./(sqrt(2)))))...

+ (x3_s_u(5)*(1/(x3_s_u(7)*sqrt(2*pi)))...

*exp((-((x-x3_s_u(6))/x3_s_u(7)).^2)./2)...

.*(1+erf(x3_s_u(8)*((x-x3_s_u(6))./x3_s_u(7))./(sqrt(2)))));

subplot(3,2,3)

plot(t_s_u,RLU_s_u,'-r',t_s_u,RLU_s_u_(t_s_u),'-b');

title('C','Fontsize',20)

ylabel({'Expression of PcomS';'[RLU/cell]'},'Fontsize',13)

xlabel('Time [min]','Fontsize',13)

axis([0 600 0 inf]);

hold on

% Computation of the production rates

d_lux = log(2)/45; % T 1/2 = 45' for luciferase

% Derivative : dR/dt in [RLU/cell*min]

derR_s_u_ = @(x) diff(RLU_s_u_(x))./diff(x);

derR_s_u = [derR_s_u_(t_s_u(1:2)); derR_s_u_(t_s_u(:))];

%Because the new vector has n-1 values, we add a value at the beginning

%of the vector to have the same size as t

%transforming derivative into comS production throug light-protein amount

%equation (see Haustenne et al., 2015)

prodS_u = derR_s_u + d_lux*RLU_s_u_(t_s_u) + mu_s_u_(t_s_u).*RLU_s_u_(t_s_u);

subplot(3,2,4)

plot(t_s_u,prodS_u,'r')

title('D','Fontsize',20)

ylabel({'Lux production rate';' [RLU/cell.min]'},'Fontsize',13)

xlabel('Time [min]','Fontsize',13)

hold on

% Smoothing the negative part (negative production is impossible).

% To do this, we approximate the curves by new skewed gaussians.

x4=[1*10e-4 60 55 1 2*10e-4 205 22 0];

prodSplus_u = @(x) (x4(1)*(1/(x4(3)*sqrt(2*pi)))...

*exp((-((x-x4(2))/x4(3)).^2)./2).*...

(1+erf(x4(4)*((x-x4(2))./x4(3))./(sqrt(2)))))...

+ (x4(5)*(1/(x4(7)*sqrt(2*pi)))*...

exp((-((x-x4(6))/x4(7)).^2)./2)...

.*(1+erf(x4(8)*((x-x4(6))./x4(7))./(sqrt(2)))));

subplot(3,2,5)

plot(t_s_u,prodS_u,'r',t_s_u,prodSplus_u(t_s_u),'b')

title('E','Fontsize',20)

ylabel({'Lux production rate';'[RLU/cell.min]'},'Fontsize',13)

xlabel('Time [min]','Fontsize',13)

hold on

% Scaling the comS production --> Use of the RLUmol factor used for PcomR

RLUmol=5.5*10^(-6);

%Using this number to evaluate the production in molecules/cell*min

ProdS_u = @(x) (1/RLUmol).*((x4(1)*(1/(x4(3)*sqrt(2*pi)))...

*exp((-((x-x4(2))/x4(3)).^2)./2).*...

(1+erf(x4(4)*((x-x4(2))./x4(3))./(sqrt(2)))))...

+ (x4(5)*(1/(x4(7)*sqrt(2*pi)))*...

exp((-((x-x4(6))/x4(7)).^2)./2)...

.*(1+erf(x4(8)*((x-x4(6))./x4(7))./(sqrt(2))))));

subplot(3,2,6)

plot(t_s_u,ProdS_u(t_s_u))

title('F','Fontsize',20)

ylabel({'ComS synthesis rate';'[mol/cell.min]'},'Fontsize',13)

xlabel('Time [min]','Fontsize',13)

% Here we have converted the RLU in #molecules (through 1/RLUmol)

%Comparing max synthesis rate of ComS in activated cells and in unactivated

%cells :

A_over_U=max(ProdS_a(t_s_a))/max(ProdS_u(t_s_u));

bS=1/(A_over_U-1);

%% 3. Determining scaling bx

%%%%%%%%%%%%%%%%%%%%%%%%%%%%%%%%%%%%%%%%%%%%%%%%%%%%%%%%%%%%%%%%%%%%%%%%%%%

% 3.1 Let's compute growth curve and mu(t) for

% activated competent cells(PcomX-lux Pxyl2-comR xyl0.5%)

%%%%%%%%%%%%%%%%%%%%%%%%%%%%%%%%%%%%%%%%%%%%%%%

OD_600_x_a = xlsread('dataset1.xlsx','PcomX_lux','T3:T35');

% Growth curve (OD600)

Xm_x_a = OD_600_x_a*5*10^7; %5*10^8 = number of cells per OD unit

t_x_a = xlsread('dataset1.xlsx','PcomX_lux','L3:L35');

% Optimized sigmoïd function :

x2_x_a = [0.0235 270]; %optimized parameters

X_x_a = @(x) (max(Xm_x_a)-min(Xm_x_a))./(1+exp(-x2_x_a(1)*(x-x2_x_a(2))))...

+min(Xm_x_a);

figure

subplot(2,1,1)

plot(t_x_a,Xm_x_a,'rx',t_x_a,X_x_a(t_x_a),'b')

title('PcomX-luxAB activated : Growth over time')

xlabel('Time [min]','Fontsize',13)

ylabel('Number of cells [cells/ml]')

hold on

%Sigmoïd derivation to find mu(t) :

mu_s_a_ = @(x) (max(Xm_x_a)-min(Xm_x_a))...

.*(x2_x_a(1).*(exp(-x2_x_a(1)*(x-x2_x_a(2)))))...

.*((1+exp(-x2_x_a(1)*(x-x2_x_a(2)))).^(-2)).*(1./X_x_a(x));

% mu = dX/dt * 1/X(t) = X(t)'/X(t)^2 * 1/X(t)

subplot(2,1,2)

plot(t_x_a,mu_s_a_(t_x_a),'-b');

title('PcomX-lux Activated : Growth rate over time')

ylabel('Growth rate [1/min]')

xlabel('Time [min]')

hold on

% 3.2 Let's compute comS production rate for

% activated competent cells(PcomX-lux Pxyl2-comR xylose 0.5%)

%%%%%%%%%%%%%%%%%%%%%%%%%%%%%%%%%%%%%%%%%%%%

%Let's get the measurement in RLU

RLU_x_a = xlsread('dataset1.xlsx','PcomX_lux','S3:S35');

figure

subplot(3,2,1)

plot(t_x_a,RLU_x_a,'r')

title('A','Fontsize',20)

ylabel({'Expression of PcomX';'[RLU]'},'Fontsize',13)

xlabel('Time [min]','Fontsize',13)

axis([0 600 0 inf])

hold on

% Let's transform the RLU in RLU/cell

RLU_x_a = RLU_x_a./X_x_a(t_x_a);

subplot(3,2,2)

plot(t_x_a,RLU_x_a,'r')

title('B','Fontsize',20)

ylabel({'Expression of PcomX';'[RLU/cell]'},'Fontsize',13)

xlabel('Time [min]','Fontsize',13)

axis([0 600 0 inf])

% Approximation of these curves by skewed gaussians

x3_x_a= [0.21 160 10 0 0.75 170 65 3];

RLU_x_a_ = @(x) (x3_x_a(1)*(1/(x3_x_a(3)*sqrt(2*pi)))...

*exp((-((x-x3_x_a(2))/x3_x_a(3)).^2)./2)...

.*(1+erf(x3_x_a(4)*((x-x3_x_a(2))./x3_x_a(3))./(sqrt(2)))))...

+ (x3_x_a(5)*(1/(x3_x_a(7)*sqrt(2*pi)))...

*exp((-((x-x3_x_a(6))/x3_x_a(7)).^2)./2)...

.*(1+erf(x3_x_a(8)*((x-x3_x_a(6))./x3_x_a(7))./(sqrt(2)))));

subplot(3,2,3)

plot(t_x_a,RLU_x_a,'-r',t_x_a,RLU_x_a_(t_x_a),'-b');

title('C','Fontsize',20)

ylabel({'Expression of PcomX';'[RLU/cell]'},'Fontsize',13)

xlabel('Time [min]','Fontsize',13)

axis([0 600 0 inf]);

hold on

% Computation of the production rates

d_lux = log(2)/45; % T 1/2 = 45' for luciferase

% Derivative : dR/dt in [RLU/cell*min]

derR_x_a_ = @(x) diff(RLU_x_a_(x))./diff(x);

derR_x_a = [derR_x_a_(t_x_a(1:2)); derR_x_a_(t_x_a(:))];

%Because the new vector has n-1 values, we add a value at the beginning

%of the vector to have the same size as t

%transforming derivative into comS production throug light-protein amount

%equation (see Haustenne et al., 2015)

prodX_a = derR_x_a + d_lux*RLU_x_a_(t_x_a) + mu_s_a_(t_x_a).*RLU_x_a_(t_x_a);

subplot(3,2,4)

plot(t_x_a,prodX_a,'r')

title('D','Fontsize',20)

ylabel({'Lux production rate';' [RLU/cell.min]'},'Fontsize',13)

xlabel('Time [min]','Fontsize',13)

hold on

% Smoothing the negative part (negative production is impossible).

% To do this, we approximate the curves by new skewed gaussians.

x7=[1.5*10e-3 155 5 0 0.1*10e-2 200 15 0];

prodXplus_a = @(x) (x7(1)*(1/(x7(3)*sqrt(2*pi)))...

*exp((-((x-x7(2))/x7(3)).^2)./2).*...

(1+erf(x7(4)*((x-x7(2))./x7(3))./(sqrt(2)))))...

+ (x7(5)*(1/(x7(7)*sqrt(2*pi)))*...

exp((-((x-x7(6))/x7(7)).^2)./2)...

.*(1+erf(x7(8)*((x-x7(6))./x7(7))./(sqrt(2)))));

subplot(3,2,5)

plot(t_x_a,prodX_a,'r',t_x_a,prodXplus_a(t_x_a),'b')

title('E','Fontsize',20)

ylabel({'Lux production rate';'[RLU/cell.min]'},'Fontsize',13)

xlabel('Time [min]','Fontsize',13)

hold on

% Scaling the comX production --> Use of a RLUmol factor computed for PcomR-lux

RLUmol=5.5*10^(-6);

%Using this number to evaluate the production in molecules/cell*min

ProdX_a = @(x) (1/RLUmol).*((x7(1)*(1/(x7(3)*sqrt(2*pi)))...

*exp((-((x-x7(2))/x7(3)).^2)./2).*...

(1+erf(x7(4)*((x-x7(2))./x7(3))./(sqrt(2)))))...

+ (x7(5)*(1/(x7(7)*sqrt(2*pi)))*...

exp((-((x-x7(6))/x7(7)).^2)./2)...

.*(1+erf(x7(8)*((x-x7(6))./x7(7))./(sqrt(2))))));

subplot(3,2,6)

plot(t_x_a,ProdX_a(t_x_a))

title('F','Fontsize',20)

ylabel({'ComX synthesis rate';'[mol/cell.min]'},'Fontsize',13)

xlabel('Time [min]','Fontsize',13)

% Here we have converted the RLU in #molecules (through 1/RLUmol)

% 3.3 Let's now compute growth curve and mu(t) for

% unactivated competent cells(PcomX-lux Pxyl2-comR xylose 0%)

%%%%%%%%%%%%%%%%%%%%%%%%%%%%%%%%%%%%%%%%%%%%%%%%%%

OD_600_x_u = xlsread('dataset1.xlsx','PcomX_lux','J3:J33');

Xm_x_u=OD_600_x_u*5*10^7; %5*10^8 = number of cells per OD unit

t_x_u = xlsread('dataset1.xlsx','PcomX_lux','B3:B33');

% Optimized sigmoïd function :

x2_x_u = [0.028 255]; %optimized parameters

X_x_u = @(x) (max(Xm_x_u)-min(Xm_x_u))...

./(1+exp(-x2_x_u(1)*(x-x2_x_u(2))))+min(Xm_x_u);

figure

subplot(2,1,1)

plot(t_x_u,Xm_x_u,'rx',t_x_u,X_x_u(t_x_u),'b')

title('PcomX-lux Unactivated : Growth [cells/ml]')

hold on

%Sigmoïd derivation to find mu(t) :

mu_x_u_ = @(x) (max(Xm_x_u)-min(Xm_x_u)).*(x2_x_u(1)...

.*(exp(-x2_x_u(1)*(x-x2_x_u(2)))))...

.*((1+exp(-x2_x_u(1)*(x-x2_x_u(2)))).^(-2)).*(1./X_x_u(x));

% mu = dX/dt * 1/X(t) = X(t)'/X(t)^2 * 1/X(t)

subplot(2,1,2)

plot(t_x_u,mu_x_u_(t_x_u),'-b');

title('PcomX-lux Unactivated : Growth rate [1/min]')

%2.4 Let's finally compute comX production rate for

% unactivated competent cells(PcomX-lux Pxyl2-comR xylose 0%)

%%%%%%%%%%%%%%%%%%%%%%%%%%%%%%%%%%%%%%%%%%%%%%%%%%%%

%Let's get the measurement in RLU

RLU_x_u = xlsread('dataset1.xlsx','PcomX_lux','I3:I33');

figure

subplot(3,2,1)

plot(t_x_u,RLU_x_u,'r')

title('A','Fontsize',20)

ylabel({'Expression of PcomX';'[RLU]'},'Fontsize',13)

xlabel('Time [min]','Fontsize',13)

axis([0 600 0 inf])

hold on

% Let's transform the RLU in RLU/cell

RLU_x_u = RLU_x_u./X_x_u(t_x_u);

subplot(3,2,2)

plot(t_x_u,RLU_x_u,'r')

title('B','Fontsize',20)

ylabel({'Expression of PcomX';'[RLU/cell]'},'Fontsize',13)

xlabel('Time [min]','Fontsize',13)

axis([0 600 0 inf])

% Approximation of these curves by constant expression

x3_x_u= [1.4*10^(-2) 10 90 200];

RLU_x_u_ = @(x) (x3_x_u(1)*(1/(x3_x_u(3)*sqrt(2*pi)))...

*exp((-((x-x3_x_u(2))/x3_x_u(3)).^2)./2)...

.*(1+erf(x3_x_u(4)*((x-x3_x_u(2))./x3_x_u(3))./(sqrt(2)))));

subplot(3,2,3)

plot(t_x_u,RLU_x_u,'-r',t_x_u,RLU_x_u_(t_x_u),'-b');

title('C','Fontsize',20)

ylabel({'Expression of PcomX';'[RLU/cell]'},'Fontsize',13)

xlabel('Time [min]','Fontsize',13)

axis([0 600 0 inf]);

hold on

% Computation of the production rates

d_lux = log(2)/45; % T 1/2 = 45' for luciferase

% Derivative : dX/dt in [RLU/cell*min]

derR_x_u_ = @(x) diff(RLU_x_u_(x))./diff(x);

derR_x_u = [derR_x_u_(t_x_u(1:2)); derR_x_u_(t_x_u(:))];

%Because the new vector has n-1 values, we add a value at the beginning

%of the vector to have the same size as t

%transforming derivative into comX production throug light-protein amount

%equation (see Haustenne et al., 2015)

prodx_u = derR_x_u + d_lux*RLU_x_u_(t_x_u) + mu_x_u_(t_x_u).*RLU_x_u_(t_x_u);

subplot(3,2,4)

plot(t_x_u,prodx_u,'r')

title('D','Fontsize',20)

ylabel({'Lux production rate';' [RLU/cell.min]'},'Fontsize',13)

xlabel('Time [min]','Fontsize',13)

hold on

% Smoothing the negative part (negative production is impossible).

% To do this, we approximate the curves by new skewed gaussians.

x8=[3.5*10e-5 1 25 1000];

prodXplus_u = @(x) (x8(1)*(1/(x8(3)*sqrt(2*pi)))...

*exp((-((x-x8(2))/x8(3)).^2)./2).*...

(1+erf(x8(4)*((x-x8(2))./x8(3))./(sqrt(2)))));

subplot(3,2,5)

plot(t_x_u,prodx_u,'r',t_x_u,prodXplus_u(t_x_u),'b')

title('E','Fontsize',20)

ylabel({'Lux production rate';'[RLU/cell.min]'},'Fontsize',13)

xlabel('Time [min]','Fontsize',13)

hold on

% Scaling the comX production --> Use of the RLUmol factor used for PcomR

RLUmol=5.5*10^(-6);

%Using this number to evaluate the production in molecules/cell*min

ProdX_u = @(x) (1/RLUmol).*((x8(1)*(1/(x8(3)*sqrt(2*pi)))...

*exp((-((x-x8(2))/x8(3)).^2)./2).*...

(1+erf(x8(4)*((x-x8(2))./x8(3))./(sqrt(2))))));

subplot(3,2,6)

plot(t_x_u,ProdX_u(t_x_u))

title('F','Fontsize',20)

ylabel({'ComX synthesis rate';'[mol/cell.min]'},'Fontsize',13)

xlabel('Time [min]','Fontsize',13)

% Here we have converted the RLU in #molecules (through 1/RLUmol)

%Comparing max synthesis rate of ComS in activated cells and in unactivated

%cells :

A_over_U_x=max(ProdX_a(t_x_a))/max(ProdX_u(t_x_u));

bX=1/(A_over_U_x-1);

disp(' ...done')

%% DETERMINING FREE PARAMETERS

disp('Determining free parameters...')

%% 1.Determing krs, max_s and dS

%%%%%%%%%%%%%%%%%%%%%%%%%%%%%%%%%%%%%%%%%%%%%%%%%%%%%%%%%%%%%%%%%%%%%%%%%%%

tlast = 800 ; % min

time = 0:800;

% 1.1 Defining the ODEs system

%%%%%%%%%%%%%%%%%%%%%%%%%%%%%%%%%%

function deriv = dcomRS(t,statevar)

comR = statevar(1) ;

comS = statevar(2) ;

comRS = statevar(3) ;

comX = statevar(4);

comR_tot = statevar(5);

dcomR = echt*ProdR_(t)-dR*comR-krs*((comR*comS)^n);

dcomS = max_s*((comRS/(comRS+KcomRS_S))+bS)-dS*comS-krs*((comR*comS)^n);

dcomRS = (1/2)*krs*((comR*comS)^n)-dRS*comRS;

dcomX = max_x*((comRS/(comRS+KcomRS_X))+bX)-dx*comX;

dcomR_tot = echt*ProdR_(t);

deriv = [dcomR;dcomS;dcomRS;dcomX;dcomR_tot] ;

end

%1.2 Run it for two values of ComR and various

% values of krs, dS and max_S

%%%%%%%%%%%%%%%%%%%%%%%%%%%%%%%%%%%%%%%%%%%%%%%%

% We use the values from litterature to start our parametrization:

a=[1 8.5]; %Factor multiplying ComR expression with Pxyl2 induction

b=1:10; %Factor multypling dS

c=1:10; %Factor multiplying max_S

d=1:8;

A=NaN(5,800); %Preparing the matrix for getting results ON and OFF

B=NaN(1,length(b)); %Preparing the matrix for different dS values

C=NaN(length(c),length(b)); %Preparing the matrix for different max_s values

figure

hold on

for l=d % Running ODEs for different krs values

dd=[10e-10 10e-9 10e-8 10e-7 10e-6 10e-5 10e-4 10e-3 10e-2];

r=-9:1:-1;

krs=dd(l);

for k=c % Running ODEs for different max_s

cc=linspace(1,100,10);

max_s=cc(k);

for j=b % Running ODEs for different dS

bb=linspace(1,15,10);

dS=bb(j);

for i=1:2 %running the ODE for ON and OFF State

echt=a(i);

sol = ode23(@dcomRS,[0,tlast],[0 0 0 0 0]) ;

time=1:800;

eval=deval(sol,time);

A(:,:,i)=eval;

end

B(1,j)=max(A(4,:,2))-max(A(4,:,1));

end

C(k,:)=B;

end

subplot(3,3,l)

surf(cc,bb,C)

zlim([0 120])

xlabel('max_s')

ylabel('dS')

zlabel('ComX(ON/OFF)')

tit=sprintf('krs = 10^{%d}',r(l));

title(tit)

hold on

end

% 1.3 Showing Bimodality with parameters obtained

% Generating Multiple plot for different ComR

%%%%%%%%%%%%%%%%%%%%%%%%%%%%%%%%%%%%%%%%%%%%%%%%%%%

% A. Updating parameters

a = [0 1 3.5 6 8.5];

max_s=100;

dS=15;

krs=2*10e-7;

max_x=60;

tlast=800;

% B. Running simulation

figure

hold on

for i=1:5

echt=a(i);

[time, statevars] = ode23(@dcomRS, [0 tlast], [0,0,0,0,0]);

comR = statevars(:,1);

comS = statevars(:,2);

comRS = statevars(:,3);

comX = statevars(:,4);

comR_tot = statevars(:,5);

subplot(2,3,1)

plot(time,comR_tot,'LineWidth',1)

title('Cumulative amount of ComR produced per cell')

hold on

subplot(2,3,2)

plot(time, comR,'LineWidth',1)

title('Amount of ComR per cell')

ylabel('Amount of molecule per cell [MOL/CELL]')

hold on

subplot(2,3,3)

plot(time,comS,'LineWidth',1)

title('Amount of ComS per cell')

hold on

subplot(2,3,4)

plot(time,comRS,'LineWidth',1)

title('Amount of ComRS per cell')

hold on

subplot(2,3,5)

plot(time,comX,'LineWidth',1)

title('Amount of ComX per cell')

hold on

h=findobj(gcf,'type','axes');

set([h.XLabel],'string','Time [min]')

set([h.YLabel],'string','Molecules per cell [mol/cell]')

end

lgd=legend('0 ComR','WT ComR','3.5 x WT ComR', '6 x WT ComR',...

'8.5 x WT ComR','Location','southeast');

lgd.FontSize = 14;

%% 2. Implementing the new DEG term

%%%%%%%%%%%%%%%%%%%%%%%%%%%%%%%%%%%%%%%%%%%%%%%%%%%%%%%%%%%%%%%%%%%%%%%%%%%

%2.1. Parametrization of Kdeg and kcatxdeg

%%%%%%%%%%%%%%%%%%%%%%%%%%%%%%%%%%%%%%%%%%

%A. Parameters update

max_x=60;

dS=0.01;

krs=10e-7;

max_s=1000;

kcat=100;

deg=1;

Kdeg=1;

tlast = 800;

%B. Defining the new ODEs

function deriv_pep = dcomRS_pep(t,statevar)

comR = statevar(1) ;

comS = statevar(2) ;

comRS = statevar(3) ;

comX = statevar(4);

comR_tot = statevar(5);

degradation = statevar(6);

dcomR = echt*ProdR_(t)-dR*comR-krs*((comR*comS)^n);

dcomS = max_s*((comRS/(comRS+KcomRS_S))+bS)-dS*comS-krs*((comR*comS)^n)...

-kcat*deg*((comS)/(comS+Kdeg));

dcomRS = (1/2)*krs*((comR*comS)^n)-dRS*comRS;

dcomX = max_x*((comRS/(comRS+KcomRS_X))+bX)-dx*comX;

dcomR_tot = echt*ProdR_(t);

ddegradation =kcat*deg*((comS)/(comS+Kdeg))-degradation;

deriv_pep = [dcomR;dcomS;dcomRS;dcomX;dcomR_tot;ddegradation] ;

end

%C. Run it for two values of ComR and various values of kcatxpep and Kpep

a=[6 8.5]; %Factor multiplying ComR expression with Pxyl2 induction

b=1:5; %Factor multypling kcatxdeg

c=1:10; %Factor multiplying Kdeg

A=NaN(6,800); %Preparing the matrix for getting results ON and OFF

B=NaN(1,length(b)); %Preparing the matrix for different dS values

C=NaN(length(c),length(b)); %Preparing the matrix for different max_s values

for k=c %Running the ODEs for various values of Kdeg

cc=linspace(0.1,1,10);

Kdeg=cc(k);

for j=b %Running the ODEs for various values of kcat*deg

bb=linspace(1,500,5);

kcat=bb(j);

for i=1:2 %Running the ODE for ON and OFF State

echt=a(i); %the ComR multiplying factor

sol2 = ode23(@dcomRS_pep,[0,tlast],[0,0,0,0,0,0]) ;

time=1:800;

eval2=deval(sol2,time);

A(:,:,i)=eval2;

end

B(1,j)=max(A(2,:,2))-max(A(2,:,1));

end

C(k,:)=B;

end

figure

surf(bb,cc,C)

xlabel('kcat x deg')

ylabel('Kdeg')

zlabel('ComS_{ON/OFF}')

%2.2 Showing bimodality with parameters obtained

%%%%%%%%%%%%%%%%%%%%%%%%%%%%%%%%%%%%%%%%%%%%%%%%

%Generating Multiple plot for different ComR

%A. Updating parameters

kcat=140;

deg=1;

Kdeg=1;

%B. Running simulation

figure

for i=1:5

a=[0 1 3.5 6 8.5];

echt=a(i);

[time,statevarsy] = ode23(@dcomRS_pep,[0,tlast],[0 0 0 0 0 0]) ;

comR = statevarsy(:,1);

comS = statevarsy(:,2);

comRS = statevarsy(:,3);

comX = statevarsy(:,4);

comR_tot = statevarsy(:,5);

degradation = statevarsy(:,6);

subplot(2,3,1)

plot(time,comR_tot,'LineWidth',1)

title('Cumulative amount of ComR produced per cell')

hold on

subplot(2,3,2)

plot(time, comR,'LineWidth',1)

title('Amount of ComR per cell')

ylabel('Amount of molecule per cell [MOL/CELL]')

hold on

subplot(2,3,3)

plot(time,comS,'LineWidth',1)

title('Amount of ComS per cell')

hold on

subplot(2,3,4)

plot(time,comRS,'LineWidth',1)

title('Amount of ComRS per cell')

hold on

subplot(2,3,5)

plot(time,comX,'LineWidth',1)

title('Amount of ComX per cell')

hold on

h=findobj(gcf,'type','axes');

set([h.XLabel],'string','Time [min]')

set([h.YLabel],'string','Molecules per cell [mol/cell]')

subplot(2,3,6)

plot(time,degradation,'LineWidth',1)

title('Degradation rate {\it (deg)}')

ylabel('Molecules per cell per minutes [mol/cell.min]')

hold on

end

lgd=legend('0 ComR','WT ComR','3.5 x WT ComR', '6 x WT ComR',...

'8.5 x WT ComR','Location','southeast');

lgd.FontSize = 14;

disp(' ...done')

%% VALIDATION OF THE MODEL

disp('Validating the model ...')

%% 1. Validation with comS deletion

%%%%%%%%%%%%%%%%%%%%%%%%%%%%%%%%%%%%%%%%%%%%%%%%%%%%%%%%%%%%%%%%%%%%%%%%%%%

%1.1 Defining the new ODEs

%%%%%%%%%%%%%%%%%%%%%%%%%%%

tlast = 800 ; % min

kcat = 140;

deg = 1;

Kdeg = 1;

function deriv_comSdeletion = dcomRS_comSdeletion(t,statevar)

comR = statevar(1) ;

comS = statevar(2) ;

comRS = statevar(3) ;

comX = statevar(4);

prodRtot = statevar(5);

degradation = statevar(6);

dcomR = echt*ProdR_(t)-dR*comR-krs*((comR*comS)^n);

dcomS = 0;

dcomRS = (1/2)*krs*((comR*comS)^n)-dRS*comRS;

dcomX = max_x*((comRS/(comRS+KcomRS_X))+bX)-dx*comX;

dcomRtot = echt*ProdR_(t);

ddegradation =kcat*deg*((comS)/(comS+Kdeg))-degradation;

deriv_comSdeletion = [dcomR;dcomS;dcomRS;dcomX;dcomRtot;ddegradation] ;

end

time=0:800;

figure

for i=1:5

a=[0 1 3.5 6 8.5];

echt=a(i);

sol3 = ode23(@dcomRS_comSdeletion,[0,tlast],[0 0 0 0 0 0]) ;

eval3=deval(sol3,time);

comR=eval3(1,:);

comS=eval3(2,:);

comRS=eval3(3,:);

comX=eval3(4,:);

prodRtot=eval3(5,:);

degradation=eval3(6,:);

subplot(2,3,1)

plot(time,prodRtot,'LineWidth',1)

title('Accumulative amount of ComR per cell')

hold on

subplot(2,3,2)

plot(time, comR,'LineWidth',1)

title('Amount of ComR per cell')

ylabel('Amount of molecule per cell [MOL/CELL]')

hold on

subplot(2,3,3)

time_dashed=time;

if i>1

time_dashed(1:i*40-40)=NaN;

end

for j=1:length(time/5)

a=0:length(time)-1;

time_dashed(1+i*40+a(j)*200:i*40+160+a(j)*200)=NaN;

end

time_dashed=time_dashed(1:length(time));

plot(time_dashed,comS,'LineWidth',1)

title('Amount of ComS per cell')

hold on

subplot(2,3,4)

plot(time_dashed,comRS,'LineWidth',1)

title('Amount of ComRS per cell')

hold on

subplot(2,3,5)

plot(time_dashed,comX,'LineWidth',1)

title('Amount of ComX per cell')

hold on

h=findobj(gcf,'type','axes');

set([h.XLabel],'string','Time [min]')

set([h.YLabel],'string','Molecules per cell [mol/cell]')

subplot(2,3,6)

plot(time_dashed,degradation,'LineWidth',1)

title('Degradation rate {\it (deg)}')

ylabel('Molecules per cell per minutes [mol/cell.min]')

hold on

end

lgd=legend('0 ComR','WT ComR','3.5 x WT ComR', '6 x WT ComR',...

'8.5 x WT ComR','Location','southeast');

lgd.FontSize = 14;

%% 2. Validation with Range for bimodality with ComS distribution

%%%%%%%%%%%%%%%%%%%%%%%%%%%%%%%%%%%%%%%%%%%%%%%%%%%%%%%%%%%%%%%%%%%%%%%%%%%

%2.1 generating the maximum basal rate value of bS

%%%%%%%%%%%%%%%%%%%%%%%%%%%%%%%%%%%%%%%%%%%%%%%%%%

%Running the simulation for different values of ComS @ 1.27*comR

echt=1.27;

bS_var=linspace(0.0026,0.05,10);

time=0:800;

A=NaN(1,10);

for i=1:10

bS=bS_var(i);

sol3=ode23(@dcomRS_pep,[0,tlast],[0,0,0,0,0,0]);

eval3=deval(sol3,time);

A(i)=max(eval3(4,:));

end

figure

plot(bS_var,A,'ob','MarkerSize',6,'MarkerFaceColor',[0.1,0.4,0.7])

hold on

plot(bS_var,A,'--','color',[0.1,0.6,1])

xlabel('ComS basal rate (b_S)')

ylabel('Maximum ComX amount [mol.cell^{-1}]')

title('Maximum ComX amount in response to ComS basal rate')

xlim([0 0.06])

figure

subplot(2,3,1)

bins=0:0.04:4;

PcomS_gfp = xlsread('dataset1.xlsx','PcomS_gfp','C2:C1841');

PcomS_gfp_log = log10(PcomS_gfp);

h1 = bar(bins,histc(PcomS_gfp_log,bins)/length(PcomS_gfp_log),'histc');

h1.FaceColor = [0.2 0.5 0.3];

xlim([0 4])

ylim([0 0.15])

xticks([ 0 1 2 3 4])

xticklabels({'0','10','10^{2}','10^{3}','10^{4}'})

yticks([0 0.05 0.1 0.15])

yticklabels({'0','5','10','15'})

ylabel('Cell count [%]')

xlabel('Fluorescence Intensity [AU] (log scale)')

title('P_{comS}-gfp^{+} fluorescence distribution')

hold on

%2.2 Generating the cumulative density function for PcomS

%%%%%%%%%%%%%%%%%%%%%%%%%%%%%%%%%%%%%%%%%%%%%%%%%%%%%%%%%

% Getting the noise and fano factor values

noise=(std(PcomS_gfp)^2)/mean(PcomS_gfp)^2;

fano=std(PcomS_gfp)^2/mean(PcomS_gfp);

a=1/noise;

b=fano;

% Generating the probability density function

proba = @(x) ((x.^(a-1)).*exp(-x./b)./(gamma(a)*b^a));

subplot(2,3,2)

c=1:100;

plot(1:100,proba(c),'-','color',[0.2 0.5 0.3])

ylim([0 0.04]);

ylabel('Probability')

xlabel('Fluorescence Intensity [AU]')

title('Gamma probability density function')

hold on

% Generating the cumulative density function

proba_cumul = @(x) integral(proba,0,x);

N=NaN(1,100);

for i=1:100

N(i)=proba_cumul(i);

end

subplot(2,3,3)

plot(1:100,N,'color',[0.2 0.5 0.3])

ylabel('Cummulative Probability')

xlabel('Fluorescence Intensity [AU]')

title('Gamma Cumulative density function')

% 2.3 Generating the range for bimodality

%%%%%%%%%%%%%%%%%%%%%%%%%%%%%%%%%%%%%%%%%

%finding the minimum and maximum values of the distribution with alpha=0.05

find_min=fzero(@(x)proba_cumul(x)-0.05,10);

find_max=fzero(@(x)proba_cumul(x)-0.95,60);

%Calculating the critical bs for competence activation for several ComR values

%and generating bifurcation plot in function of basal rate of ComS for

%different values of ComR

A=NaN(1,20);

B=NaN(10,20);

figure

for i=1:10

echt=i;

for j=1:20

bS_choose=logspace(-3,-1.2,20);

bS=bS_choose(j);

sol4=ode23(@dcomRS_pep,[0,tlast],[0,0,0,0,0,0]);

eval4=deval(sol4,time);

A(j)=max(eval4(4,:));

end

B(i,:)=A;

subplot(2,3,1)

a=-0.09+(0.09*i);

b=(10*i-(i^2))/50;

c=0.6-(0.05*i);

plot(bS_choose,B(i,:),'color',[a,b,c])

hold on

end

title({'ComX maximum concentration in function of ComS basal rate';...

'for various ComR production'})

xlabel('Basal comS synthesis rate b_{S} (log scale)')

ylabel('ComX Maximum concentration [mol.cell^{-1}]')

set(gca, 'XScale', 'log')

xline(0.0026,'--');

xline(0.031,'--');

lgd = legend('1X','2X','3X','4X','5X','6X','7X','8X','9X','10X');

title(lgd,'ComR Fold Increase')

%Getting the critical bs values

C=NaN(1,10);

for i=1:10

for j=2:20

if B(i,j)-B(i,j-1)>50

C(i)=bS_choose(j);

end

end

end

%Scaling critical bs values to I critical

I=C.*(find_max-find_min)./(0.031-0.0026);

%Transforming into percentage

percent=NaN(1,10);

for i=1:10

percent(i)=100-100.*proba_cumul(I(i));

end

%Plot of experimental and simulated percentage computed in relation to ComR

experimental_comR=[1 1.27 1.4 2.62 8.5];

experimental_percent=[0 9.5 21 65 93];

subplot(2,3,2)

plot(1:10,percent,'--','color',[0.8 0 0])

hold on

plot(experimental_comR,experimental_percent,'ob','MarkerSize',6,...

'MarkerFaceColor',[0.1,0.4,0.7])

title({'Percentage of activating cells' ;'in function of ComR abundance'})

xlabel('comR fold increase')

ylabel('Percentage of activated cells [%]')

legend('Simulation','Experimental')

xlim([0 10])

%% Validation with Range for bimodality with ComR distribution

%%%%%%%%%%%%%%%%%%%%%%%%%%%%%%%%%%%%%%%%%%%%%%%%%%%%%%%%%%%%%%%%%%%%%%%%%%%

%3.1 Computing the median value and gamma distribution of ComR

%%%%%%%%%%%%%%%%%%%%%%%%%%%%%%%%%%%%%%%%%%%%%%%%%%%%%%%%%%%%%%

echt=1;

figure

subplot(2,3,1)

bins=0:0.04:4;

PcomR_gfp = xlsread('dataset1.xlsx','PcomR_gfp','C2:C2356');

PcomR_gfp_log = log10(PcomR_gfp);

h1 = bar(bins,histc(PcomR_gfp_log,bins)/length(PcomR_gfp_log),'histc');

h1.FaceColor = [0.2 0.5 0.3];

xlim([0 4])

ylim([0 0.15])

xticks([ 0 1 2 3 4])

xticklabels({'0','10','10^{2}','10^{3}','10^{4}'})

yticks([0 0.05 0.1 0.15])

yticklabels({'0','5','10','15'})

ylabel('Cell count [%]')

xlabel('Fluorescence Intensity [AU] (log scale)')

title('P_{comR}-gfp^{+} fluorescence distribution')

hold on

%getting the noise and fano factor values

noise=(std(PcomR_gfp)^2)/mean(PcomR_gfp)^2;

fano=std(PcomR_gfp)^2/mean(PcomR_gfp);

a=1/noise;

b=fano;

%Generating the probability density function

proba_2 = @(x) ((x.^(a-1)).*exp(-x./b)./(gamma(a)*b^a));

subplot(2,3,2)

c=1:300;

plot(1:300,proba_2(c),'-','color',[0.2 0.5 0.3])

ylim([0 0.015]);

ylabel('Probability')

xlabel('Fluorescence Intensity [AU]')

title('Gamma probability density function')

hold on

%Generating the cumulative density function

proba_cumul_2 = @(x) integral(proba_2,0,x);

N=NaN(1,300);

for i=1:300

N(i)=proba_cumul_2(i);

end

subplot(2,3,3)

plot(1:300,N,'color',[0.2 0.5 0.3])

ylabel('Cummulative Probability')

xlabel('Fluorescence Intensity [AU]')

title('Gamma Cumulative density function')

%3.2 Computing the critical bR value

%%%%%%%%%%%%%%%%%%%%%%%%%%%%%%%%%%%%

%generating the 1.27-fold-change distribution

% (Experimentally, 1.27 fold change correspond to 10% of cells activated)

stdev_127=std(PcomR_gfp)*1.27;

mean_127=mean(PcomR_gfp)*1.27;

noise_127=(stdev_127^2)/mean_127^2;

fano_127=stdev_127^2/mean_127;

a_127=1/noise_127;

b_127=fano_127;

proba_127 = @(x) ((x.^(a_127-1)).*exp(-x./b_127)./(gamma(a_127)*b_127^a_127));

figure

subplot(2,3,1)

c=1:300;

plot(1:300,proba_127(c),'-','color',[0.2 0.5 0.3])

ylabel('Probability')

xlabel('Fluorescence Intensity [AU]')

title('Gamma probability density function')

hold on

%Generating the cumulative density function

proba_cumul_127 = @(x) integral(proba_127,0,x);

N=NaN(1,300);

for i=1:300

N(i)=proba_cumul_127(i);

end

subplot(2,3,2)

plot(1:300,N,'color',[0.2 0.5 0.3])

ylabel('Cummulative Probability')

xlabel('Fluorescence Intensity [AU]')

title('Gamma Cumulative density function')

xline(fzero(@(x)proba_cumul_127(x)-0.9,500),'--');

hold on

% Computing the ComR fold increase corresponding to activation

limit=fzero(@(x)proba_cumul_127(x)-0.9,500); %Fluo intensity corresponding

% to the treshold value

fold=limit/(fzero(@(x)proba_cumul_2(x)-0.5,100)); %Fold change calculated

% in comparison with the "WT" situation corresponding to a "critical" fold

% change for activation

echt=fold;

%3.3 Calculating the percentage of cells

% activated for different fold-change

%%%%%%%%%%%%%%%%%%%%%%%%%%%%%%%%%%%%%%%%%%%%%%

%Generating the fold-change distributions

A=NaN(2,9);

for i=1:10

r=1:1:10;

stdev=std(PcomR_gfp)*r(i);

meani=mean(PcomR_gfp)*r(i);

noise=(stdev^2)/meani^2;

fano=stdev^2/meani;

A(1,i)=1/noise;

A(2,i)=fano;

end

M=NaN(10,3000);

N=NaN(10,3000);

for i=1:3000

for j=1:10

m=j;

proba_var = @(x) ((x.^(A(1,m)-1)).*exp(-x./A(2,m))...

./(gamma(A(1,m))*A(2,m)^A(1,m)));

proba_cumul_var = @(x) integral(proba_var,0,x);

M(j,i)=proba_var(i);

N(j,i)=proba_cumul_var(i);

end

end

figure

for i=1:10

if i<6

plot(axes('Position', [0.05 0.98-(0.18*(i)) 0.12 0.12]),1:3000,N(i,:))

set(gca, 'XScale', 'log')

end

if i>5

plot(axes('Position', [0.2 0.98-(0.18*(i-5)) 0.12 0.12]),1:3000,N(i,:))

set(gca, 'XScale', 'log')

end

tit=sprintf('%d-fold change',i);

title(tit,'FontWeight','normal');

xlim([10 5000]);

xline(limit,'--');

end

h1=text(1,0.001,'Intensity [AU]','FontWeight','bold');

h2=text(0.001,3.5,'Probability','FontWeight','bold');

h4=text(0.37,7.5,'Cumulative density function','FontWeight','bold');

set(h2,'Rotation',90);

%calculating the percentage of activated cells

percent=NaN(1,10);

for i=1:10

m=i;

proba_var = @(x) ((x.^(A(1,m)-1)).*exp(-x./A(2,m))...

./(gamma(A(1,m))*A(2,m)^A(1,m)));

proba_cumul_var = @(x) integral(proba_var,0,x);

percent(i)=-(proba_cumul_var(limit)-1)*100;

end

plot(axes('Position', [0.4 0.3 0.25 0.4]),1:10,percent,'--','color',[0.8 0 0])

hold on

plot(experimental_comR,experimental_percent,'ob','MarkerSize',6,...

'MarkerFaceColor',[0.1,0.4,0.7])

title({'Percentage of activating cells' ;'in function of ComR abundance'})

xlabel('comR fold increase')

ylabel('Percentage of activated cells [%]')

legend('Simulation','Experimental')

disp(' ...done')

%% SENSITIVITY ANALYSIS

disp('Making sensitivity analysis...')

%% 1. Calculating the percentage of activated cells for parameter increase

%%%%%%%%%%%%%%%%%%%%%%%%%%%%%%%%%%%%%%%%%%%%%%%%%%%%%%%%%%%%%%%%%%%%%%%%%%%

% 1.1. generating bifurcation plot in function

% of basal rate of ComS for different values of ComR

%%%%%%%%%%%%%%%%%%%%%%%%%%%%%%%%%%%%%%%%%%%%%%%%%%%%

%Parameter Update

echt=2; % to be in a bistable position

dS = 0.01;

dRS = 0.01;

max_s = 1000;

KcomRS_S = 161;

bS = 0.0026;

krs = 10e-7;

n= 2;

dR = 0.01;

max_x = 60 ;

bX = 0.0107;

dx = 0.2;

KcomRS_X = 322;

Kdeg=1;

deg=1;

kcat=140;

time=0:800;

A=NaN(1,20);

percent=NaN(1,15);

I=1;

% Generating a function calculating the percentage of activating cells

function percent = comp_percent()

for j=1:20

bS_choose=logspace(-3,-1.2,20);

bS=bS_choose(j);

sol4=ode23(@dcomRS_pep,[0,800],[0,0,0,0,0,0]);

eval4=deval(sol4,time);

A(j)=max(eval4(4,:));

end

for j=2:20

if A(j)-A(j-1)>100

C=bS_choose(j);

I=C*(find_max-find_min)/(0.031-0.0026);

end

end

percent=100-100.*proba_cumul(I);

end

for i=1:15

if i==1

dx=dx+0.5*dx;

percent(i)=comp_percent();

elseif i==2

dx=0.2;

bX=bX+0.5*bX;

percent(i)=comp_percent();

elseif i==3

bX=0.0107;

KcomRS_X=KcomRS_X+0.5*KcomRS_X;

percent(i)=comp_percent();

elseif i==4

KcomRS_X=322;

max_x=max_x+max_x*0.5;

percent(i)=comp_percent();

elseif i==5

max_x=60;

dRS=dRS+0.5*dRS;

percent(i)=comp_percent();

elseif i==6

dRS=0.01;

Kdeg=Kdeg+0.501*Kdeg;

percent(i)=comp_percent();

elseif i==7

Kdeg=1;

kcat=kcat+0.5*kcat;

percent(i)=comp_percent();

elseif i==8

kcat=140;

dS=dS+0.5*dS;

percent(i)=comp_percent();

elseif i==9

dS=0.01;

KcomRS_S=KcomRS_S+KcomRS_S*0.5;

percent(i)=comp_percent();

elseif i==10

KcomRS_S=161;

max_s=max_s+0.5*max_s;

percent(i)=comp_percent();

elseif i==11

max_s=1000;

n=n+0.5*n;

percent(i)=comp_percent();

elseif i==12

n=2;

krs=krs+0.5*krs;

percent(i)=comp_percent();

elseif i==13

krs=10e-7;

dR=dR+0.5*dR;

percent(i)=comp_percent();

elseif i==14

dR=0.01;

echt=echt+0.5*echt;

percent(i)=comp_percent();

elseif i==15

echt=2;

percent(i)=comp_percent();

end

end

Kdeg=1;

echt=1;

figure

y=percent./percent(15);

barh(y(1:14))

xline(1,'--');

yticklabels({'d_{X}','b_{X}','K_{X}','max_{X}','d_{RS}','K_{pep}',...

'k_{cat}.pep','d_{S}','K_{S}','max_{S}','n','k_{RS}','d_{R}','b_{R}'})

title('Parameters Sensitivity Analysis')

xlabel('Fold change of the percentage of competence activating cells')

xlim([0 3.5])

disp(' ...done')

%% PREDICTIONS

disp('Making predictions...')

%% 1. Modeling XIP addition vs xip overexpression

%%%%%%%%%%%%%%%%%%%%%%%%%%%%%%%%%%%%%%%%%%%%%%%%%%%%%%%%%%%%%%%%%%%%%%%%%%%

% 1.1 Generating a new system describing XIP addition or overexpression

%%%%%%%%%%%%%%%%%%%%%%%%%%%%%%%%%%%%%%%%%%%%%%%%%%%%%%%%%%%%%%

% A. XIP Addition

%New parameters:

xyl2=0;

re_echt=0;

echt=1;

n=2;

time=0:800;

bS=0.002614;

XIP_added=((10^-9)*(10^-3)*(6.022e23))/X_r(120);% Considering that all the

%XIP is utaken by the cells

% Generating a function describing this uptake

xipi= [XIP_added 127 2 0];

XIP_ = @(x) (xipi(1)*(1/(xipi(3)*sqrt(2*pi)))...

*exp((-((x-xipi(2))/xipi(3)).^2)./2).*(1+erf(xipi(4)...

*((x-xipi(2))./xipi(3))./(sqrt(2)))));

function deriv_pep = dcomRS_pep_xip(t,statevar)

comR = statevar(1) ;

comS = statevar(2) ;

comRS = statevar(3) ;

comX = statevar(4);

degradation = statevar(5);

XIP = statevar(6);

dcomR = echt*ProdR_(t)-dR*comR-krs*((comR*comS)^n);

dcomS = max_s*((comRS/(comRS+KcomRS_S))+bS)-dS*comS-krs*((comR*comS)^n)...

-kcat*deg*((comS)/(comS+Kdeg))+re_echt*XIP_(t)+xyl2;

dcomRS = (1/2)*krs*((comR*comS)^n)-dRS*comRS;

dcomX = max_x*((comRS/(comRS+KcomRS_X))+bX)-dx*comX;

ddegradation = kcat*deg*((comS)/(comS+Kdeg))-degradation;

dXIP=re_echt*XIP_(t)-XIP;

deriv_pep = [dcomR;dcomS;dcomRS;dcomX;ddegradation;dXIP] ;

end

xyl2=0;

b=NaN(1,5);

A=NaN(5,801);

B=NaN(5,801);

figure

hold on

for i=1:5

c=1.015*10^(-4);

a=[5*c 10*c 20*c 40*c 80*c];

re_echt=a(i);

sol5=ode23(@dcomRS_pep_xip,[0,800],[0,0,0,0,0,0]);

eval5=deval(sol5,time);

degradation=eval5(5,:);

comR=eval5(1,:);

comS=eval5(2,:);

comRS=eval5(3,:);

comX=eval5(4,:);

XIP=eval5(6,:);

b(i)= sum(XIP);

A(i,:)=XIP;

B(i,:)=degradation;

time_dashed1=time;

if i>1

time_dashed1(1:i*40-40)=NaN;

end

for j=1:length(time/3)

a=0:length(time)-1;

time_dashed1(1+i*40+a(j)*120:i*40+80+a(j)*120)=NaN;

end

time_dashed1=time_dashed1(1:length(time));

time_dashed2=time;

if i>1

time_dashed2(1:i*40-40)=NaN;

end

for j=1:length(time/2)

a=0:length(time)-1;

time_dashed2(1+i*40+a(j)*80:i*40+40+a(j)*80)=NaN;

end

time_dashed2=time_dashed2(1:length(time));

if i<4

subplot(2,3,1)

plot(time_dashed1, comR,'LineWidth',1)

title('Amount of ComR per cell')

ylabel('Molecules per cell [mol.cell^{-1}]')

hold on

subplot(2,3,2)

plot(time_dashed1,comS,'LineWidth',1)

title('Amount of ComS per cell')

ylabel('Molecules per cell [mol.cell^{-1}]')

hold on

subplot(2,3,3)

plot(time_dashed1,comRS,'LineWidth',1)

title('Amount of ComRS per cell')

ylabel('Molecules per cell [mol.cell^{-1}]')

hold on

subplot(2,3,4)

plot(time_dashed1,comX,'LineWidth',1)

title('Amount of ComX per cell')

ylabel('Molecules per cell [mol.cell^{-1}]')

hold on

end

if i>3

subplot(2,3,1)

plot(time_dashed2, comR,'LineWidth',1)

title('Amount of ComR per cell')

ylabel('Molecules per cell [mol.cell^{-1}]')

hold on

subplot(2,3,2)

plot(time_dashed2,comS,'LineWidth',1)

title('Amount of ComS per cell')

ylabel('Molecules per cell [mol.cell^{-1}]')

hold on

subplot(2,3,3)

plot(time_dashed2,comRS,'LineWidth',1)

title('Amount of ComRS per cell')

ylabel('Molecules per cell [mol.cell^{-1}]')

hold on

subplot(2,3,4)

plot(time_dashed2,comX,'LineWidth',1)

title('Amount of ComX per cell')

ylabel('Molecules per cell [mol.cell^{-1}]')

hold on

end

subplot(2,3,5)

plot(time,XIP,'LineWidth',1)

title('Rate of XIP importation')

ylabel('Molecules per cell per minute [mol.cell^{-1}.min^{-1}]')

hold on

subplot(2,3,6)

plot(time,degradation,'LineWidth',1)

title('ComS Degradation rate')

ylabel('Molecules per cell per minute [mol.cell^{-1}.min^{-1}]')

hold on

h=findobj(gcf,'type','axes');

set([h.XLabel],'string','Time [min]')

lab1=sprintf('{%0.0f}',b(1));lab2=sprintf('{%0.0f}',b(2));

lab3=sprintf('{%0.0f}',b(3));

lab4=sprintf('{%0.0f}',b(4));lab5=sprintf('{%0.0f}',b(5));

end

lgd=legend(lab1,lab2,lab3,lab4,lab5,'Location','southeast');

lgd.FontSize = 10;

title(lgd,{'Total XIP_{S} uptaken';'[mol.cell^{-1}]'},'FontSize',10)

plot(axes('Position', [0.51 0.25 0.1 0.175]),time,A(1,:),time,A(2,:),...

time,A(3,:),time,A(4,:),time,A(5,:))

xlim([120 140])

hold on

plot(axes('Position', [0.79 0.18 0.1 0.175]),time,B(1,:),time,B(2,:),...

time,B(3,:),time,B(4,:),time,B(5,:))

xlim([120 140])

% B. Computing for xip overexpression

%New parameters:

re_echt=0;

time=0:800;

xyl2=0;

b=NaN(1,5);

A=NaN(5,801);

B=NaN(5,801);

figure

hold on

for i=1:5

a=linspace(10,50,5);

xyl2=a(i);

sol6=ode23(@dcomRS_pep_xip,[0,800],[0,0,0,0,0,0]);

eval6=deval(sol6,time);

degradation=eval6(5,:);

comR=eval6(1,:);

comS=eval6(2,:);

comRS=eval6(3,:);

comX=eval6(4,:);

XIP=eval6(6,:);

b(i)= a(i);

A(i,:)=XIP;

B(i,:)=degradation;

time_dashed1=time;

if i<4

if i>1

time_dashed1(1:i*40-40)=NaN;

end

time_dashed1(i*40:200)=NaN;

for j=1:5

a=[200 320 440 560 680];

if i>1

time_dashed1(200:200+i*40-40)=NaN;

end

time_dashed1(a(j)+i*40:a(j)+80+i*40)=NaN;

end

time_dashed1=time_dashed1(1:length(time));

subplot(2,3,1)

plot(time_dashed1, comR,'LineWidth',1)

title('Amount of ComR per cell')

ylabel('Molecules per cell [mol.cell^{-1}]')

hold on

end

time_dashed2=time;

if i>3

time_dashed2(1:i*40-40)=NaN;

for j=1:6

a=[200 300 400 500 600 700];

time_dashed2(a(j)+(i-3)*50:a(j)+50+(i-3)*50)=NaN;

end

time_dashed2=time_dashed2(1:length(time));

subplot(2,3,1)

plot(time_dashed2, comR,'LineWidth',1)

title('Amount of ComR per cell')

ylabel('Molecules per cell [mol.cell^{-1}]')

hold on

end

time_dashed3=time;

if i<4

for j=1:6

a=0:150:800;

time_dashed3(a(j)+i*50:a(j)+100+i*50)=NaN;

end

time_dashed3=time_dashed3(1:length(time));

subplot(2,3,2)

plot(time_dashed3,comS,'LineWidth',1)

title('Amount of ComS per cell')

ylabel('Molecules per cell [mol.cell^{-1}]')

hold on

end

if i>3

subplot(2,3,2)

plot(time,comS,'LineWidth',1)

title('Amount of ComS per cell')

ylabel('Molecules per cell [mol.cell^{-1}]')

hold on

end

time_dashed4=time;

if i<4

for j=1:6

a=0:150:800;

time_dashed4(a(j)+i*50:a(j)+100+i*50)=NaN;

end

time_dashed4=time_dashed4(1:length(time));

subplot(2,3,3)

plot(time_dashed4,comRS,'LineWidth',1)

title('Amount of ComRS per cell')

ylabel('Molecules per cell [mol.cell^{-1}]')

hold on

end

if i>3

time_dashed5=time;

for j=1:6

a=[200 300 400 500 600 700];

time_dashed5(a(j)+(i-3)*50:a(j)+50+(i-3)*50)=NaN;

end

time_dashed5=time_dashed5(1:length(time));

subplot(2,3,3)

plot(time_dashed5,comRS,'LineWidth',1)

title('Amount of ComRS per cell')

ylabel('Molecules per cell [mol.cell^{-1}]')

hold on

end

time_dashed6=time;

if i<4

for j=1:6

a=0:150:800;

time_dashed6(a(j)+i*50:a(j)+100+i*50)=NaN;

end

time_dashed6=time_dashed6(1:length(time));

subplot(2,3,4)

plot(time_dashed6,comX,'LineWidth',1)

title('Amount of ComX per cell')

ylabel('Molecules per cell [mol.cell^{-1}]')

hold on

end

if i>3

time_dashed7=time;

for j=1:6

a=[250 350 450 550 650 750];

time_dashed7(a(j)+(i-3)*50:a(j)+50+(i-3)*50)=NaN;

end

time_dashed7=time_dashed7(1:length(time));

subplot(2,3,4)

plot(time_dashed7,comX,'LineWidth',1)

title('Amount of ComX per cell')

ylabel('Molecules per cell [mol.cell^{-1}]')

hold on

end

if i<4

subplot(2,3,5)

plot(time,degradation,'LineWidth',1)

title('ComS Degradation rate')

ylabel('Molecules per cell per minute [mol.cell^{-1}.min^{-1}]')

hold on

end

if i>3

time_dashed8=time;

for j=1:3

a=[200 300 400];

time_dashed8(a(j)+(i-3)*50:a(j)+50+(i-3)*50)=NaN;

end

time_dashed8=time_dashed8(1:length(time));

subplot(2,3,5)

plot(time_dashed8,degradation,'LineWidth',1)

title('ComS Degradation rate')

ylabel('Molecules per cell per minute [mol.cell^{-1}.min^{-1}]')

hold on

end

h=findobj(gcf,'type','axes');

set([h.XLabel],'string','Time [min]')

lab1=sprintf('{%0.0f}',b(1));lab2=sprintf('{%0.0f}',b(2));

lab3=sprintf('{%0.0f}',b(3));

lab4=sprintf('{%0.0f}',b(4));lab5=sprintf('{%0.0f}',b(5));

end

lgd=legend(lab1,lab2,lab3,lab4,lab5,'Location','southeast');

lgd.FontSize = 10;

title(lgd,{'xip overexpression synthesis rate';'[mol.cell^{-1}.min^{-1}]'},...

'FontSize',10)

disp(' ...done')

disp(' Finally done this time !')

toc

end
